# Competitive endogenous RNA network and pathway-based analysis of LncRNA single-nucleotide polymorphism in myasthenia gravis

**DOI:** 10.1038/s41598-021-03357-x

**Published:** 2021-12-14

**Authors:** Tianfeng Wang, Si Xu, Huixue Zhang, Xiaoyu Lu, Shuang Li, Li Liu, Xiaotong Kong, Hongyu Gao, Xu Wang, Shangwei Ning, Jianjian Wang, Lihua Wang

**Affiliations:** 1 Department of Neurology, The Second Affiliated Hospital of Harbin Medical University, 246 Xuefu Road, Harbin, 150081 Heilongjiang China; 2 College of Bioinformatics Science and Technology, Harbin Medical University, Harbin, 150081 China

**Keywords:** Data mining, Neurological disorders

## Abstract

Myasthenia gravis (MG) is a complex neurological autoimmune disease with a pathogenetic mechanism that has yet to be elucidated. Emerging evidence has revealed that genes, non-coding RNAs and genetic variants play significant roles in the pathogenesis of MG. However, the molecular mechanisms of single nucleotide polymorphisms (SNPs) located on lncRNAs could disturb lncRNA-mediated ceRNA regulatory functions still unclear in MG. In this study, we collated 276 experimentally confirmed MG risk genes and 192 MG risk miRNAs. We then constructed a lncRNA-mediated ceRNA network for MG based on multi-step computational strategies. Next, we systematically integrated risk pathways and identified candidate SNPs in lncRNAs for MG based on data acquired from public databases. In addition, we constructed a pathway-based lncRNA-SNP mediated network (LSPN) that contained 128 lncRNAs targeting 8 MG risk pathways. By analyzing network, we propose a latent mechanism for how the “lncRNA-SNP-mRNA-pathway” axis affects the pathogenesis of MG. Moreover, 25 lncRNAs and 51 SNPs on lncRNAs were extracted from the “lncRNA-SNP-mRNA-pathway” axis. Finally, functional analyses demonstrated lncRNA-SNPs mediated ceRNA regulation pairs associated with MG participated in the MAPK signaling pathway. In summary, we constructed MG-specific lncRNA-SNPs mediated ceRNA regulatory networks based on pathway in the present study, which was helpful to elucidate the roles of lncRNA-SNPs in the pathogenesis of MG and provide novel insights into mechanism of lncRNA-SNPs as potential genetic risk biomarkers of MG.

## Introduction

Myasthenia gravis (MG) is an autoimmune disease that is mediated by acetylcholine receptor antibodies which attack the postsynaptic membrane at the neuromuscular junction, thus resulting in muscle weakness and fatigue^1^. Low density lipoprotein receptor-related protein 4 and muscle-specific kinase (MuSK) antibodies have also been implicated in MG^2,3^. The relative role of genetic susceptibility in the development of MG has been widely studied; however, the precise molecular mechanisms involved have yet to be fully elucidated^4,5^.

Long non-coding RNA (lncRNA) and microRNA (miRNA) are both non-coding forms of RNA and play key roles in a range of biological functions, including the regulation of immune responses^6,7^. Recently, researchers discovered that the abnormal expression of lncRNA XLOC_003810 in the thymus aggravates Th17/Treg cell dysregulation in patients with MG, thus suggesting that lncRNAs may regulate certain key biological processes^8^. A growing body of evidence now suggests that the abnormal expression of miRNA may contribute to the initiation and progression of MG by binding to the 3′-UTR of target genes. A recent study also put forward a new theory involving competing endogenous RNA (ceRNA) in which RNA molecules that are targeted by common miRNAs could compete with miRNA response elements (MREs), thereby indirectly regulating each other^9^. Furthermore, there appears to be a growing body of evidence to support the fact that lncRNAs function as miRNA “sponges” to compete with mRNAs and thus regulate their activity^10^. Disturbances in the regulatory mechanisms associated with ceRNA can cause immunological and other diseases^11^. For instance, in our previous study, we found that lncRNA SNHG16 played a crucial role in the immune progression of MG by acting as a ceRNA for miRNA let-7c-5p, thus resulting in reduced expression levels of interleukin 10, its endogenous target gene^12^.

Over recent years, a number of single-nucleotide polymorphisms (SNPs) have been reported to be involved in the pathogenic mechanisms underlying MG^13,14^. For example, a previous genome-wide association study (GWAS) identified certain disease-associated SNPs in the human genome and has become a powerful tool with which to investigate biological process and the genetic architecture of complex diseases^15^. Surprisingly, very few of the SNPs identified thus far are located in protein-coding regions^16^. Instead, the majority of the SNPs identified thus far are located in non-coding regions, including miRNAs and lncRNAs. Consequently, it is more difficult to explain the biological significance of these SNPs^17^. An accumulating body of evidence now indicates that risk variants in lncRNAs could lead to a range of diseases by influencing the expression of protein-coding genes or the expression levels of lncRNAs and miRNAs^18^. Consequently, it is conceivable that lncRNA-associated SNPs could affect the regulation of lncRNAs. Furthermore, the presence of risk-associated SNPs on lncRNAs may lead to alterations on the miRNA binding sites and result in the gain or loss of ceRNA interactions^19^. In addition, the mechanisms of SNPs induced the dysregulation of ceRNA include: (i) When the SNP occurs on the miRNA binding site might destroy the combination of lncRNA-miRNA, which lead to the loss of ceRNA interactions; (ii) The SNP occurs on miRNA binding site might produce a new miRNA binding site, which lead to establish the new ceRNA interactions^20,21^. For example, the functional rs664589 polymorphism in lncRNA MALAT1 is known to alter the miR-194-5p binding site and result in increased levels of MALAT1 expression, thus reducing the risk of colorectal cancer^22^. Furthermore, the rs710886 SNP in lncRNA PCAT1 is known to lead to the loss of miRNA-145 binding sites, thus resulting in increased levels of PCAT1 expression and the inhibition of tumor cell invasion and proliferation^23^. Despite advances in our knowledge of how SNPs might influence lncRNAs, we know very little about how lncRNAs, SNPs, and miRNAs, are associated with the specific genes and molecular pathways that have been implicated in MG. Therefore, the identification and characterization of SNPs will help us to discover regulatory mechanisms in MG that involve lncRNAs and SNPs and may help us to develop new diagnoses and treatments for MG.

In the present study, we carried out bioinformatics analysis of human lncRNAs featuring SNPs that may disturb miRNA binding sites. Next, we systematically identified candidate functional lncRNAs featuring SNPs that are associated with MG by creating a global lncRNA-mediated ceRNA network for MG and identifying risk pathways by adopting a multi-step computational approach (refer to the flowchart shown in Supplementary Fig. S1). Our results may help to reveal lncRNAs featuring SNPs that might exert influence over lncRNA-miRNA-mRNA interactions and provide us with new concepts for studying the progression and pathogenesis of MG. In conclusion, we identified and characterized the effects of lncRNAs that feature SNPs and affect ceRNA regulation pairs in MG; these may represent new biomarkers and therapeutic targets for patients with MG.

## Results

### Construction of the lncRNA-mediated ceRNA network

**Figure 1:**
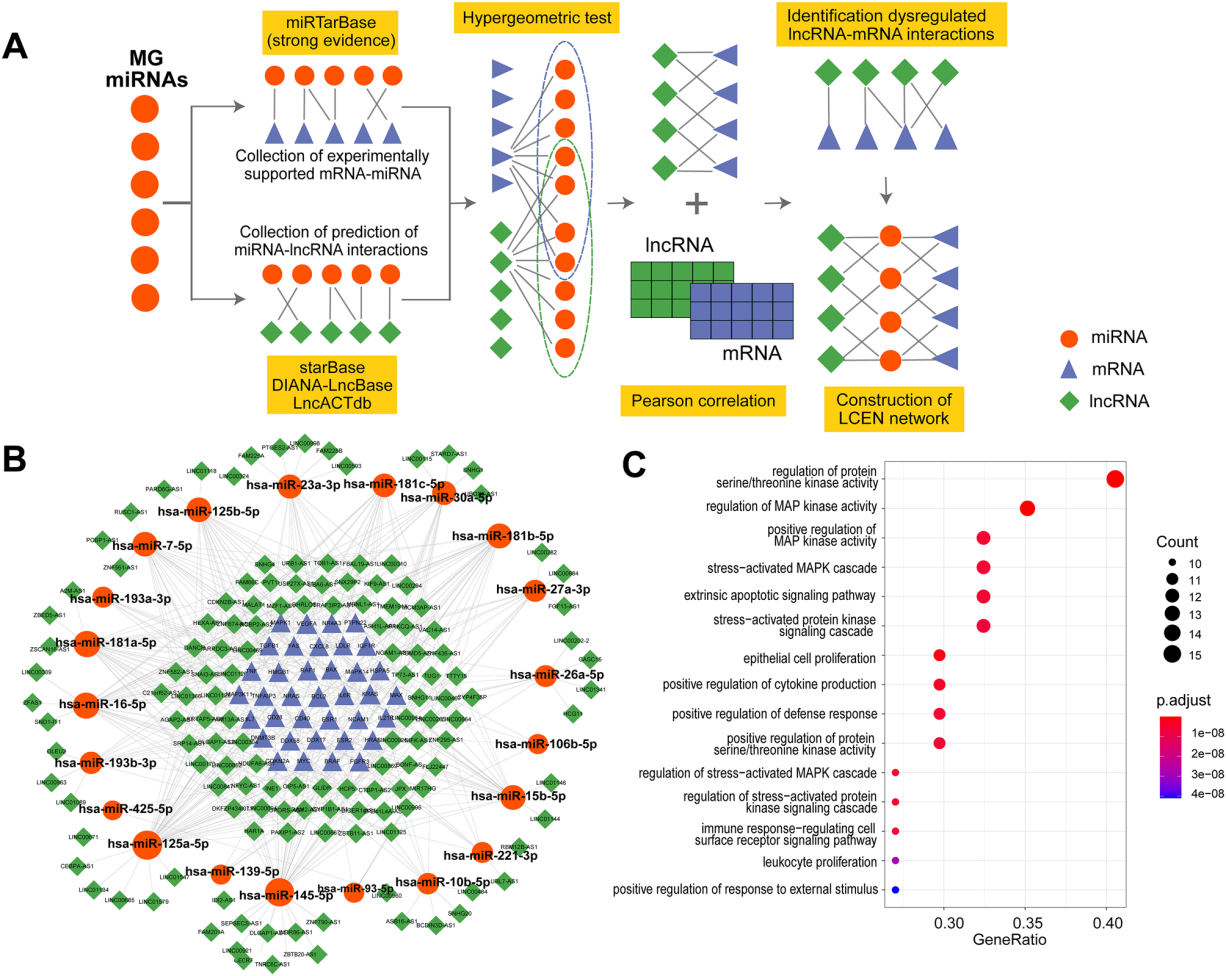
The construction and characteristics of a LCEN. (**A**) An integrative pipeline used to construct the LCEN by merging dysregulated lncRNA-mRNA ceRNA interactions based on the ceRNA hypothesis. (**B**) The creation of a LCEN network for MG demonstrated pair bonds among lncRNAs, miRNAs, and mRNAs. Blue triangles represent mRNAs, red circles represent miRNAs, green rhombi represent lncRNAs, and lines represent their regulatory interactions. (**C**) GO annotation of mRNAs that were co-expressed with mRNAs.

It is well established that lncRNAs can act as “sponges” for miRNAs in order to regulate mRNAs^10^. To investigate the role of lncRNA-mediated ceRNA regulation in MG, we constructed a LCEN network via a multi-step approach (Fig. 1A). The LCEN generated contained 21 miRNAs, 37 genes, 140 lncRNAs, and 388 edges (Fig. 1B). Moreover, for each lncRNA, we computed the number of primary lncRNA-miRNA interactions and secondary miRNA-mRNA interaction pairs of; these represent the number of lncRNAs that are linked with miRNAs and mRNAs (Table S1). LINC00667, PAXIP1-AS2 and HCP5, exhibited higher total numbers of lncRNA-miRNA and miRNA-mRNA interactions, and were considered as hub lncRNAs; these lncRNAs played crucial roles in the network. Then, we carried out functional GO annotation analysis based on co-expressed mRNAs. GO annotation of the MG co-expressed gene set was significantly enriched in certain categories, including immune response, the regulation of cytokine production, neuronal apoptotic processes, and lymphocyte proliferation; these processes were in accord with the immunological mechanisms associated with MG pathogenesis. We showed the top 15 functional GO terms (biological processes) that may play important roles in the pathogenesis of MG (Fig. 1C). These findings revealed the fundamental characteristics of co-expressed lncRNAs that are involved in the pathogenesis of MG.

### Enrichment analysis of risk pathways for MG

**Figure 2:**
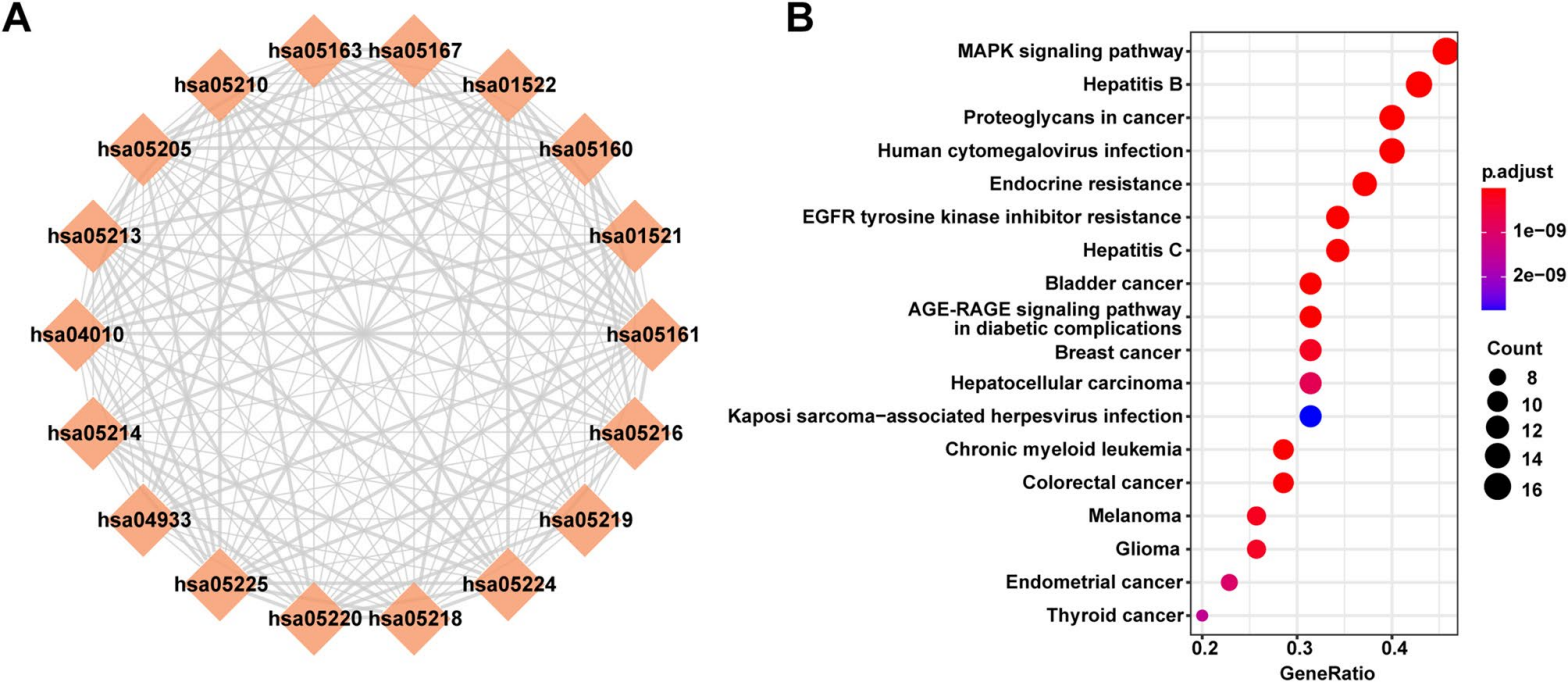
MG-associated risk pathways. (**A**) A pathway crosstalk network identifying strongly correlated pathways in risk pathways for MG. The red rhombus stands for one pathway, the line between two pathways represents a significant correlation between two pathways. (**B**) KEGG enrichment analysis showing the top 18 pathways involving the co-expression of lncRNAs and risk genes for MG (P < 0.01).

Next, we performed KEGG pathway enrichment analysis using co-expressed mRNAs and identified the top 18 risk pathways for MG (Table 1, detailed information outlined in Table S2). We also identified the correlation between risk pathways for MG by analyzing the intersection between each two pathways based on hypergeometric tests; this allowed us to establish an MG- associated pathway crosstalk network (Fig. 2A). This network suggested that most of the enriched pathways were particularly correlated with the other part. In addition, we identified certain enriched KEGG pathways (*P* < 0.01); the MAPK signaling pathway was the most highly enriched pathway (Fig. 2B), which implicated potential significant role of MAPK signaling pathway in MG. For example, the MAPK signaling pathway is known to contribute to pathological conditions of the nervous system as well as autoimmune diseases by regulating immunity^24^. Moreover, we found that the co-expressed genes in LCEN were mainly enriched in oncogenic and infectious pathways, which implies that we can use these oncogenic pathways as well as infectious pathways to functionally characterize MG. As we know, MG is an autoimmune disease related to thymoma, therefore some of pathways were involved in cancer. We also identified several GO terms (biological processes) by annotating the co-expressed risk genes for MG, including the activation of innate immune response, T cell activation, and the regulation of adaptive immunity; these processes were in accord with the immune pathogenesis of MG (Table S3). Collectively, these findings demonstrated that the risk pathways for MG may play an important synergistic role in the pathogenesis of and identified the fundamental characteristics of co-expressed lncRNAs in the regulation of MG.

**Table 1:** Enriched KEGG pathways of MG risk genes.

Pathway term	FDR	Pathway map
hsa05219: Bladder cancer	9.17E−16	Human disease (cancers: specific types)
hsa05161: Hepatitis B	3.66E−15	Human disease (Infectious disease: viral)
hsa01522: Endocrine resistance	3.86E−15	Human disease (Drug resistance: antineoplastic)
hsa01521: EGFR tyrosine kinase inhibitor resistance	1.00E−14	Human disease (Drug resistance: antineoplastic)
hsa04010: MAPK signaling pathway	4.95E−13	Environmental Information Processing (Signal transduction)
hsa05205: Proteoglycans in cancer	1.28E−12	Human diseases (Cancer: overview)
hsa05163: Human cytomegalovirus infection	4.01E−12	Human disease (Infectious disease: viral)
hsa04933: AGE-RAGE signaling pathway in diabetic complications	4.37E−12	Human disease (Endocrine and metabolic disease)
hsa05220: Chronic myeloid leukemia	8.94E−12	Human disease (cancers: specific types)
hsa05160: Hepatitis C	1.94E−11	Human disease (Infectious disease: viral)
hsa05210: Colorectal cancer	2.64E−11	Human disease (cancers: specific types)
hsa05218: Melanoma	1.86E−10	Human disease (cancers: specific types)
hsa05224: Breast cancer	1.97E−10	Human disease (cancers: specific types)
hsa05214: Glioma	2.33E−10	Human disease (cancers: specific types)
hsa05225: Hepatocellular carcinoma	7.35E−10	Human disease (cancers: specific types)
hsa05213: Endometrial cancer	9.72E−10	Human disease (cancers: specific types)
hsa05216: Thyroid cancer	1.50E−09	Human disease (cancers: specific types)
hsa05167: Kaposi sarcoma-associated herpesvirus infection	2.75E−09	Human disease (Infectious disease: viral)

### Construction of a pathway-based lncRNA-SNP mediated network

**Figure 3:**
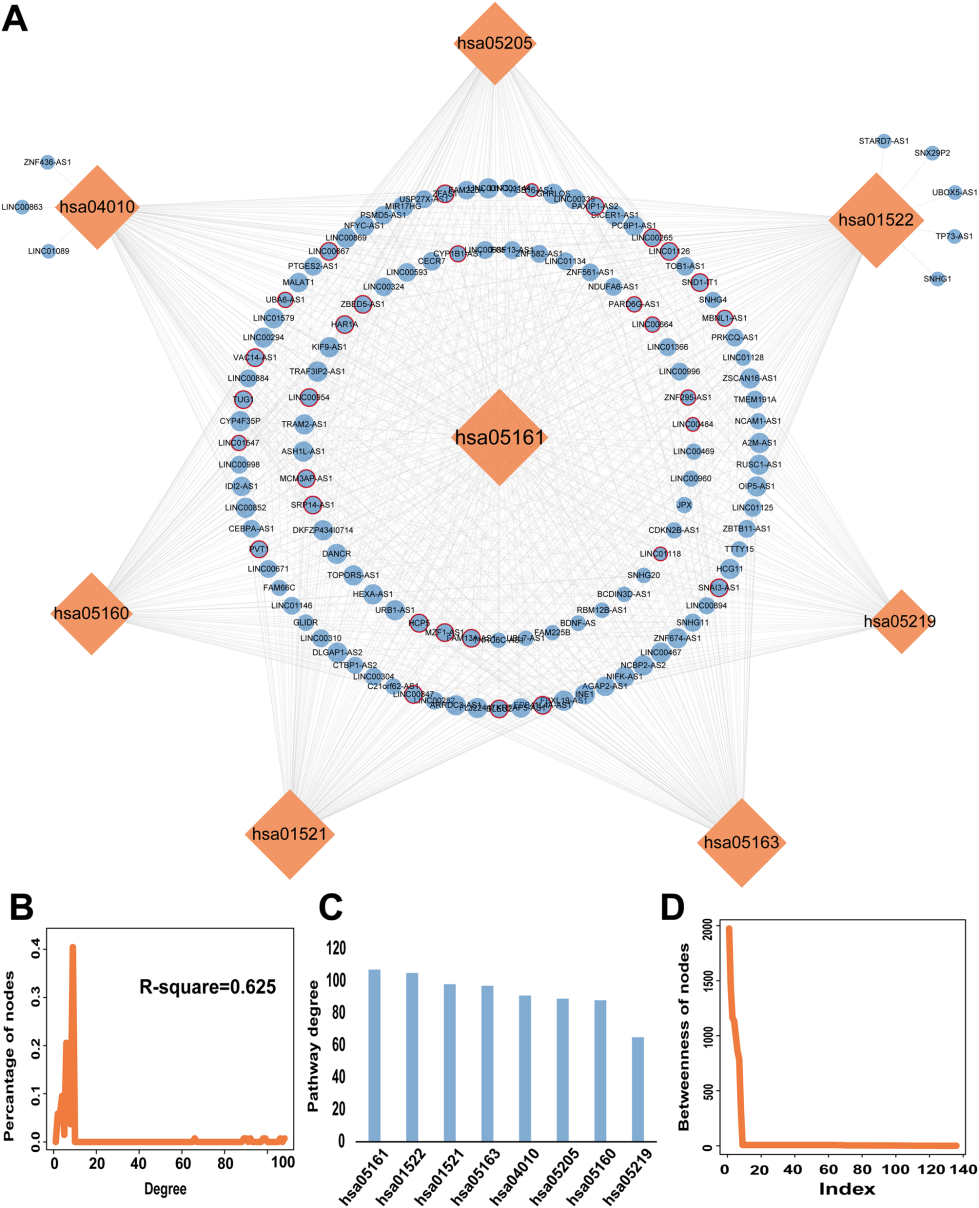
Topological properties of the LSPN. (**A**) The LSPN featured 8 risk pathways, 128 lncRNAs and identified numerous lncRNA-SNPs. The orange rhombi and blue circles represent pathways and lncRNAs, respectively; their size is a direct reflection of their degrees. The lncRNA-SNPs contain miRNA binding sites that are represented by red circles around a blue circle. (**B**) The distribution of node degrees within the global LSPN network. (**C**) Bar plots showing the distribution of degrees within the pathway. (**D**) The distribution of node betweenness in the LSPN.

Based on hypergeometric testing and co-expression analysis, we identified 359 lncRNA-miRNA-mRNA interactions, involving 37 risk genes, 140 lncRNAs, and 21 miRNAs. By evaluating lncRNA-mediated regulation and the potential roles of lncRNA SNPs in MG susceptibility, we constructed a LSPN to objectively illustrate the potential impact of lncRNA-SNPs on MG at the pathway level (Table S4); this involved ceRNA theory and a multi-step approach (Fig. 3A). Our analysis created a LSPN that contained 128 lncRNAs, 30 lncRNA-SNPs within the miRNA binding sites that may disrupt the regulation of lncRNAs, miRNAs, mRNAs, in eight risk pathways for MG. These lncRNA-SNPs could exert regulatory functionality on these risk pathways.

Next, we dissected the topological characteristics of the LSPN, including the degree distribution and topological coefficient. We found that the degree distribution of all nodes followed a power law distribution in the LSPN that was defined by f(x) = 20.481x^−0.636^ (Fig. 3B). Next, we determined the degree distribution for the pathway (Fig. 3C) and identified specific lncRNAs showing the best top linkage. The top five pathways were “hsa05161: hepatitis B”, “hsa01522: endocrine resistance”, “hsa01521: endocrine resistance”, “hsa05163: human cytomegalovirus infection”, and “hsa04010: MAPK signaling pathway”; collectively, these pathways showed the highest degrees of connection with the majority of the lncRNAs. We also calculated the betweenness of nodes within the LSPN (Fig. 3D); these analyses indicated that the higher betweenness of a node, the more important the node was in maintaining the tight connectivity of the network. Collectively, these findings revealed that these lncRNA-SNP mediated pathways were involved in the pathogenesis of MG.

### Dissection of the potential mechanisms underlying the regulation of MG by lncRNA-SNPs

**Figure 4:**
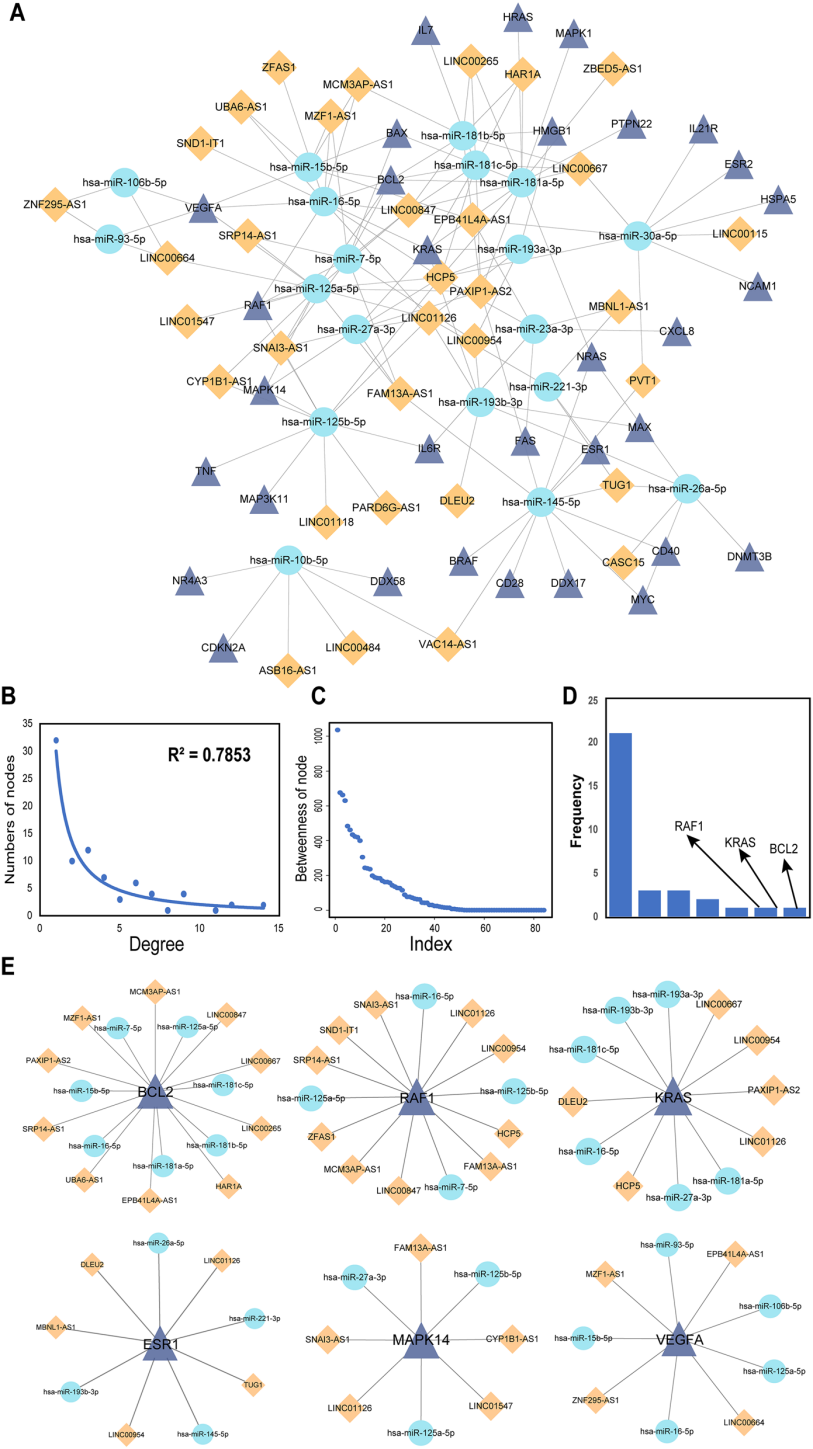
The lncRNA-SNP mediated ceRNA network and basic characteristics. (**A**) The lncRNA-SNP mediated ceRNA network in myasthenia gravis. Purple triangles represent genes, the blue circles represent miRNAs, and the orange rhombi represents lncRNAs; the lines represent regulatory relationships. (**B**,**C**) The degree distribution and betweenness distribution of the network. (**D**) The degree distribution of genes. (**E**) The sub-network of the top six genes ranked by degree.

In order to understand how lncRNA-SNPs affect the regulation of miRNAs and mRNAs, we established a lncRNA-SNP mediated ceRNA network that contained 84 nodes and 151 edges (Fig. 4A). There were 32 lncRNAs, 33 genes, and 19 miRNAs, in the lncRNA-SNP mediated ceRNA network, thus indicating that lncRNAs occupied a large proportion of all nodes and might play a key role within the network. Then, we identified the degree and betweenness distribution. The degree of all nodes conformed to a power law distribution that was defined by f(x) = 30.083x^-1.161^ (R^2^ = 0.7853 Fig. 4B); this approximated a scale-free network.

**Figure 5:**
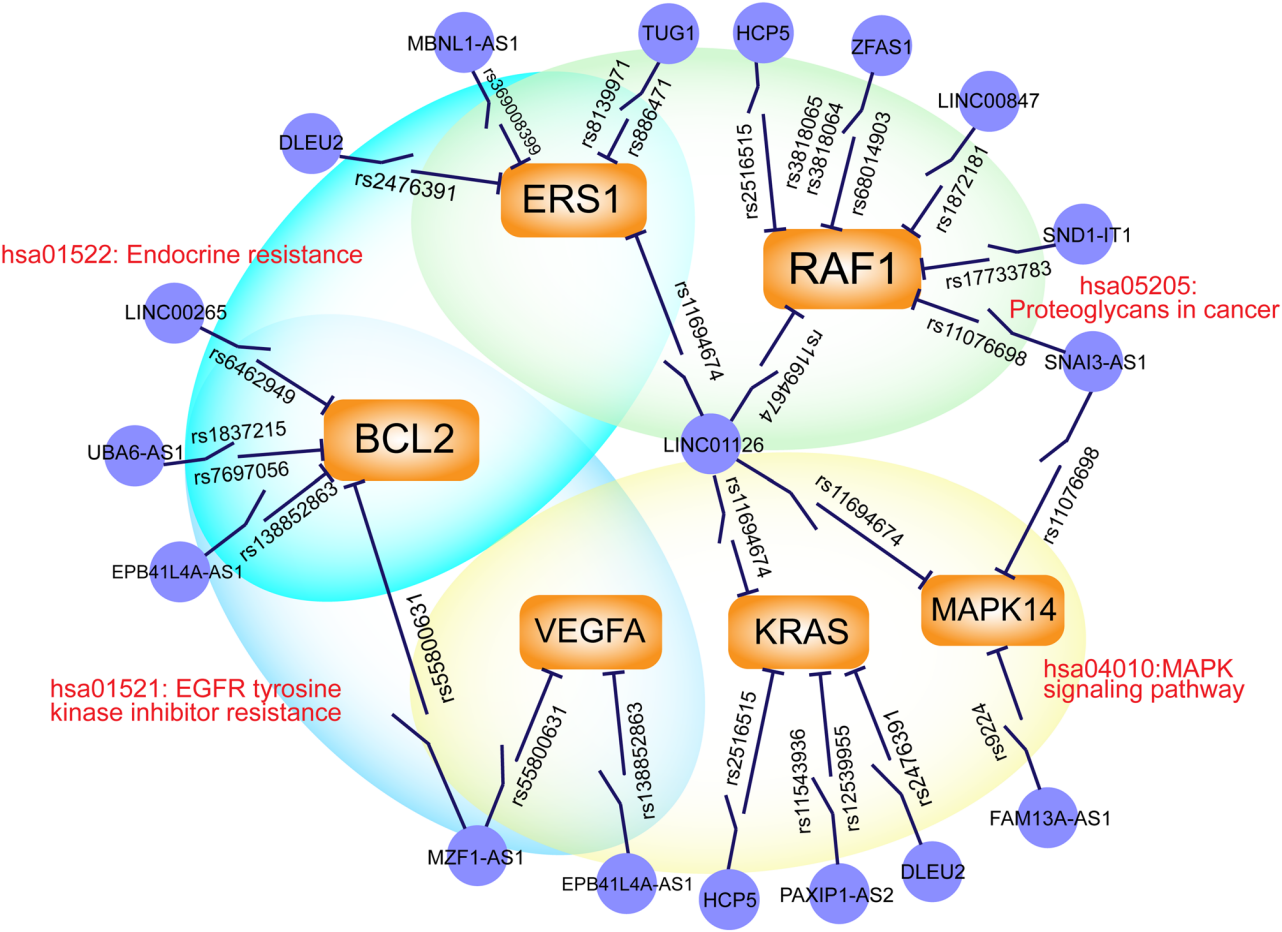
A schematic diagram of the lncRNA-SNP-gene-pathway axis. The orange rectangles and purple circles represent high-risk genes and their regulatory lncRNAs, respectively. The lines represent the interactions between lncRNAs and genes. The switch symbol shown on the line represent lncRNA-SNPs. The large peripheral circles represent pathways containing enriched genes.

We also calculated the betweenness of the nodes (Fig. 4C) and determined the degree distribution of genes (Fig. 4D) in the lncRNA-SNP mediated ceRNA network. Based on the LSPN, lncRNA-SNP mediated ceRNA network, and by considering knowledge relating to the pathogenesis of MG, we identified six high-risk genes (*BCL2*, *KRAS*, *MAPK14*, *VEGFA*, *RAF1*, *ESR1*) and for risk pathways for MG. Next, we constructed sub-networks for these risk gene-miRNA pairs and their linked lncRNA SNPs (Fig. 4E). The presence of lncRNA-SNPs in risk genes were appropriate for the pathogenesis of MG because the regulation of these risk genes has been demonstrated in MG^25–27^. We identified 32 lncRNAs that contained risk SNPs and may act as ceRNAs to regulate MG risk gene-miRNA pairs at the pathway level. These lncRNAs are known to participate in the development of a range of cancers^28–30^, and also play roles in the immune response^31^; they are also known to improve neuronal injury^32^ and inhibit the proliferation of bone marrow-derived mesenchymal stem cells^33^. These findings suggest that certain lncRNA-SNP mediated ceRNAs and pathways play key roles in the immunological pathogenesis of MG and exhibit complex features. Next, we inferred the biological mechanisms of a potential “lncRNA-SNP-risk gene-pathway” axis; our analysis demonstrated that lncRNA-SNPs could perturb the pathways associated with MG by influencing the regulation of gene-miRNA pairs (Fig. 5). Six high-risk genes were shown to be regulated by 15 lncRNAs which were all potentially able to influence the expression and function of risk genes. We demonstrated that *BCL2*, *KRAS*, *MAPK14*, *VEGFA*, *RAF1*, and ESR1, were the top six high-risk genes in the pathogenesis of MG and could be regulated by numerous lncRNAs and lncRNA-SNPs and influence several crucial risk pathways for MG.

### The lncRNA-SNP mediated ceRNA regulation pairs in MG were associated with significant biological functions and the MAPK signaling pathway

**Figure 6:**
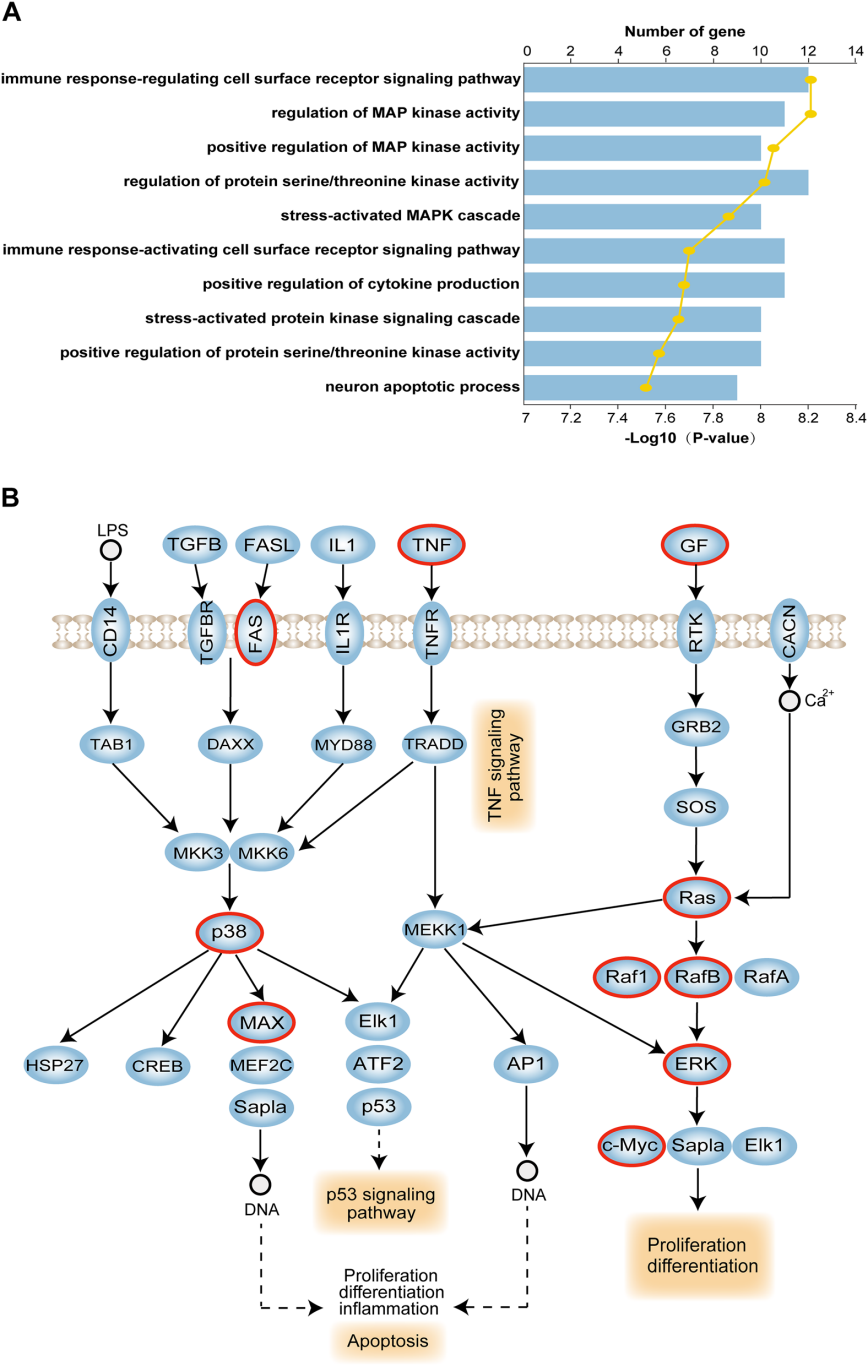
GO enrichment analyses of genes associated with dysregulated lncRNA-SNP mediated ceRNA interaction pairs and risk pathways for MG. (**A**) GO terms enriched for genes in lncRNA-SNP mediated ceRNA interaction pairs in MG, ranked by -log10(P-value). (**B**) The MAPK signaling pathway and dysregulated genes in lncRNA-SNP mediated ceRNA interaction pairs.

GO enrichment analyses were carried out using genes that were co-expressed in the lncRNA-SNP mediated ceRNA network in MG. These genes were particularly enriched in some pivotal immune biological functions, including the immune response-regulating cell surface receptor signaling pathway, the regulation of MAP kinase activity, the positive regulation of cytokine production, and the process of apoptosis in neurons (Fig. 6A). Abnormal activation of the immune response in MG was previously associated with the production of proinflammatory cytokines that play an important role in the pathogenesis of MG^34^. The dissection of risk pathways further demonstrated that the MAPK signaling pathway (hsa04010) plays an extremely significant role in the pathogenesis of MG and featured the highest density of SNPs. The MAPK signaling pathway is an intracellular signaling pathway that plays a key role in various cellular functions, including cell proliferation, differentiation, and migration^24^. In this current study, 13 co-expressed risk genes in the lncRNA SNPs mediated ceRNA regulation pairs were involved in this pathway (Fig. 6B). For example, the *MAPK14* (p38) gene plays a key role in the MAPK signaling pathway and has been found to regulate neuronal activity and reduce the functionality of neural stem cells^35^. MAPK14 formed dysregulated lncRNA-SNP mediated ceRNA regulation pairs with lncRNA-01126 and hsa-miR-125a-5p. In summary, these results suggest that these lncRNA-SNP mediated ceRNA regulation pairs are associated with MG and are able to exert action by regulating the MAPK signaling pathway.

## Discussion

MG is a complex disease and the pathogenetic mechanisms and autoimmune processes involved are still unclear. An extensive body of evidence now supports the fact that lncRNAs play a regulatory role in the immune system and can exert influence in numerous autoimmune diseases and MG^7,36^. The identification of SNPs in lncRNA-mediated ceRNA regulation pairs in MG by considering relevant risk pathways could help us to illustrate the potential roles of lncRNA-SNPs in the progression of MG. In the present study, computational analysis provided an integrated catalog of lncRNA-SNP mediated ceRNA interactions in risk pathways associated with MG. We systematically screened candidate functional lncRNA-SNPs and their potential mechanisms by considering our existing knowledge of MG. First, we constructed an LCEN based on ceRNA theory using a computational approach. To further verify reliability of our results, we tested weighted gene co-expression analysis (WGCNA) based on expression profiles with the WGCNA R package. As a result, in the turquoise module which had the highest number of genes, there were 101 common shared between Pearson correlation coefficients analysis and WGCNA analysis (Supplementary Fig. S2). These findings enhanced the credibility of our results. The LCEN featured 21 miRNAs, 37 genes, and 140 lncRNAs. GO functional annotation analysis further revealed that these lncRNAs may play key roles in the development of MG. Furthermore, we constructed and dissected an LSPN associated with MG which could help us to investigate the potential pathogenesis of MG by considering genetic variation and post-transcriptional regulation. We considered lncRNA-SNPs as a key regulatory system and by identifying MG-related risk genes and risk pathways, we constructed a “lncRNA-SNP-mRNA-risk pathway” axis that was based on public data resources and ceRNA theory. The functional analyses highlighted the importance of the MAPK signaling pathway. The activation of the MAPK/ERK pathway is known to be associated with enhanced anti-oxidative capacity and the prevention of cell death^37^.

We not only demonstrated that the ceRNA network for MG could be influenced by SNPs, we also identified some important biological functions that were related to SNP-mediated ceRNAs and risk pathways for MG. Functional pathway analysis for MG provided us with an enhanced understanding of how the immune system plays a role in the pathogenesis of MG. Some of the top 18 risk pathways for MG were associated with immunity; in particular, we identified three pathways that were associated with “infectious diseases”, thus suggesting that certain mechanisms of infection may be involved in the pathogenesis of MG. A previous study revealed that toll-like receptor (TLR)7 and TLR9 may play a key role in the inflammation caused in the thymus by infection with Epstein-Barr virus (EBV), thus suggesting that the EBV-induced expression of TLR7/9 may participate in the onset and maintenance of the immune response during the-intra thymic pathogenesis of MG^38^.

Furthermore, we selected 128 lncRNA-mediated ceRNAs that may play a regulatory role in certain risk pathways for MG. Our present findings suggested that the lncRNA might result in the abnormal expression of genes which might lead to the dysregulation of key pathways, thus leading to the occurrence and progression of MG. Previous studies have found that HCP5 was shown to regulate neuroblastoma cells activation and promoted neuroblastoma progression by acting as ceRNA to bind with miR-186-5p and regulate MAP3K2 expression^39^. Wang et al. found that lncRNA EPB41L4A-AS1 could function as the ceRNA regulator in the pathogenesis of NSCLC^30^. In addition, lncRNA has been investigated as part of the ceRNA regulator in various complex disease, but its molecular mechanism in autoimmune diseases of the nervous system was still unclear. Though the lncRNAs mentioned above that have been validated by previous work are not specific for MG, these results demonstrate that our strategy for identifying lncRNAs involved in the development of MG is reliable. However, there is little known about the functions of the other lncRNAs in the LSPN network, which may thus be novel regulators of the pathogenesis of MG. Moreover, we provided an example of how these regulators might be involved in the pathogenesis of MG by acting on targets and pathways. As we know, lncRNA regulates the gene expression by acting as ceRNA. As a gene target, mRNA received regulation through this ceRNA, which could be enriched in different pathways, such as MAPK signaling pathway, proteoglycans in cancer and EGFR tyrosine kinase inhibitor resistance. This indicates an ongoing relationship between the mechanisms responsible for cell activation and proliferation that influence the immune response in the autoimmune conditions of MG.

By analyzing the LSPN and considering the mechanisms associated with MG, we identified the potentially significant roles of the MAPK signaling pathway (hsa04010), six high-risk genes (*BCL2*, *KRAS*, *MAPK14*, *VEGFA*, *RAF1*, and *ESR1*), and associated lncRNA-SNPs in MG. By mining lncRNA-SNPs in high-risk genes for MG, we demonstrated the four most significant potential mechanisms for lncRNA-SNP-gene-pathway effects: as follows: rs138852863/EPB41L4A-AS1 → VEGFA → hsa04010 (MAPK signaling pathway); rs2516515/HCP5 → RAF1 → hsa05205 (proteoglycans in cancer); rs17177030/MCM3AP-AS1 → BCL2 → hsa01521 (EGFR tyrosine kinase inhibitor resistance)/hsa01522 (Endocrine resistance); rs2476391/ DLEU2 → ESR1 → hsa05205 (proteoglycans in cancer). Furthermore, the MAPK signaling pathway was identified as a key risk pathway for MG; within this pathway, VEGFA plays an important regulatory role in crosstalk and cell survival. In a previous study, Uzawa et al. demonstrated that VEGFA was significantly upregulated in patients with MG and thymoma and acts as a key regulator by inhibiting apoptosis and inducing cell proliferation^26^. In addition, VEGFA, rs138852863/EPB41L4A-AS1, and hsa-miR-16-5p formed a dysregulated SNP-mediated ceRNA in MG, thus suggesting that VEGFA might exert action in the MAPK signaling pathway by forming ceRNA in patients with MG. Our results indicated that the genes and pathways mediated by lncRNA-SNPs in ceRNA could create a dynamic balance in the mechanisms underlying the progression of MG.

In conclusion, we constructed and analyzed SNP-mediated ceRNAs in MG based on a computational approach. We created ta catalog of risk genes and miRNAs for MG, identified risk pathways for MG, identified lncRNA-SNPs that could affect lncRNA-miRNA-mRNA interactions and regulate risk pathways for MG. We also constructed a LSPN and identified a “lncRNA-SNP-mRNA-pathway” axis. In the present study, we focused on these SNPs in miRNA binding sites of lncRNAs, integrated MG related risk genes, miRNAs, ceRNA network and risk pathways multi-dimensionally by using public data resources. Our intention was to show that these SNPs in miRNA binding sites of lncRNAs are potential functional variants, which could be candidates in ‘wet’ lab experimental designs. However, SNPs caused gain or loss of function in MREs might affect ceRNA efficiency related factors still lack direct proof of being involved in MG, all this indirect evidence could offer for researchers to explore the association of lncRNA SNPs in MG. These could be a valuable complement to experimental studies and assist with future studies of lncRNA functions in MG. However, several limitations also exist in our study. Firstly, because the miRNAs that we manually collected were extracted from multiple immune cell types, a great deal of genes and lncRNAs might be generated during the process of integrating the data, which might expand the range of the results but decrease the credibility unfortunately. Moreover, because of the large number of genes and lncRNAs, we filtered by hypergeometric test and PCC analysis, trying to make the data more accurate. Secondly, lncRNAs and ceRNA hypothesis have not been extensively investigated in MG, so most lncRNAs that have not been previously reported in this context require validation. Finally, we constructed the LSPN and identified several critical lncRNA SNPs and pathways, but the actual relationships between their need to be verified experimentally in future studies. Furthermore, in vitro and in vivo experiments would be necessary to confirm the potential roles of lncRNA and lncRNA SNPs in MG pathogenesis.

Our study revealed an efficient method to investigate the molecular mechanisms underlying the actions of lncRNA-SNPs in the pathogenesis of MG. We focused on lncRNA SNP in MG for the first time and will provide important clues for the investigation of gene regulation by lncRNA SNP in the pathogenesis of MG based on ceRNA network and pathway analysis. Collectively, our findings provide novel insight and supporting evidence for the further validation and investigation of lncRNA-SNPs and ceRNAs in the pathogenesis of MG.

## Methods

### Risk genes for MG and the acquisition of miRNA

Data relating to risk genes were acquired by two methods. First, we identified all existing research publications focusing on MG-associated genes from the PubMed database (http://www.ncbi.nlm.nih.gov/pubmed) by exploring literature published prior to 1st May 2020 and using the following terms [myasthenia gravis (MeSH Terms) and English (Language)]. We restricted our literature searches to “*Homo sapiens*”. Then, we manually selected MG-associated genes that met the following standards, as described in our previous study^40^: (i) the risk gene was indicated in more than 5 MG samples (including blood samples and thymic tissue samples); (ii) the risk gene had been detected using credible experimental methods (such as microarrays, RT-PCR, ELISA, or western blotting); (iii) the risk gene was differentially expressed in samples from patients with MG. Secondly, we also collected genes from the Online Mendelian Inheritance in Man^41^, DisGeNET^42^, and the Genetic Association Database^43^. In a similar manner, we also literature search data relating to risk miRNA for MG using the keywords ‘microRNA’ or ‘miRNA’ or ‘miR’ and ‘myasthenia gravis' in PubMed. Finally, we total collected 192 MG risk miRNAs from 31 literatures published online and the detailed information of MG risk miRNAs which we collected was summarized in (Table S5).

### MiRNA-lncRNA and miRNA-mRNA interaction data

Experimentally verified mRNA-miRNA interactions were downloaded from miRTarBase (Release 7.0)^44^. By adopting this database, we retained high-confidence functional mRNA-miRNA interactions that were supported by luciferase reporter assays or western blotting data. mRNA-miRNA interactions were further filtered using the risk genes and risk miRNAs for MG, as described above. We also extracted miRNA-lncRNA interactions from starBase^45^, DIANA-LncBase^46^, and LncACTdb^47^; these are databases that feature high throughput and experimentally validated miRNA-lncRNA interactions. These miRNA-lncRNA intersections were filtered by risk miRNAs for MG. Then, we removed repetitive miRNA-lncRNA entries and retained the remaining data as candidate miRNA-lncRNA interactions.

### Cumulative hypergeometric testing

We applied the following two steps in the identification of the competing mRNA-lncRNA interactions pairs. Firstly, to identify competing mRNA-lncRNA interaction pairs that shared the same miRNA, we used hypergeometric tests to identify competing pairs based on the common miRNAs of any pair of mRNAs and lncRNAs^48^. The *P* value was computed using the following formula given in Eq. (1):

1 $$P=1-\sum_{k=0}^{x}\frac{\left(\genfrac{}{}{0pt}{}{m}{k}\right)\left(\genfrac{}{}{0pt}{}{N-m}{n-k}\right)}{\left(\genfrac{}{}{0pt}{}{N}{n}\right)}$$

For each interaction pair, *N* denotes the total number of risk miRNAs for MG, *n* and *m* represent the number of miRNAs that were associated with one mRNA and one lncRNA, respectively, and *x* represents the number of miRNAs that overlap between mRNA and lncRNA. mRNA-lncRNA interaction pairs with a *P*-value < 0.01 were considered to be significant interactions.

### LncRNA-mRNA co-expression analysis and the construction of LCEN

Next, to identify lncRNA-mRNA interaction pairs, we performed co-expression correlations for lncRNA-mRNA by calculating Pearson correlation coefficients (PCC) based on the expression of lncRNA and mRNAs. The mRNA and lncRNA expression data were downloaded from the dbGaP database (https://www.ncbi.nlm.nih.gov/projects/gap/cgi-bin/study.cgi?study_id=phs000424.v7.p2)^49^. The threshold for mRNA-lncRNA interaction pairs was set to a PCC > 0.5 and a *P*-value < 0.01. Both mRNA and lncRNA were co-expressed and shared common miRNAs for merging into a competing triplet. We constructed a lncRNA-mediated ceRNA network (LCEN) after identifying all lncRNA-miRNA-mRNA competing triplets. Then, we visualized the network by applying Cytoscape software, in which nodes represented genes, miRNAs, and lncRNAs; the edges represent interactions.

### Functional enrichment analysis

We carried out Kyoto Encyclopedia of Genes and Genomes (KEGG) (https://www.kegg.jp/) pathway enrichment analysis^50^ to identify the potential roles of the lncRNAs identified. To do this, we applied the clusterProfiler package^51^; this is a functional annotation tool in the R package. We also used the clusterProfiler package to carry out gene ontology (GO) annotation^52^ for the co-expression of lncRNAs. Adjusted *P*-values with a false discovery rate (FDR) < 0.01 were considered to be significantly enriched function annotations (for KEGG pathways and GO terms).

### Acquisition of lncRNA/SNP data and the construction of a LSPN and a lncRNA-SNP mediated ceRNA network

We used the LnCeVar database^19^ to identify lncRNA/SNP data that could lead to alterations in miRNA binding sites, thus leading to the gain or loss of miRNA function^19^. The LnCeVar database is a database featuring high-quality manual curation from the published literature and high-throughput experimentally supported genomic variations that lead to the abnormal regulation of the lncRNA-variation-ceRNA network. We used the database to screen for candidate lncRNA-SNPs that may potentially exert influence on miRNA-lncRNA interactions. Then, we constructed a LSPN to objectively demonstrate the latent impact of lncRNA-SNPs on MG at the pathway level. Finally, we generated a lncRNA-SNP mediated ceRNA network to allow us to dissect the potential mechanisms involved in the regulation of MG. These networks were visualized same way as the LCEN network.

### Topological features of the LSPN and lncRNA-SNP mediated ceRNA network for MG

We analyzed a range of topological features for all nodes in the pathway-based lncRNA-SNP mediated network and the lncRNA-SNP mediated ceRNA network, including degree and betweenness distribution. Cytoscape software (v 3.8.1 http://www.cytoscape.org/) was used for the visualization of all the networks.

## Supplementary Information

Supplementary Figure 1.

Supplementary Figure 2.

Supplementary Legends.

Supplementary Table S1.

Supplementary Table S2.

Supplementary Table S3.

Supplementary Table S4.

Supplementary Table S5.
