# Supplementary figures and images for: Competitive endogenous RNA network and pathway-based analysis of LncRNA single-nucleotide polymorphism in myasthenia gravis
Source: Sci Rep. 2021 Dec 14;11:23920. doi: 10.1038/s41598-021-03357-x (PMC8671434; doi:10.1038/s41598-021-03357-x)

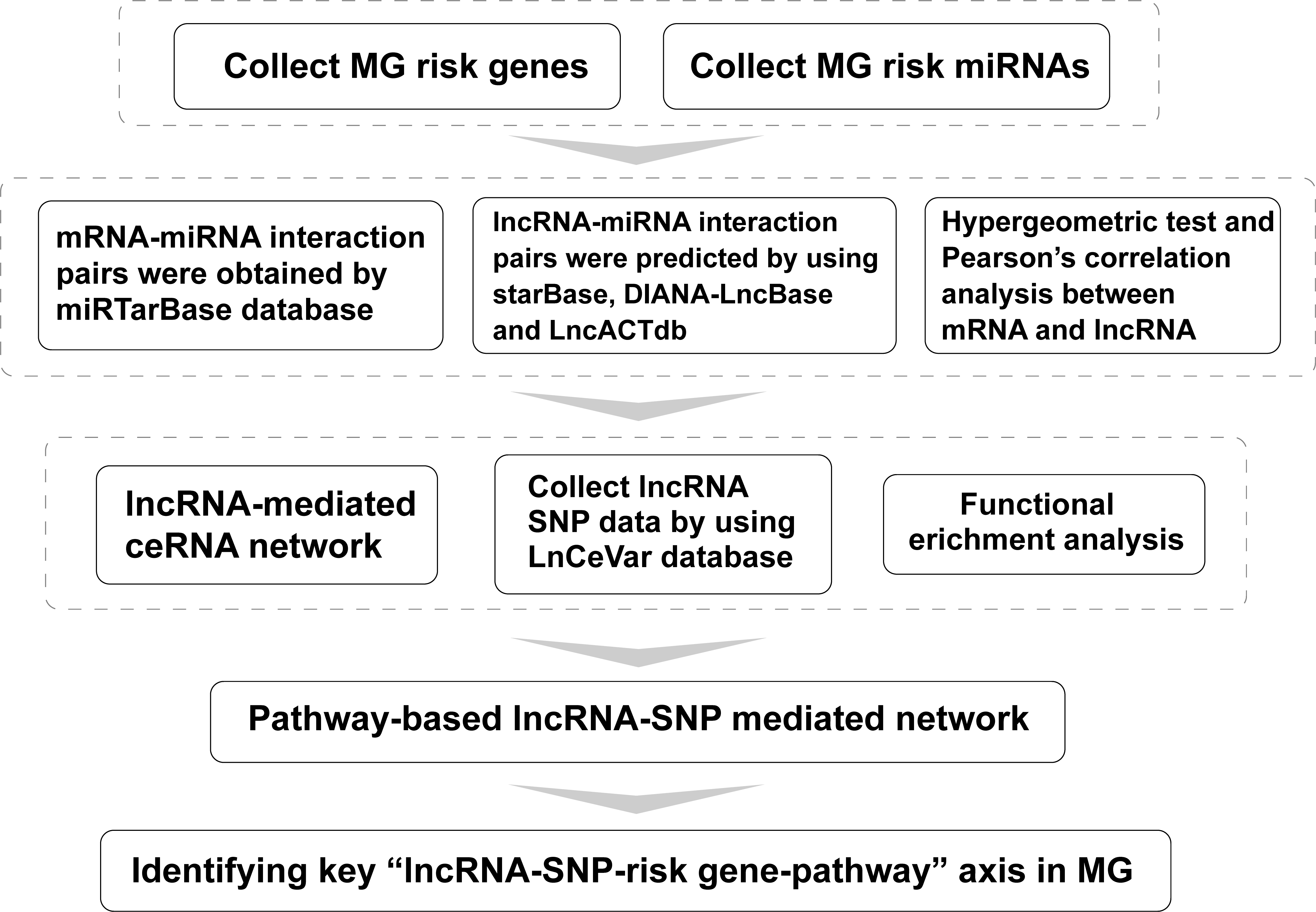

Supplement: Supplementary file 1 — Supplementary Figure 1. [file 41598_2021_3357_MOESM1_ESM.tif]

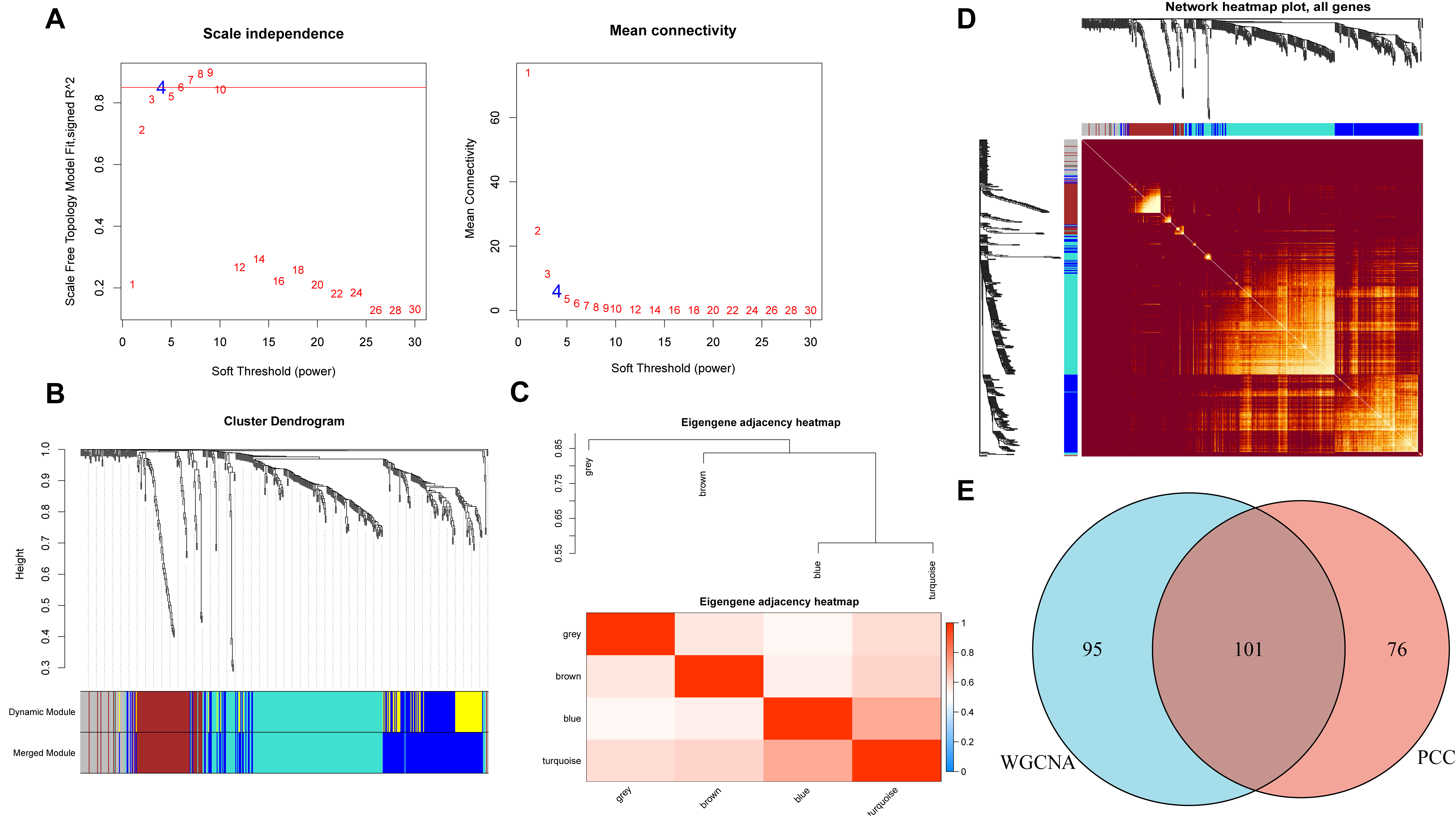

Supplement: Supplementary file 2 — Supplementary Figure 2. [file 41598_2021_3357_MOESM2_ESM.tif]
