# Supplementary material for: Competitive endogenous RNA network and pathway-based analysis of LncRNA single-nucleotide polymorphism in myasthenia gravis
Source: Sci Rep. 2021 Dec 14;11:23920. doi: 10.1038/s41598-021-03357-x (PMC8671434; doi:10.1038/s41598-021-03357-x)
Supplement: Supplementary file 3 — Supplementary Legends. [file 41598_2021_3357_MOESM3_ESM.docx]

**Supplementary Figure 1 The flowchart of methods.**

**Supplementary Figure 2 Construction of co-expression modules by WGCNA.** (A) Analysis of the network topology for different soft threshold powers. (B) Cluster dendrogram of the co-expression network modules. (C) Visualization of network using the heatmap plot. (D) Heatmap depicting the topological overlap matrix (TOM) of lncRNA-mRNA co-expression network modules. A redder background demonstrates a higher module correlation. (E) Venn diagram of the turquoise module and PCC. Blue circle indicates the lncRNA-mRNA co-expression in turquoise module, red circle indicates the lncRNA-mRNA co-expression of PCC analysis.
