# Supplementary material for: Competitive endogenous RNA network and pathway-based analysis of LncRNA single-nucleotide polymorphism in myasthenia gravis
Source: Sci Rep. 2021 Dec 14;11:23920. doi: 10.1038/s41598-021-03357-x (PMC8671434; doi:10.1038/s41598-021-03357-x)
Supplement: Supplementary file 4 — Supplementary Table S1. [file 41598_2021_3357_MOESM4_ESM.docx]

**Table S1 The number of lncRNA–miRNA, miRNA–mRNA regulation pairs for each lncRNA in the LCEN**

| LncRNA name | Number of  lncRNA-miRNA  regulation pairs | Number of  miRNA-mRNA  regulation pairs | Total |
| --- | --- | --- | --- |
| HCP5 | 9 | 41 | 50 |
| GLIDR | 6 | 35 | 41 |
| OIP5-AS1 | 6 | 30 | 36 |
| PAXIP1-AS2 | 6 | 30 | 36 |
| LINC01126 | 7 | 27 | 34 |
| LINC00894 | 6 | 27 | 33 |
| CTBP1-AS2 | 6 | 24 | 30 |
| DKFZP434I0714 | 5 | 24 | 29 |
| LINC00847 | 5 | 22 | 27 |
| LINC00469 | 5 | 21 | 26 |
| KRTAP5-AS1 | 4 | 21 | 25 |
| DANCR | 3 | 21 | 24 |
| FAM13A-AS1 | 4 | 19 | 23 |
| SNAI3-AS1 | 4 | 19 | 23 |
| TTTY15 | 4 | 19 | 23 |
| C21orf62-AS1 | 4 | 18 | 22 |
| JPX | 5 | 17 | 22 |
| LINC00667 | 4 | 18 | 22 |
| ZNF436-AS1 | 3 | 19 | 22 |
| ZNF582-AS1 | 4 | 18 | 22 |
| CDKN2B-AS1 | 4 | 17 | 21 |
| LINC00294 | 4 | 17 | 21 |
| ZNF674-AS1 | 4 | 17 | 21 |
| EPB41L4A-AS1 | 4 | 16 | 20 |
| LINC01366 | 3 | 17 | 20 |
| ARRDC3-AS1 | 3 | 16 | 19 |
| DICER1-AS1 | 3 | 16 | 19 |
| GHRLOS | 3 | 16 | 19 |
| KIF9-AS1 | 3 | 16 | 19 |
| LINC00852 | 3 | 16 | 19 |
| HEXA-AS1 | 3 | 15 | 18 |
| MIR17HG | 2 | 16 | 18 |
| FBXL19-AS1 | 3 | 14 | 17 |
| HAR1A | 3 | 14 | 17 |
| LINC00265 | 3 | 14 | 17 |
| TRAF3IP2-AS1 | 3 | 14 | 17 |
| LINC00310 | 3 | 13 | 16 |
| TUG1 | 3 | 13 | 16 |
| CYP1B1-AS1 | 2 | 13 | 15 |
| FAM66C | 3 | 12 | 15 |
| INE1 | 2 | 13 | 15 |
| LINC00467 | 3 | 12 | 15 |
| LINC01128 | 3 | 12 | 15 |
| MCM3AP-AS1 | 3 | 12 | 15 |
| MZF1-AS1 | 3 | 12 | 15 |
| NCAM1-AS1 | 2 | 13 | 15 |
| BDNF-AS | 2 | 12 | 14 |
| CYP4F35P | 3 | 11 | 14 |
| LINC00926 | 2 | 12 | 14 |
| MALAT1 | 2 | 12 | 14 |
| PVT1 | 2 | 12 | 14 |
| PSMD5-AS1 | 2 | 11 | 13 |
| SNHG11 | 2 | 11 | 13 |
| TMEM191A | 2 | 11 | 13 |
| TOB1-AS1 | 2 | 11 | 13 |
| URB1-AS1 | 2 | 11 | 13 |
| VAC14-AS1 | 2 | 11 | 13 |
| DLGAP1-AS2 | 2 | 10 | 12 |
| LINC00869 | 2 | 10 | 12 |
| TRAM2-AS1 | 3 | 9 | 12 |
| AGAP2-AS1 | 2 | 9 | 11 |
| FLJ22447 | 2 | 9 | 11 |
| LINC00954 | 3 | 8 | 11 |
| LINC01125 | 2 | 9 | 11 |
| NCBP2-AS2 | 2 | 9 | 11 |
| NDUFA6-AS1 | 2 | 9 | 11 |
| PRKCQ-AS1 | 2 | 9 | 11 |
| TP73-AS1 | 2 | 9 | 11 |
| UBA6-AS1 | 2 | 9 | 11 |
| LINC00173 | 2 | 8 | 10 |
| LINC00304 | 2 | 8 | 10 |
| LINC00664 | 3 | 7 | 10 |
| NFYC-AS1 | 2 | 8 | 10 |
| SNHG4 | 2 | 8 | 10 |
| SRP14-AS1 | 2 | 8 | 10 |
| TOPORS-AS1 | 2 | 8 | 10 |
| A2M-AS1 | 1 | 8 | 9 |
| CECR7 | 1 | 8 | 9 |
| DLGAP1-AS1 | 1 | 8 | 9 |
| FAM201A | 1 | 8 | 9 |
| IDI2-AS1 | 1 | 8 | 9 |
| LINC00921 | 1 | 8 | 9 |
| LINC01118 | 1 | 8 | 9 |
| PARD6G-AS1 | 1 | 8 | 9 |
| SEPSECS-AS1 | 1 | 8 | 9 |
| SNX29P2 | 2 | 7 | 9 |
| TNRC6C-AS1 | 1 | 8 | 9 |
| WDR86-AS1 | 1 | 8 | 9 |
| ZBED5-AS1 | 1 | 8 | 9 |
| ZBTB11-AS1 | 2 | 7 | 9 |
| ZBTB20-AS1 | 1 | 8 | 9 |
| ZNF790-AS1 | 1 | 8 | 9 |
| ZSCAN16-AS1 | 1 | 8 | 9 |
| ASH1L-AS1 | 2 | 5 | 7 |
| NIFK-AS1 | 2 | 5 | 7 |
| CEBPA-AS1 | 1 | 5 | 6 |
| LINC00339 | 1 | 5 | 6 |
| LINC00671 | 1 | 5 | 6 |
| LINC00685 | 1 | 5 | 6 |
| LINC01134 | 1 | 5 | 6 |
| LINC01547 | 1 | 5 | 6 |
| LINC01579 | 1 | 5 | 6 |
| MBNL1-AS1 | 2 | 4 | 6 |
| SND1-IT1 | 1 | 5 | 6 |
| ZFAS1 | 1 | 5 | 6 |
| CASC15 | 1 | 4 | 5 |
| HCG11 | 1 | 4 | 5 |
| LINC00115 | 1 | 4 | 5 |
| LINC00202-2 | 1 | 4 | 5 |
| LINC01144 | 1 | 4 | 5 |
| LINC01146 | 1 | 4 | 5 |
| LINC01341 | 1 | 4 | 5 |
| SNHG1 | 1 | 4 | 5 |
| STARD7-AS1 | 1 | 4 | 5 |
| UBOX5-AS1 | 1 | 4 | 5 |
| ASB16-AS1 | 1 | 3 | 4 |
| BCDIN3D-AS1 | 1 | 3 | 4 |
| DLEU2 | 1 | 3 | 4 |
| FAM225B | 1 | 3 | 4 |
| FAM225A | 1 | 3 | 4 |
| FGF13-AS1 | 1 | 3 | 4 |
| LINC00282 | 1 | 3 | 4 |
| LINC00324 | 1 | 3 | 4 |
| LINC00484 | 1 | 3 | 4 |
| LINC00593 | 1 | 3 | 4 |
| LINC00863 | 1 | 3 | 4 |
| LINC00884 | 1 | 3 | 4 |
| LINC00996 | 2 | 2 | 4 |
| LINC00998 | 1 | 3 | 4 |
| LINC01089 | 1 | 3 | 4 |
| PCBP1-AS1 | 1 | 3 | 4 |
| PTGES2-AS1 | 1 | 3 | 4 |
| RUSC1-AS1 | 1 | 3 | 4 |
| SNHG20 | 1 | 3 | 4 |
| USP27X-AS1 | 1 | 3 | 4 |
| ZNF295-AS1 | 2 | 2 | 4 |
| ZNF561-AS1 | 1 | 3 | 4 |
| LINC00960 | 1 | 1 | 2 |
| RBM12B-AS1 | 1 | 1 | 2 |
| UBL7-AS1 | 1 | 1 | 2 |
