# Supplementary material for: Competitive endogenous RNA network and pathway-based analysis of LncRNA single-nucleotide polymorphism in myasthenia gravis
Source: Sci Rep. 2021 Dec 14;11:23920. doi: 10.1038/s41598-021-03357-x (PMC8671434; doi:10.1038/s41598-021-03357-x)
Supplement: Supplementary file 5 — Supplementary Table S2. [file 41598_2021_3357_MOESM5_ESM.docx]

**Table S2 Enriched pathways of MG co-expressed mRNAs with lncRNAs.**

| Pathway Term | P-value |
| --- | --- |
| hsa05219: Bladder cancer | 5.12E-18 |
| hsa05161: Hepatitis B  hsa01522: Endocrine resistance  hsa01521: EGFR tyrosine kinase inhibitor resistance  hsa04010: MAPK signaling pathway  hsa05205: Proteoglycans in cancer  hsa05163: Human cytomegalovirus infection  hsa04933: AGE-RAGE signaling pathway in diabetic complications  hsa05220: Chronic myeloid leukemia  hsa05160: Hepatitis C  hsa05210: Colorectal cancer  hsa05218: Melanoma  hsa05224: Breast cancer  hsa05214: Glioma  hsa05225: Hepatocellular carcinoma  hsa05213: Endometrial cancer  hsa05216: Thyroid cancer | 4.09E-17 |
|  | 6.46E-17 |
|  | 2.25E-16 |
|  | 1.38E-14 |
|  | 4.30E-14 |
|  | 1.57E-13 |
|  | 1.95E-13 |
|  | 4.49E-13 |
|  | 1.08E-12 |
|  | 1.62E-12 |
|  | 1.25E-11 |
|  | 1.43E-11 |
|  | 1.83E-11 |
|  | 6.16E-11 |
|  | 8.69E-11 |
|  | 1.43E-10 |
| hsa05167: Kaposi sarcoma-associated herpesvirus infection  hsa05211: Renal cell carcinoma  hsa04917: Prolactin signaling pathway  hsa05226: Gastric cancer  hsa05223: Non-small cell lung cancer  hsa04218: Cellular senescence  hsa05212: Pancreatic cancer  hsa04071: Sphingolipid signaling pathway  hsa04722: Neurotrophin signaling pathway  hsa04068: FoxO signaling pathway  hsa04210: Apoptosis  hsa04370: VEGF signaling pathway  hsa04730: Long-term depression  hsa05215: Prostate cancer  hsa04550: Signaling pathways regulating pluripotency of stem cells  hsa04660: T cell receptor signaling pathway  hsa05221: Acute myeloid leukemia  hsa04015: Rap1 signaling pathway  hsa04664: Fc epsilon RI signaling pathway  hsa05170: Human immunodeficiency virus 1 infection  hsa04151: PI3K-Akt signaling pathway  hsa05230: Central carbon metabolism in cancer  hsa05166: Human T-cell leukemia virus 1 infection  hsa05206: MicroRNAs in cancer  hsa04926: Relaxin signaling pathway  hsa04012: ErbB signaling pathway  hsa04650: Natural killer cell mediated cytotoxicity  hsa05235: PD-L1 expression and PD-1 checkpoint pathway in cancer  hsa04140: Autophagy - animal  hsa04915: Estrogen signaling pathway  hsa05169: Epstein-Barr virus infection  hsa04150: mTOR signaling pathway  hsa04625: C-type lectin receptor signaling pathway  hsa04929: GnRH secretion  hsa04720: Long-term potentiation  hsa05022: Pathways of neurodegeneration - multiple diseases  hsa05145: Toxoplasmosis  hsa04919: Thyroid hormone signaling pathway  hsa05132: Salmonella infection  hsa04912: GnRH signaling pathway  hsa04932: Non-alcoholic fatty liver disease  hsa04914: Progesterone-mediated oocyte maturation  hsa05142: Chagas disease  hsa04064: NF-kappa B signaling pathway  hsa04630: JAK-STAT signaling pathway  hsa04014: Ras signaling pathway  hsa05164: Influenza A  hsa05165: Human papillomavirus infection  hsa04726: Serotonergic synapse  hsa05152: Tuberculosis  hsa04935: Growth hormone synthesis, secretion and action  hsa01524: Platinum drug resistance  hsa04062: Chemokine signaling pathway  hsa05202: Transcriptional misregulation in cancer  hsa05330: Allograft rejection  hsa04510: Focal adhesion  hsa04910: Insulin signaling pathway  hsa04662: B cell receptor signaling pathway  hsa05162: Measles  hsa04060: Cytokine-cytokine receptor interaction  hsa04810: Regulation of actin cytoskeleton  hsa04540: Gap junction  hsa04072: Phospholipase D signaling pathway  hsa04211: Longevity regulating pathway  hsa05323: Rheumatoid arthritis  hsa04657: IL-17 signaling pathway  hsa04217: Necroptosis  hsa05231: Choline metabolism in cancer  hsa05144: Malaria  hsa04916: Melanogenesis  hsa04620: Toll-like receptor signaling pathway  hsa04659: Th17 cell differentiation  hsa04066: HIF-1 signaling pathway  hsa04668: TNF signaling pathway  hsa04621: NOD-like receptor signaling pathway  hsa04725: Cholinergic synapse  hsa05034: Alcoholism  hsa04213: Longevity regulating pathway - multiple species  hsa05010: Alzheimer disease  hsa05130: Pathogenic Escherichia coli infection  hsa05203: Viral carcinogenesis  hsa04622: RIG-I-like receptor signaling pathway  hsa04115: p53 signaling pathway  hsa04371: Apelin signaling pathway  hsa05133: Pertussis  hsa05140: Leishmaniasis  hsa04921: Oxytocin signaling pathway  hsa05131: Shigellosis  hsa05222: Small cell lung cancer  hsa04350: TGF-beta signaling pathway  hsa05332: Graft-versus-host disease  hsa04940: Type I diabetes mellitus  hsa05020: Prion disease  hsa04360: Axon guidance  hsa04928: Parathyroid hormone synthesis, secretion and action  hsa04672: Intestinal immune network for IgA production  hsa05320: Autoimmune thyroid disease  hsa04380: Osteoclast differentiation  hsa05321: Inflammatory bowel disease  hsa05135: Yersinia infection  hsa05418: Fluid shear stress and atherosclerosis  hsa04137: Mitophagy - animal  hsa04934: Cushing syndrome  hsa05310: Asthma  hsa04215: Apoptosis - multiple species  hsa04640: Hematopoietic cell lineage  hsa04061: Viral protein interaction with cytokine and cytokine receptor  hsa05146: Amoebiasis  hsa04960: Aldosterone-regulated sodium reabsorption  hsa05143: African trypanosomiasis  hsa04110: Cell cycle  hsa04930: Type II diabetes mellitus  hsa04714: Thermogenesis  hsa04114: Oocyte meiosis  hsa05014: Amyotrophic lateral sclerosis  hsa04270: Vascular smooth muscle contraction  hsa04913: Ovarian steroidogenesis  hsa05322: Systemic lupus erythematosus  hsa04144: Endocytosis  hsa05134: Legionellosis  hsa04514: Cell adhesion molecules  hsa04261: Adrenergic signaling in cardiomyocytes  hsa05416: Viral myocarditis  hsa05120: Epithelial cell signaling in Helicobacter pylori infection  hsa04141: Protein processing in endoplasmic reticulum  hsa04520: Adherens junction | 2.76E-10 |
|  | 3.67E-10 |
|  | 4.13E-10 |
|  | 4.18E-10 |
|  | 5.20E-10 |
|  | 6.57E-10 |
|  | 8.10E-10 |
|  | 1.24E-09 |
|  | 1.24E-09 |
|  | 2.92E-09 |
|  | 4.07E-09 |
|  | 4.42E-09 |
|  | 4.98E-09 |
|  | 5.83E-09 |
|  | 6.35E-09 |
|  | 1.02E-08 |
|  | 1.10E-08 |
|  | 1.19E-08 |
|  | 1.22E-08 |
|  | 1.30E-08 |
|  | 1.40E-08 |
|  | 1.50E-08 |
|  | 1.78E-08 |
|  | 4.09E-08 |
|  | 5.58E-08 |
|  | 5.90E-08 |
|  | 6.30E-08 |
|  | 8.14E-08 |
|  | 8.94E-08 |
|  | 9.46E-08 |
|  | 1.29E-07 |
|  | 2.33E-07 |
|  | 2.40E-07 |
|  | 2.65E-07 |
|  | 3.49E-07 |
|  | 3.59E-07 |
|  | 3.99E-07 |
|  | 6.76E-07 |
|  | 7.63E-07 |
|  | 2.46E-06 |
|  | 2.88E-06 |
|  | 3.76E-06 |
|  | 4.22E-06 |
|  | 4.73E-06 |
|  | 4.81E-06 |
|  | 4.95E-06 |
|  | 6.88E-06 |
|  | 8.04E-06 |
|  | 8.48E-06 |
|  | 9.65E-06 |
|  | 1.03E-05 |
|  | 1.38E-05 |
|  | 1.47E-05 |
|  | 1.47E-05 |
|  | 1.97E-05 |
|  | 1.98E-05 |
|  | 2.31E-05 |
|  | 2.44E-05 |
|  | 2.51E-05 |
|  | 2.87E-05 |
|  | 3.35E-05 |
|  | 3.44E-05 |
|  | 3.58E-05 |
|  | 3.63E-05 |
|  | 4.49E-05 |
|  | 4.73E-05 |
|  | 5.36E-05 |
|  | 5.78E-05 |
|  | 5.91E-05 |
|  | 6.68E-05 |
|  | 7.68E-05 |
|  | 8.79E-05 |
|  | 9.60E-05 |
|  | 0.000109 |
|  | 0.00011 |
|  | 0.000114 |
|  | 0.000132 |
|  | 0.000138 |
|  | 0.00014 |
|  | 0.000176 |
|  | 0.000212 |
|  | 0.000221 |
|  | 0.00026 |
|  | 0.000282 |
|  | 0.000304 |
|  | 0.00032 |
|  | 0.000483 |
|  | 0.000581 |
|  | 0.00063 |
|  | 0.000684 |
|  | 0.000763 |
|  | 0.000817 |
|  | 0.001003 |
|  | 0.001006 |
|  | 0.001073 |
|  | 0.001199 |
|  | 0.001506 |
|  | 0.002155 |
|  | 0.00271 |
|  | 0.00276 |
|  | 0.002908 |
|  | 0.003082 |
|  | 0.004301 |
|  | 0.007842 |
|  | 0.008342 |
|  | 0.008803 |
|  | 0.009048 |
|  | 0.009551 |
|  | 0.011051 |
|  | 0.011051 |
|  | 0.016171 |
|  | 0.016761 |
|  | 0.017042 |
|  | 0.017959 |
|  | 0.019285 |
|  | 0.019466 |
|  | 0.020371 |
|  | 0.020641 |
|  | 0.02269 |
|  | 0.025091 |
|  | 0.02618 |
|  | 0.026637 |
|  | 0.027603 |
|  | 0.036664 |
|  | 0.037206 |
|  | 0.037626 |
