# Supplementary material for: Competitive endogenous RNA network and pathway-based analysis of LncRNA single-nucleotide polymorphism in myasthenia gravis
Source: Sci Rep. 2021 Dec 14;11:23920. doi: 10.1038/s41598-021-03357-x (PMC8671434; doi:10.1038/s41598-021-03357-x)
Supplement: Supplementary file 6 — Supplementary Table S3. [file 41598_2021_3357_MOESM6_ESM.docx]

**Table S3 Enriched GO annotation of MG co-expressed mRNAs with lncRNAs (TOP 100).**

| GO Term | P-value |
| --- | --- |
| GO:0043405:regulation of MAP kinase activity | 1.22E-12 |
| GO:0051403:stress-activated MAPK cascade | 5.40E-12 |
| GO:0097191:extrinsic apoptotic signaling pathway | 1.33E-11 |
| GO:0031098:stress-activated protein kinase signaling cascade | 1.53E-11 |
| GO:0051090:regulation of DNA-binding transcription factor activity | 2.22E-11 |
| GO:0032872:regulation of stress-activated MAPK cascade | 2.32E-11 |
| GO:0050673:epithelial cell proliferation | 2.34E-11 |
| GO:0070302:regulation of stress-activated protein kinase signaling cascade | 2.52E-11 |
| GO:0001819:positive regulation of cytokine production | 5.08E-11 |
| GO:0043406:positive regulation of MAP kinase activity | 5.35E-11 |
| GO:0070661:leukocyte proliferation | 2.20E-10 |
| GO:0070663:regulation of leukocyte proliferation | 3.76E-10 |
| GO:0010212:response to ionizing radiation | 3.88E-10 |
| GO:0071216:cellular response to biotic stimulus | 6.47E-10 |
| GO:0071902:positive regulation of protein serine/threonine kinase activity | 6.66E-10 |
| GO:0051402:neuron apoptotic process | 7.23E-10 |
| GO:0048872:homeostasis of number of cells | 9.33E-10 |
| GO:0031663:lipopolysaccharide-mediated signaling pathway | 1.49E-09 |
| GO:0050678:regulation of epithelial cell proliferation | 2.20E-09 |
| GO:0046651:lymphocyte proliferation | 2.26E-09 |
| GO:0032943:mononuclear cell proliferation | 2.41E-09 |
| GO:0022407:regulation of cell-cell adhesion | 4.07E-09 |
| GO:0002758:innate immune response-activating signal transduction | 5.01E-09 |
| GO:0018105:peptidyl-serine phosphorylation | 5.16E-09 |
| GO:0002687:positive regulation of leukocyte migration | 5.24E-09 |
| GO:0071222:cellular response to lipopolysaccharide | 5.41E-09 |
| GO:0050679:positive regulation of epithelial cell proliferation | 5.63E-09 |
| GO:0050670:regulation of lymphocyte proliferation | 6.07E-09 |
| GO:0032944:regulation of mononuclear cell proliferation | 6.30E-09 |
| GO:0071219:cellular response to molecule of bacterial origin | 7.05E-09 |
| GO:0048732:gland development | 8.26E-09 |
| GO:0070371:ERK1 and ERK2 cascade | 8.58E-09 |
| GO:0002218:activation of innate immune response | 9.06E-09 |
| GO:0002700:regulation of production of molecular mediator of immune response | 9.31E-09 |
| GO:0033135:regulation of peptidyl-serine phosphorylation | 9.31E-09 |
| GO:0018209:peptidyl-serine modification | 9.83E-09 |
| GO:0032103:positive regulation of response to external stimulus | 1.01E-08 |
| GO:0051054:positive regulation of DNA metabolic process | 1.25E-08 |
| GO:0002822:regulation of adaptive immune response based on somatic recombination of immune receptors built from immunoglobulin superfamily domains | 1.25E-08 |
| GO:0048754:branching morphogenesis of an epithelial tube | 1.58E-08 |
| GO:0008625:extrinsic apoptotic signaling pathway via death domain receptors | 1.66E-08 |
| GO:0002429:immune response-activating cell surface receptor signaling pathway | 1.87E-08 |
| GO:0070997:neuron death | 1.92E-08 |
| GO:0002819:regulation of adaptive immune response | 2.47E-08 |
| GO:2000027:regulation of animal organ morphogenesis | 2.80E-08 |
| GO:0002702:positive regulation of production of molecular mediator of immune response | 3.03E-08 |
| GO:0035690:cellular response to drug | 3.19E-08 |
| GO:0051091:positive regulation of DNA-binding transcription factor activity | 3.56E-08 |
| GO:0045089:positive regulation of innate immune response | 4.19E-08 |
| GO:0048545:response to steroid hormone | 4.59E-08 |
| GO:2001237:negative regulation of extrinsic apoptotic signaling pathway | 5.22E-08 |
| GO:0033138:positive regulation of peptidyl-serine phosphorylation | 5.53E-08 |
| GO:0061138:morphogenesis of a branching epithelium | 5.99E-08 |
| GO:0010332:response to gamma radiation | 8.19E-08 |
| GO:0001763:morphogenesis of a branching structure | 9.96E-08 |
| GO:0002685:regulation of leukocyte migration | 9.96E-08 |
| GO:1903037:regulation of leukocyte cell-cell adhesion | 1.15E-07 |
| GO:0022612:gland morphogenesis | 1.23E-07 |
| GO:0060249:anatomical structure homeostasis | 1.35E-07 |
| GO:0043523:regulation of neuron apoptotic process | 1.59E-07 |
| GO:0009314:response to radiation | 1.67E-07 |
| GO:0045088:regulation of innate immune response | 1.80E-07 |
| GO:0043281:regulation of cysteine-type endopeptidase activity involved in apoptotic process | 1.87E-07 |
| GO:0070374:positive regulation of ERK1 and ERK2 cascade | 1.87E-07 |
| GO:0002699:positive regulation of immune effector process | 1.93E-07 |
| GO:1904951:positive regulation of establishment of protein localization | 1.94E-07 |
| GO:0050671:positive regulation of lymphocyte proliferation | 1.98E-07 |
| GO:0002697:regulation of immune effector process | 2.01E-07 |
| GO:1903039:positive regulation of leukocyte cell-cell adhesion | 2.05E-07 |
| GO:0032946:positive regulation of mononuclear cell proliferation | 2.07E-07 |
| GO:0032496:response to lipopolysaccharide | 2.16E-07 |
| GO:0071214:cellular response to abiotic stimulus | 2.21E-07 |
| GO:0104004:cellular response to environmental stimulus | 2.21E-07 |
| GO:0050921:positive regulation of chemotaxis | 2.47E-07 |
| GO:0007159:leukocyte cell-cell adhesion | 2.54E-07 |
| GO:0002237:response to molecule of bacterial origin | 2.90E-07 |
| GO:0070665:positive regulation of leukocyte proliferation | 2.94E-07 |
| GO:2001234:negative regulation of apoptotic signaling pathway | 2.95E-07 |
| GO:0048871:multicellular organismal homeostasis | 3.26E-07 |
| GO:0051249:regulation of lymphocyte activation | 3.26E-07 |
| GO:0048873:homeostasis of number of cells within a tissue | 3.45E-07 |
| GO:2000116:regulation of cysteine-type endopeptidase activity | 3.83E-07 |
| GO:0002460:adaptive immune response based on somatic recombination of immune receptors built from immunoglobulin superfamily domains | 4.28E-07 |
| GO:0018108:peptidyl-tyrosine phosphorylation | 4.46E-07 |
| GO:0018212:peptidyl-tyrosine modification | 4.75E-07 |
| GO:0032675:regulation of interleukin-6 production | 4.98E-07 |
| GO:0110110:positive regulation of animal organ morphogenesis | 5.30E-07 |
| GO:2001236:regulation of extrinsic apoptotic signaling pathway | 5.58E-07 |
| GO:0022409:positive regulation of cell-cell adhesion | 5.93E-07 |
| GO:0050730:regulation of peptidyl-tyrosine phosphorylation | 6.08E-07 |
| GO:0002696:positive regulation of leukocyte activation | 6.30E-07 |
| GO:0002718:regulation of cytokine production involved in immune response | 6.36E-07 |
| GO:0032635:interleukin-6 production | 6.98E-07 |
| GO:0001776:leukocyte homeostasis | 7.16E-07 |
| GO:0002690:positive regulation of leukocyte chemotaxis | 7.58E-07 |
| GO:0002714:positive regulation of B cell mediated immunity | 8.23E-07 |
| GO:0002891:positive regulation of immunoglobulin mediated immune response | 8.23E-07 |
| GO:0050867:positive regulation of cell activation | 8.28E-07 |
| GO:0043491:protein kinase B signaling | 8.48E-07 |
| GO:0071674:mononuclear cell migration | 8.98E-07 |
