# Supplementary material for: Competitive endogenous RNA network and pathway-based analysis of LncRNA single-nucleotide polymorphism in myasthenia gravis
Source: Sci Rep. 2021 Dec 14;11:23920. doi: 10.1038/s41598-021-03357-x (PMC8671434; doi:10.1038/s41598-021-03357-x)
Supplement: Supplementary file 7 — Supplementary Table S4. [file 41598_2021_3357_MOESM7_ESM.docx]

**Table S4** **The details of lncRNAs regulation to MG risk pathways**

| LncRNA | Term | Genes | P-value |
| --- | --- | --- | --- |
| A2M-AS1 | hsa05219: Bladder cancer | BRAF, CDKN2A, FGFR3, HRAS, CXCL8, KRAS, MYC, NRAS, MAPK1, RAF1, VEGFA | 5.12E-18 |
| A2M-AS1 | hsa05205: Proteoglycans in cancer | FAS, BRAF, MAPK14, ESR1, HRAS, IGF1R, KRAS, MYC, NRAS, MAPK1, RAF1, TGFB1, TNF, VEGFA | 4.30E-14 |
| A2M-AS1 | hsa05163: Human cytomegalovirus infection | FAS, BAX, CDKN2A, MAPK14, HRAS, IL6R, CXCL8, KRAS, MYC, NRAS, MAPK1, RAF1, TNF, VEGFA | 1.57E-13 |
| A2M-AS1 | hsa05161: Hepatitis B | FAS, BAX, BCL2, BRAF, MAPK14, HRAS, CXCL8, KRAS, MYC, NRAS, MAPK1, RAF1, TGFB1, TNF, DDX58 | 4.09E-17 |
| A2M-AS1 | hsa05160: Hepatitis C | FAS, BAX, BRAF, HRAS, KRAS, LDLR, MYC, NRAS, MAPK1, RAF1, TNF, DDX58 | 1.08E-12 |
| A2M-AS1 | hsa04010: MAPK signaling pathway | FAS, BRAF, MAPK14, FGFR3, HRAS, IGF1R, KRAS, MAX, MAP3K11, MYC, NRAS, MAPK1, RAF1, TGFB1, TNF, VEGFA | 1.38E-14 |
| A2M-AS1 | hsa01522: Endocrine resistance | BAX, BCL2, BRAF, CDKN2A, MAPK14, ESR1, ESR2, HRAS, IGF1R, KRAS, NRAS, MAPK1, RAF1 | 6.46E-17 |
| A2M-AS1 | hsa01521: EGFR tyrosine kinase inhibitor resistance | BAX, BCL2, BRAF, FGFR3, HRAS, IGF1R, IL6R, KRAS, NRAS, MAPK1, RAF1, VEGFA | 2.25E-16 |
| AGAP2-AS1 | hsa05205: Proteoglycans in cancer | FAS, BRAF, MAPK14, ESR1, HRAS, IGF1R, KRAS, MYC, NRAS, MAPK1, RAF1, TGFB1, TNF, VEGFA | 4.30E-14 |
| AGAP2-AS1 | hsa05163: Human cytomegalovirus infection | FAS, BAX, CDKN2A, MAPK14, HRAS, IL6R, CXCL8, KRAS, MYC, NRAS, MAPK1, RAF1, TNF, VEGFA | 1.57E-13 |
| AGAP2-AS1 | hsa05161: Hepatitis B | FAS, BAX, BCL2, BRAF, MAPK14, HRAS, CXCL8, KRAS, MYC, NRAS, MAPK1, RAF1, TGFB1, TNF, DDX58 | 4.09E-17 |
| AGAP2-AS1 | hsa05160: Hepatitis C | FAS, BAX, BRAF, HRAS, KRAS, LDLR, MYC, NRAS, MAPK1, RAF1, TNF, DDX58 | 1.08E-12 |
| AGAP2-AS1 | hsa04010: MAPK signaling pathway | FAS, BRAF, MAPK14, FGFR3, HRAS, IGF1R, KRAS, MAX, MAP3K11, MYC, NRAS, MAPK1, RAF1, TGFB1, TNF, VEGFA | 1.38E-14 |
| AGAP2-AS1 | hsa01522: Endocrine resistance | BAX, BCL2, BRAF, CDKN2A, MAPK14, ESR1, ESR2, HRAS, IGF1R, KRAS, NRAS, MAPK1, RAF1 | 6.46E-17 |
| AGAP2-AS1 | hsa01521: EGFR tyrosine kinase inhibitor resistance | BAX, BCL2, BRAF, FGFR3, HRAS, IGF1R, IL6R, KRAS, NRAS, MAPK1, RAF1, VEGFA | 2.25E-16 |
| ARRDC3-AS1 | hsa05219: Bladder cancer | BRAF, CDKN2A, FGFR3, HRAS, CXCL8, KRAS, MYC, NRAS, MAPK1, RAF1, VEGFA | 5.12E-18 |
| ARRDC3-AS1 | hsa05205: Proteoglycans in cancer | FAS, BRAF, MAPK14, ESR1, HRAS, IGF1R, KRAS, MYC, NRAS, MAPK1, RAF1, TGFB1, TNF, VEGFA | 4.30E-14 |
| ARRDC3-AS1 | hsa05163: Human cytomegalovirus infection | FAS, BAX, CDKN2A, MAPK14, HRAS, IL6R, CXCL8, KRAS, MYC, NRAS, MAPK1, RAF1, TNF, VEGFA | 1.57E-13 |
| ARRDC3-AS1 | hsa05161: Hepatitis B | FAS, BAX, BCL2, BRAF, MAPK14, HRAS, CXCL8, KRAS, MYC, NRAS, MAPK1, RAF1, TGFB1, TNF, DDX58 | 4.09E-17 |
| ARRDC3-AS1 | hsa05160: Hepatitis C | FAS, BAX, BRAF, HRAS, KRAS, LDLR, MYC, NRAS, MAPK1, RAF1, TNF, DDX58 | 1.08E-12 |
| ARRDC3-AS1 | hsa04010: MAPK signaling pathway | FAS, BRAF, MAPK14, FGFR3, HRAS, IGF1R, KRAS, MAX, MAP3K11, MYC, NRAS, MAPK1, RAF1, TGFB1, TNF, VEGFA | 1.38E-14 |
| ARRDC3-AS1 | hsa01522: Endocrine resistance | BAX, BCL2, BRAF, CDKN2A, MAPK14, ESR1, ESR2, HRAS, IGF1R, KRAS, NRAS, MAPK1, RAF1 | 6.46E-17 |
| ARRDC3-AS1 | hsa01521: EGFR tyrosine kinase inhibitor resistance | BAX, BCL2, BRAF, FGFR3, HRAS, IGF1R, IL6R, KRAS, NRAS, MAPK1, RAF1, VEGFA | 2.25E-16 |
| ASB16-AS1 | hsa05161: Hepatitis B | FAS, BAX, BCL2, BRAF, MAPK14, HRAS, CXCL8, KRAS, MYC, NRAS, MAPK1, RAF1, TGFB1, TNF, DDX58 | 4.09E-17 |
| ASB16-AS1 | hsa05160: Hepatitis C | FAS, BAX, BRAF, HRAS, KRAS, LDLR, MYC, NRAS, MAPK1, RAF1, TNF, DDX58 | 1.08E-12 |
| ASH1L-AS1 | hsa05219: Bladder cancer | BRAF, CDKN2A, FGFR3, HRAS, CXCL8, KRAS, MYC, NRAS, MAPK1, RAF1, VEGFA | 5.12E-18 |
| ASH1L-AS1 | hsa05205: Proteoglycans in cancer | FAS, BRAF, MAPK14, ESR1, HRAS, IGF1R, KRAS, MYC, NRAS, MAPK1, RAF1, TGFB1, TNF, VEGFA | 4.30E-14 |
| ASH1L-AS1 | hsa05163: Human cytomegalovirus infection | FAS, BAX, CDKN2A, MAPK14, HRAS, IL6R, CXCL8, KRAS, MYC, NRAS, MAPK1, RAF1, TNF, VEGFA | 1.57E-13 |
| ASH1L-AS1 | hsa05161: Hepatitis B | FAS, BAX, BCL2, BRAF, MAPK14, HRAS, CXCL8, KRAS, MYC, NRAS, MAPK1, RAF1, TGFB1, TNF, DDX58 | 4.09E-17 |
| ASH1L-AS1 | hsa05160: Hepatitis C | FAS, BAX, BRAF, HRAS, KRAS, LDLR, MYC, NRAS, MAPK1, RAF1, TNF, DDX58 | 1.08E-12 |
| ASH1L-AS1 | hsa04010: MAPK signaling pathway | FAS, BRAF, MAPK14, FGFR3, HRAS, IGF1R, KRAS, MAX, MAP3K11, MYC, NRAS, MAPK1, RAF1, TGFB1, TNF, VEGFA | 1.38E-14 |
| ASH1L-AS1 | hsa01522: Endocrine resistance | BAX, BCL2, BRAF, CDKN2A, MAPK14, ESR1, ESR2, HRAS, IGF1R, KRAS, NRAS, MAPK1, RAF1 | 6.46E-17 |
| ASH1L-AS1 | hsa01521: EGFR tyrosine kinase inhibitor resistance | BAX, BCL2, BRAF, FGFR3, HRAS, IGF1R, IL6R, KRAS, NRAS, MAPK1, RAF1, VEGFA | 2.25E-16 |
| BCDIN3D-AS1 | hsa05161: Hepatitis B | FAS, BAX, BCL2, BRAF, MAPK14, HRAS, CXCL8, KRAS, MYC, NRAS, MAPK1, RAF1, TGFB1, TNF, DDX58 | 4.09E-17 |
| BCDIN3D-AS1 | hsa05160: Hepatitis C | FAS, BAX, BRAF, HRAS, KRAS, LDLR, MYC, NRAS, MAPK1, RAF1, TNF, DDX58 | 1.08E-12 |
| BDNF-AS | hsa05205: Proteoglycans in cancer | FAS, BRAF, MAPK14, ESR1, HRAS, IGF1R, KRAS, MYC, NRAS, MAPK1, RAF1, TGFB1, TNF, VEGFA | 4.30E-14 |
| BDNF-AS | hsa01522: Endocrine resistance | BAX, BCL2, BRAF, CDKN2A, MAPK14, ESR1, ESR2, HRAS, IGF1R, KRAS, NRAS, MAPK1, RAF1 | 6.46E-17 |
| C21orf62-AS1 | hsa05161: Hepatitis B | FAS, BAX, BCL2, BRAF, MAPK14, HRAS, CXCL8, KRAS, MYC, NRAS, MAPK1, RAF1, TGFB1, TNF, DDX58 | 4.09E-17 |
| C21orf62-AS1 | hsa01522: Endocrine resistance | BAX, BCL2, BRAF, CDKN2A, MAPK14, ESR1, ESR2, HRAS, IGF1R, KRAS, NRAS, MAPK1, RAF1 | 6.46E-17 |
| C21orf62-AS1 | hsa01521: EGFR tyrosine kinase inhibitor resistance | BAX, BCL2, BRAF, FGFR3, HRAS, IGF1R, IL6R, KRAS, NRAS, MAPK1, RAF1, VEGFA | 2.25E-16 |
| CDKN2B-AS1 | hsa05161: Hepatitis B | FAS, BAX, BCL2, BRAF, MAPK14, HRAS, CXCL8, KRAS, MYC, NRAS, MAPK1, RAF1, TGFB1, TNF, DDX58 | 4.09E-17 |
| CDKN2B-AS1 | hsa01522: Endocrine resistance | BAX, BCL2, BRAF, CDKN2A, MAPK14, ESR1, ESR2, HRAS, IGF1R, KRAS, NRAS, MAPK1, RAF1 | 6.46E-17 |
| CDKN2B-AS1 | hsa01521: EGFR tyrosine kinase inhibitor resistance | BAX, BCL2, BRAF, FGFR3, HRAS, IGF1R, IL6R, KRAS, NRAS, MAPK1, RAF1, VEGFA | 2.25E-16 |
| CEBPA-AS1 | hsa05205: Proteoglycans in cancer | FAS, BRAF, MAPK14, ESR1, HRAS, IGF1R, KRAS, MYC, NRAS, MAPK1, RAF1, TGFB1, TNF, VEGFA | 4.30E-14 |
| CEBPA-AS1 | hsa05163: Human cytomegalovirus infection | FAS, BAX, CDKN2A, MAPK14, HRAS, IL6R, CXCL8, KRAS, MYC, NRAS, MAPK1, RAF1, TNF, VEGFA | 1.57E-13 |
| CEBPA-AS1 | hsa05161: Hepatitis B | FAS, BAX, BCL2, BRAF, MAPK14, HRAS, CXCL8, KRAS, MYC, NRAS, MAPK1, RAF1, TGFB1, TNF, DDX58 | 4.09E-17 |
| CEBPA-AS1 | hsa04010: MAPK signaling pathway | FAS, BRAF, MAPK14, FGFR3, HRAS, IGF1R, KRAS, MAX, MAP3K11, MYC, NRAS, MAPK1, RAF1, TGFB1, TNF, VEGFA | 1.38E-14 |
| CEBPA-AS1 | hsa01522: Endocrine resistance | BAX, BCL2, BRAF, CDKN2A, MAPK14, ESR1, ESR2, HRAS, IGF1R, KRAS, NRAS, MAPK1, RAF1 | 6.46E-17 |
| CECR7 | hsa05219: Bladder cancer | BRAF, CDKN2A, FGFR3, HRAS, CXCL8, KRAS, MYC, NRAS, MAPK1, RAF1, VEGFA | 5.12E-18 |
| CECR7 | hsa05205: Proteoglycans in cancer | FAS, BRAF, MAPK14, ESR1, HRAS, IGF1R, KRAS, MYC, NRAS, MAPK1, RAF1, TGFB1, TNF, VEGFA | 4.30E-14 |
| CECR7 | hsa05163: Human cytomegalovirus infection | FAS, BAX, CDKN2A, MAPK14, HRAS, IL6R, CXCL8, KRAS, MYC, NRAS, MAPK1, RAF1, TNF, VEGFA | 1.57E-13 |
| CECR7 | hsa05161: Hepatitis B | FAS, BAX, BCL2, BRAF, MAPK14, HRAS, CXCL8, KRAS, MYC, NRAS, MAPK1, RAF1, TGFB1, TNF, DDX58 | 4.09E-17 |
| CECR7 | hsa05160: Hepatitis C | FAS, BAX, BRAF, HRAS, KRAS, LDLR, MYC, NRAS, MAPK1, RAF1, TNF, DDX58 | 1.08E-12 |
| CECR7 | hsa04010: MAPK signaling pathway | FAS, BRAF, MAPK14, FGFR3, HRAS, IGF1R, KRAS, MAX, MAP3K11, MYC, NRAS, MAPK1, RAF1, TGFB1, TNF, VEGFA | 1.38E-14 |
| CTBP1-AS2 | hsa05161: Hepatitis B | FAS, BAX, BCL2, BRAF, MAPK14, HRAS, CXCL8, KRAS, MYC, NRAS, MAPK1, RAF1, TGFB1, TNF, DDX58 | 4.09E-17 |
| CTBP1-AS2 | hsa05160: Hepatitis C | FAS, BAX, BRAF, HRAS, KRAS, LDLR, MYC, NRAS, MAPK1, RAF1, TNF, DDX58 | 1.08E-12 |
| CTBP1-AS2 | hsa01522: Endocrine resistance | BAX, BCL2, BRAF, CDKN2A, MAPK14, ESR1, ESR2, HRAS, IGF1R, KRAS, NRAS, MAPK1, RAF1 | 6.46E-17 |
| CTBP1-AS2 | hsa01521: EGFR tyrosine kinase inhibitor resistance | BAX, BCL2, BRAF, FGFR3, HRAS, IGF1R, IL6R, KRAS, NRAS, MAPK1, RAF1, VEGFA | 2.25E-16 |
| CYP1B1-AS1 | hsa05205: Proteoglycans in cancer | FAS, BRAF, MAPK14, ESR1, HRAS, IGF1R, KRAS, MYC, NRAS, MAPK1, RAF1, TGFB1, TNF, VEGFA | 4.30E-14 |
| CYP1B1-AS1 | hsa05163: Human cytomegalovirus infection | FAS, BAX, CDKN2A, MAPK14, HRAS, IL6R, CXCL8, KRAS, MYC, NRAS, MAPK1, RAF1, TNF, VEGFA | 1.57E-13 |
| CYP1B1-AS1 | hsa05161: Hepatitis B | FAS, BAX, BCL2, BRAF, MAPK14, HRAS, CXCL8, KRAS, MYC, NRAS, MAPK1, RAF1, TGFB1, TNF, DDX58 | 4.09E-17 |
| CYP1B1-AS1 | hsa04010: MAPK signaling pathway | FAS, BRAF, MAPK14, FGFR3, HRAS, IGF1R, KRAS, MAX, MAP3K11, MYC, NRAS, MAPK1, RAF1, TGFB1, TNF, VEGFA | 1.38E-14 |
| CYP1B1-AS1 | hsa01522: Endocrine resistance | BAX, BCL2, BRAF, CDKN2A, MAPK14, ESR1, ESR2, HRAS, IGF1R, KRAS, NRAS, MAPK1, RAF1 | 6.46E-17 |
| CYP1B1-AS1 | hsa01521: EGFR tyrosine kinase inhibitor resistance | BAX, BCL2, BRAF, FGFR3, HRAS, IGF1R, IL6R, KRAS, NRAS, MAPK1, RAF1, VEGFA | 2.25E-16 |
| CYP4F35P | hsa05219: Bladder cancer | BRAF, CDKN2A, FGFR3, HRAS, CXCL8, KRAS, MYC, NRAS, MAPK1, RAF1, VEGFA | 5.12E-18 |
| CYP4F35P | hsa05205: Proteoglycans in cancer | FAS, BRAF, MAPK14, ESR1, HRAS, IGF1R, KRAS, MYC, NRAS, MAPK1, RAF1, TGFB1, TNF, VEGFA | 4.30E-14 |
| CYP4F35P | hsa05163: Human cytomegalovirus infection | FAS, BAX, CDKN2A, MAPK14, HRAS, IL6R, CXCL8, KRAS, MYC, NRAS, MAPK1, RAF1, TNF, VEGFA | 1.57E-13 |
| CYP4F35P | hsa05161: Hepatitis B | FAS, BAX, BCL2, BRAF, MAPK14, HRAS, CXCL8, KRAS, MYC, NRAS, MAPK1, RAF1, TGFB1, TNF, DDX58 | 4.09E-17 |
| CYP4F35P | hsa05160: Hepatitis C | FAS, BAX, BRAF, HRAS, KRAS, LDLR, MYC, NRAS, MAPK1, RAF1, TNF, DDX58 | 1.08E-12 |
| CYP4F35P | hsa04010: MAPK signaling pathway | FAS, BRAF, MAPK14, FGFR3, HRAS, IGF1R, KRAS, MAX, MAP3K11, MYC, NRAS, MAPK1, RAF1, TGFB1, TNF, VEGFA | 1.38E-14 |
| CYP4F35P | hsa01522: Endocrine resistance | BAX, BCL2, BRAF, CDKN2A, MAPK14, ESR1, ESR2, HRAS, IGF1R, KRAS, NRAS, MAPK1, RAF1 | 6.46E-17 |
| CYP4F35P | hsa01521: EGFR tyrosine kinase inhibitor resistance | BAX, BCL2, BRAF, FGFR3, HRAS, IGF1R, IL6R, KRAS, NRAS, MAPK1, RAF1, VEGFA | 2.25E-16 |
| DANCR | hsa05219: Bladder cancer | BRAF, CDKN2A, FGFR3, HRAS, CXCL8, KRAS, MYC, NRAS, MAPK1, RAF1, VEGFA | 5.12E-18 |
| DANCR | hsa05205: Proteoglycans in cancer | FAS, BRAF, MAPK14, ESR1, HRAS, IGF1R, KRAS, MYC, NRAS, MAPK1, RAF1, TGFB1, TNF, VEGFA | 4.30E-14 |
| DANCR | hsa05163: Human cytomegalovirus infection | FAS, BAX, CDKN2A, MAPK14, HRAS, IL6R, CXCL8, KRAS, MYC, NRAS, MAPK1, RAF1, TNF, VEGFA | 1.57E-13 |
| DANCR | hsa05161: Hepatitis B | FAS, BAX, BCL2, BRAF, MAPK14, HRAS, CXCL8, KRAS, MYC, NRAS, MAPK1, RAF1, TGFB1, TNF, DDX58 | 4.09E-17 |
| DANCR | hsa05160: Hepatitis C | FAS, BAX, BRAF, HRAS, KRAS, LDLR, MYC, NRAS, MAPK1, RAF1, TNF, DDX58 | 1.08E-12 |
| DANCR | hsa04010: MAPK signaling pathway | FAS, BRAF, MAPK14, FGFR3, HRAS, IGF1R, KRAS, MAX, MAP3K11, MYC, NRAS, MAPK1, RAF1, TGFB1, TNF, VEGFA | 1.38E-14 |
| DANCR | hsa01522: Endocrine resistance | BAX, BCL2, BRAF, CDKN2A, MAPK14, ESR1, ESR2, HRAS, IGF1R, KRAS, NRAS, MAPK1, RAF1 | 6.46E-17 |
| DANCR | hsa01521: EGFR tyrosine kinase inhibitor resistance | BAX, BCL2, BRAF, FGFR3, HRAS, IGF1R, IL6R, KRAS, NRAS, MAPK1, RAF1, VEGFA | 2.25E-16 |
| DICER1-AS1 | hsa05219: Bladder cancer | BRAF, CDKN2A, FGFR3, HRAS, CXCL8, KRAS, MYC, NRAS, MAPK1, RAF1, VEGFA | 5.12E-18 |
| DICER1-AS1 | hsa05205: Proteoglycans in cancer | FAS, BRAF, MAPK14, ESR1, HRAS, IGF1R, KRAS, MYC, NRAS, MAPK1, RAF1, TGFB1, TNF, VEGFA | 4.30E-14 |
| DICER1-AS1 | hsa05163: Human cytomegalovirus infection | FAS, BAX, CDKN2A, MAPK14, HRAS, IL6R, CXCL8, KRAS, MYC, NRAS, MAPK1, RAF1, TNF, VEGFA | 1.57E-13 |
| DICER1-AS1 | hsa05161: Hepatitis B | FAS, BAX, BCL2, BRAF, MAPK14, HRAS, CXCL8, KRAS, MYC, NRAS, MAPK1, RAF1, TGFB1, TNF, DDX58 | 4.09E-17 |
| DICER1-AS1 | hsa05160: Hepatitis C | FAS, BAX, BRAF, HRAS, KRAS, LDLR, MYC, NRAS, MAPK1, RAF1, TNF, DDX58 | 1.08E-12 |
| DICER1-AS1 | hsa04010: MAPK signaling pathway | FAS, BRAF, MAPK14, FGFR3, HRAS, IGF1R, KRAS, MAX, MAP3K11, MYC, NRAS, MAPK1, RAF1, TGFB1, TNF, VEGFA | 1.38E-14 |
| DICER1-AS1 | hsa01522: Endocrine resistance | BAX, BCL2, BRAF, CDKN2A, MAPK14, ESR1, ESR2, HRAS, IGF1R, KRAS, NRAS, MAPK1, RAF1 | 6.46E-17 |
| DICER1-AS1 | hsa01521: EGFR tyrosine kinase inhibitor resistance | BAX, BCL2, BRAF, FGFR3, HRAS, IGF1R, IL6R, KRAS, NRAS, MAPK1, RAF1, VEGFA | 2.25E-16 |
| DKFZP434I0714 | hsa05219: Bladder cancer | BRAF, CDKN2A, FGFR3, HRAS, CXCL8, KRAS, MYC, NRAS, MAPK1, RAF1, VEGFA | 5.12E-18 |
| DKFZP434I0714 | hsa05205: Proteoglycans in cancer | FAS, BRAF, MAPK14, ESR1, HRAS, IGF1R, KRAS, MYC, NRAS, MAPK1, RAF1, TGFB1, TNF, VEGFA | 4.30E-14 |
| DKFZP434I0714 | hsa05163: Human cytomegalovirus infection | FAS, BAX, CDKN2A, MAPK14, HRAS, IL6R, CXCL8, KRAS, MYC, NRAS, MAPK1, RAF1, TNF, VEGFA | 1.57E-13 |
| DKFZP434I0714 | hsa05161: Hepatitis B | FAS, BAX, BCL2, BRAF, MAPK14, HRAS, CXCL8, KRAS, MYC, NRAS, MAPK1, RAF1, TGFB1, TNF, DDX58 | 4.09E-17 |
| DKFZP434I0714 | hsa05160: Hepatitis C | FAS, BAX, BRAF, HRAS, KRAS, LDLR, MYC, NRAS, MAPK1, RAF1, TNF, DDX58 | 1.08E-12 |
| DKFZP434I0714 | hsa04010: MAPK signaling pathway | FAS, BRAF, MAPK14, FGFR3, HRAS, IGF1R, KRAS, MAX, MAP3K11, MYC, NRAS, MAPK1, RAF1, TGFB1, TNF, VEGFA | 1.38E-14 |
| DKFZP434I0714 | hsa01522: Endocrine resistance | BAX, BCL2, BRAF, CDKN2A, MAPK14, ESR1, ESR2, HRAS, IGF1R, KRAS, NRAS, MAPK1, RAF1 | 6.46E-17 |
| DKFZP434I0714 | hsa01521: EGFR tyrosine kinase inhibitor resistance | BAX, BCL2, BRAF, FGFR3, HRAS, IGF1R, IL6R, KRAS, NRAS, MAPK1, RAF1, VEGFA | 2.25E-16 |
| DLEU2 | hsa05219: Bladder cancer | BRAF, CDKN2A, FGFR3, HRAS, CXCL8, KRAS, MYC, NRAS, MAPK1, RAF1, VEGFA | 5.12E-18 |
| DLEU2 | hsa05205: Proteoglycans in cancer | FAS, BRAF, MAPK14, ESR1, HRAS, IGF1R, KRAS, MYC, NRAS, MAPK1, RAF1, TGFB1, TNF, VEGFA | 4.30E-14 |
| DLEU2 | hsa05163: Human cytomegalovirus infection | FAS, BAX, CDKN2A, MAPK14, HRAS, IL6R, CXCL8, KRAS, MYC, NRAS, MAPK1, RAF1, TNF, VEGFA | 1.57E-13 |
| DLEU2 | hsa05161: Hepatitis B | FAS, BAX, BCL2, BRAF, MAPK14, HRAS, CXCL8, KRAS, MYC, NRAS, MAPK1, RAF1, TGFB1, TNF, DDX58 | 4.09E-17 |
| DLEU2 | hsa05160: Hepatitis C | FAS, BAX, BRAF, HRAS, KRAS, LDLR, MYC, NRAS, MAPK1, RAF1, TNF, DDX58 | 1.08E-12 |
| DLEU2 | hsa04010: MAPK signaling pathway | FAS, BRAF, MAPK14, FGFR3, HRAS, IGF1R, KRAS, MAX, MAP3K11, MYC, NRAS, MAPK1, RAF1, TGFB1, TNF, VEGFA | 1.38E-14 |
| DLEU2 | hsa01522: Endocrine resistance | BAX, BCL2, BRAF, CDKN2A, MAPK14, ESR1, ESR2, HRAS, IGF1R, KRAS, NRAS, MAPK1, RAF1 | 6.46E-17 |
| DLEU2 | hsa01521: EGFR tyrosine kinase inhibitor resistance | BAX, BCL2, BRAF, FGFR3, HRAS, IGF1R, IL6R, KRAS, NRAS, MAPK1, RAF1, VEGFA | 2.25E-16 |
| DLGAP1-AS2 | hsa05219: Bladder cancer | BRAF, CDKN2A, FGFR3, HRAS, CXCL8, KRAS, MYC, NRAS, MAPK1, RAF1, VEGFA | 5.12E-18 |
| DLGAP1-AS2 | hsa05205: Proteoglycans in cancer | FAS, BRAF, MAPK14, ESR1, HRAS, IGF1R, KRAS, MYC, NRAS, MAPK1, RAF1, TGFB1, TNF, VEGFA | 4.30E-14 |
| DLGAP1-AS2 | hsa05163: Human cytomegalovirus infection | FAS, BAX, CDKN2A, MAPK14, HRAS, IL6R, CXCL8, KRAS, MYC, NRAS, MAPK1, RAF1, TNF, VEGFA | 1.57E-13 |
| DLGAP1-AS2 | hsa05161: Hepatitis B | FAS, BAX, BCL2, BRAF, MAPK14, HRAS, CXCL8, KRAS, MYC, NRAS, MAPK1, RAF1, TGFB1, TNF, DDX58 | 4.09E-17 |
| DLGAP1-AS2 | hsa05160: Hepatitis C | FAS, BAX, BRAF, HRAS, KRAS, LDLR, MYC, NRAS, MAPK1, RAF1, TNF, DDX58 | 1.08E-12 |
| DLGAP1-AS2 | hsa04010: MAPK signaling pathway | FAS, BRAF, MAPK14, FGFR3, HRAS, IGF1R, KRAS, MAX, MAP3K11, MYC, NRAS, MAPK1, RAF1, TGFB1, TNF, VEGFA | 1.38E-14 |
| DLGAP1-AS2 | hsa01522: Endocrine resistance | BAX, BCL2, BRAF, CDKN2A, MAPK14, ESR1, ESR2, HRAS, IGF1R, KRAS, NRAS, MAPK1, RAF1 | 6.46E-17 |
| DLGAP1-AS2 | hsa01521: EGFR tyrosine kinase inhibitor resistance | BAX, BCL2, BRAF, FGFR3, HRAS, IGF1R, IL6R, KRAS, NRAS, MAPK1, RAF1, VEGFA | 2.25E-16 |
| EPB41L4A-AS1 | hsa05219: Bladder cancer | BRAF, CDKN2A, FGFR3, HRAS, CXCL8, KRAS, MYC, NRAS, MAPK1, RAF1, VEGFA | 5.12E-18 |
| EPB41L4A-AS1 | hsa05205: Proteoglycans in cancer | FAS, BRAF, MAPK14, ESR1, HRAS, IGF1R, KRAS, MYC, NRAS, MAPK1, RAF1, TGFB1, TNF, VEGFA | 4.30E-14 |
| EPB41L4A-AS1 | hsa05163: Human cytomegalovirus infection | FAS, BAX, CDKN2A, MAPK14, HRAS, IL6R, CXCL8, KRAS, MYC, NRAS, MAPK1, RAF1, TNF, VEGFA | 1.57E-13 |
| EPB41L4A-AS1 | hsa05161: Hepatitis B | FAS, BAX, BCL2, BRAF, MAPK14, HRAS, CXCL8, KRAS, MYC, NRAS, MAPK1, RAF1, TGFB1, TNF, DDX58 | 4.09E-17 |
| EPB41L4A-AS1 | hsa05160: Hepatitis C | FAS, BAX, BRAF, HRAS, KRAS, LDLR, MYC, NRAS, MAPK1, RAF1, TNF, DDX58 | 1.08E-12 |
| EPB41L4A-AS1 | hsa04010: MAPK signaling pathway | FAS, BRAF, MAPK14, FGFR3, HRAS, IGF1R, KRAS, MAX, MAP3K11, MYC, NRAS, MAPK1, RAF1, TGFB1, TNF, VEGFA | 1.38E-14 |
| EPB41L4A-AS1 | hsa01522: Endocrine resistance | BAX, BCL2, BRAF, CDKN2A, MAPK14, ESR1, ESR2, HRAS, IGF1R, KRAS, NRAS, MAPK1, RAF1 | 6.46E-17 |
| EPB41L4A-AS1 | hsa01521: EGFR tyrosine kinase inhibitor resistance | BAX, BCL2, BRAF, FGFR3, HRAS, IGF1R, IL6R, KRAS, NRAS, MAPK1, RAF1, VEGFA | 2.25E-16 |
| FAM13A-AS1 | hsa05219: Bladder cancer | BRAF, CDKN2A, FGFR3, HRAS, CXCL8, KRAS, MYC, NRAS, MAPK1, RAF1, VEGFA | 5.12E-18 |
| FAM13A-AS1 | hsa05205: Proteoglycans in cancer | FAS, BRAF, MAPK14, ESR1, HRAS, IGF1R, KRAS, MYC, NRAS, MAPK1, RAF1, TGFB1, TNF, VEGFA | 4.30E-14 |
| FAM13A-AS1 | hsa05163: Human cytomegalovirus infection | FAS, BAX, CDKN2A, MAPK14, HRAS, IL6R, CXCL8, KRAS, MYC, NRAS, MAPK1, RAF1, TNF, VEGFA | 1.57E-13 |
| FAM13A-AS1 | hsa05161: Hepatitis B | FAS, BAX, BCL2, BRAF, MAPK14, HRAS, CXCL8, KRAS, MYC, NRAS, MAPK1, RAF1, TGFB1, TNF, DDX58 | 4.09E-17 |
| FAM13A-AS1 | hsa05160: Hepatitis C | FAS, BAX, BRAF, HRAS, KRAS, LDLR, MYC, NRAS, MAPK1, RAF1, TNF, DDX58 | 1.08E-12 |
| FAM13A-AS1 | hsa04010: MAPK signaling pathway | FAS, BRAF, MAPK14, FGFR3, HRAS, IGF1R, KRAS, MAX, MAP3K11, MYC, NRAS, MAPK1, RAF1, TGFB1, TNF, VEGFA | 1.38E-14 |
| FAM13A-AS1 | hsa01522: Endocrine resistance | BAX, BCL2, BRAF, CDKN2A, MAPK14, ESR1, ESR2, HRAS, IGF1R, KRAS, NRAS, MAPK1, RAF1 | 6.46E-17 |
| FAM13A-AS1 | hsa01521: EGFR tyrosine kinase inhibitor resistance | BAX, BCL2, BRAF, FGFR3, HRAS, IGF1R, IL6R, KRAS, NRAS, MAPK1, RAF1, VEGFA | 2.25E-16 |
| FAM225A | hsa05205: Proteoglycans in cancer | FAS, BRAF, MAPK14, ESR1, HRAS, IGF1R, KRAS, MYC, NRAS, MAPK1, RAF1, TGFB1, TNF, VEGFA | 4.30E-14 |
| FAM225A | hsa05163: Human cytomegalovirus infection | FAS, BAX, CDKN2A, MAPK14, HRAS, IL6R, CXCL8, KRAS, MYC, NRAS, MAPK1, RAF1, TNF, VEGFA | 1.57E-13 |
| FAM225A | hsa05161: Hepatitis B | FAS, BAX, BCL2, BRAF, MAPK14, HRAS, CXCL8, KRAS, MYC, NRAS, MAPK1, RAF1, TGFB1, TNF, DDX58 | 4.09E-17 |
| FAM225A | hsa05160: Hepatitis C | FAS, BAX, BRAF, HRAS, KRAS, LDLR, MYC, NRAS, MAPK1, RAF1, TNF, DDX58 | 1.08E-12 |
| FAM225A | hsa04010: MAPK signaling pathway | FAS, BRAF, MAPK14, FGFR3, HRAS, IGF1R, KRAS, MAX, MAP3K11, MYC, NRAS, MAPK1, RAF1, TGFB1, TNF, VEGFA | 1.38E-14 |
| FAM225A | hsa01521: EGFR tyrosine kinase inhibitor resistance | BAX, BCL2, BRAF, FGFR3, HRAS, IGF1R, IL6R, KRAS, NRAS, MAPK1, RAF1, VEGFA | 2.25E-16 |
| FAM225B | hsa05163: Human cytomegalovirus infection | FAS, BAX, CDKN2A, MAPK14, HRAS, IL6R, CXCL8, KRAS, MYC, NRAS, MAPK1, RAF1, TNF, VEGFA | 1.57E-13 |
| FAM225B | hsa01521: EGFR tyrosine kinase inhibitor resistance | BAX, BCL2, BRAF, FGFR3, HRAS, IGF1R, IL6R, KRAS, NRAS, MAPK1, RAF1, VEGFA | 2.25E-16 |
| FAM66C | hsa05161: Hepatitis B | FAS, BAX, BCL2, BRAF, MAPK14, HRAS, CXCL8, KRAS, MYC, NRAS, MAPK1, RAF1, TGFB1, TNF, DDX58 | 4.09E-17 |
| FAM66C | hsa01522: Endocrine resistance | BAX, BCL2, BRAF, CDKN2A, MAPK14, ESR1, ESR2, HRAS, IGF1R, KRAS, NRAS, MAPK1, RAF1 | 6.46E-17 |
| FAM66C | hsa01521: EGFR tyrosine kinase inhibitor resistance | BAX, BCL2, BRAF, FGFR3, HRAS, IGF1R, IL6R, KRAS, NRAS, MAPK1, RAF1, VEGFA | 2.25E-16 |
| FBXL19-AS1 | hsa05219: Bladder cancer | BRAF, CDKN2A, FGFR3, HRAS, CXCL8, KRAS, MYC, NRAS, MAPK1, RAF1, VEGFA | 5.12E-18 |
| FBXL19-AS1 | hsa05205: Proteoglycans in cancer | FAS, BRAF, MAPK14, ESR1, HRAS, IGF1R, KRAS, MYC, NRAS, MAPK1, RAF1, TGFB1, TNF, VEGFA | 4.30E-14 |
| FBXL19-AS1 | hsa05163: Human cytomegalovirus infection | FAS, BAX, CDKN2A, MAPK14, HRAS, IL6R, CXCL8, KRAS, MYC, NRAS, MAPK1, RAF1, TNF, VEGFA | 1.57E-13 |
| FBXL19-AS1 | hsa05161: Hepatitis B | FAS, BAX, BCL2, BRAF, MAPK14, HRAS, CXCL8, KRAS, MYC, NRAS, MAPK1, RAF1, TGFB1, TNF, DDX58 | 4.09E-17 |
| FBXL19-AS1 | hsa05160: Hepatitis C | FAS, BAX, BRAF, HRAS, KRAS, LDLR, MYC, NRAS, MAPK1, RAF1, TNF, DDX58 | 1.08E-12 |
| FBXL19-AS1 | hsa04010: MAPK signaling pathway | FAS, BRAF, MAPK14, FGFR3, HRAS, IGF1R, KRAS, MAX, MAP3K11, MYC, NRAS, MAPK1, RAF1, TGFB1, TNF, VEGFA | 1.38E-14 |
| FBXL19-AS1 | hsa01522: Endocrine resistance | BAX, BCL2, BRAF, CDKN2A, MAPK14, ESR1, ESR2, HRAS, IGF1R, KRAS, NRAS, MAPK1, RAF1 | 6.46E-17 |
| FBXL19-AS1 | hsa01521: EGFR tyrosine kinase inhibitor resistance | BAX, BCL2, BRAF, FGFR3, HRAS, IGF1R, IL6R, KRAS, NRAS, MAPK1, RAF1, VEGFA | 2.25E-16 |
| FGF13-AS1 | hsa05205: Proteoglycans in cancer | FAS, BRAF, MAPK14, ESR1, HRAS, IGF1R, KRAS, MYC, NRAS, MAPK1, RAF1, TGFB1, TNF, VEGFA | 4.30E-14 |
| FGF13-AS1 | hsa05163: Human cytomegalovirus infection | FAS, BAX, CDKN2A, MAPK14, HRAS, IL6R, CXCL8, KRAS, MYC, NRAS, MAPK1, RAF1, TNF, VEGFA | 1.57E-13 |
| FGF13-AS1 | hsa05161: Hepatitis B | FAS, BAX, BCL2, BRAF, MAPK14, HRAS, CXCL8, KRAS, MYC, NRAS, MAPK1, RAF1, TGFB1, TNF, DDX58 | 4.09E-17 |
| FGF13-AS1 | hsa04010: MAPK signaling pathway | FAS, BRAF, MAPK14, FGFR3, HRAS, IGF1R, KRAS, MAX, MAP3K11, MYC, NRAS, MAPK1, RAF1, TGFB1, TNF, VEGFA | 1.38E-14 |
| FGF13-AS1 | hsa01522: Endocrine resistance | BAX, BCL2, BRAF, CDKN2A, MAPK14, ESR1, ESR2, HRAS, IGF1R, KRAS, NRAS, MAPK1, RAF1 | 6.46E-17 |
| FLJ22447 | hsa05219: Bladder cancer | BRAF, CDKN2A, FGFR3, HRAS, CXCL8, KRAS, MYC, NRAS, MAPK1, RAF1, VEGFA | 5.12E-18 |
| FLJ22447 | hsa05205: Proteoglycans in cancer | FAS, BRAF, MAPK14, ESR1, HRAS, IGF1R, KRAS, MYC, NRAS, MAPK1, RAF1, TGFB1, TNF, VEGFA | 4.30E-14 |
| FLJ22447 | hsa05163: Human cytomegalovirus infection | FAS, BAX, CDKN2A, MAPK14, HRAS, IL6R, CXCL8, KRAS, MYC, NRAS, MAPK1, RAF1, TNF, VEGFA | 1.57E-13 |
| FLJ22447 | hsa05161: Hepatitis B | FAS, BAX, BCL2, BRAF, MAPK14, HRAS, CXCL8, KRAS, MYC, NRAS, MAPK1, RAF1, TGFB1, TNF, DDX58 | 4.09E-17 |
| FLJ22447 | hsa05160: Hepatitis C | FAS, BAX, BRAF, HRAS, KRAS, LDLR, MYC, NRAS, MAPK1, RAF1, TNF, DDX58 | 1.08E-12 |
| FLJ22447 | hsa04010: MAPK signaling pathway | FAS, BRAF, MAPK14, FGFR3, HRAS, IGF1R, KRAS, MAX, MAP3K11, MYC, NRAS, MAPK1, RAF1, TGFB1, TNF, VEGFA | 1.38E-14 |
| FLJ22447 | hsa01522: Endocrine resistance | BAX, BCL2, BRAF, CDKN2A, MAPK14, ESR1, ESR2, HRAS, IGF1R, KRAS, NRAS, MAPK1, RAF1 | 6.46E-17 |
| FLJ22447 | hsa01521: EGFR tyrosine kinase inhibitor resistance | BAX, BCL2, BRAF, FGFR3, HRAS, IGF1R, IL6R, KRAS, NRAS, MAPK1, RAF1, VEGFA | 2.25E-16 |
| GHRLOS | hsa05219: Bladder cancer | BRAF, CDKN2A, FGFR3, HRAS, CXCL8, KRAS, MYC, NRAS, MAPK1, RAF1, VEGFA | 5.12E-18 |
| GHRLOS | hsa05205: Proteoglycans in cancer | FAS, BRAF, MAPK14, ESR1, HRAS, IGF1R, KRAS, MYC, NRAS, MAPK1, RAF1, TGFB1, TNF, VEGFA | 4.30E-14 |
| GHRLOS | hsa05163: Human cytomegalovirus infection | FAS, BAX, CDKN2A, MAPK14, HRAS, IL6R, CXCL8, KRAS, MYC, NRAS, MAPK1, RAF1, TNF, VEGFA | 1.57E-13 |
| GHRLOS | hsa05161: Hepatitis B | FAS, BAX, BCL2, BRAF, MAPK14, HRAS, CXCL8, KRAS, MYC, NRAS, MAPK1, RAF1, TGFB1, TNF, DDX58 | 4.09E-17 |
| GHRLOS | hsa05160: Hepatitis C | FAS, BAX, BRAF, HRAS, KRAS, LDLR, MYC, NRAS, MAPK1, RAF1, TNF, DDX58 | 1.08E-12 |
| GHRLOS | hsa04010: MAPK signaling pathway | FAS, BRAF, MAPK14, FGFR3, HRAS, IGF1R, KRAS, MAX, MAP3K11, MYC, NRAS, MAPK1, RAF1, TGFB1, TNF, VEGFA | 1.38E-14 |
| GHRLOS | hsa01522: Endocrine resistance | BAX, BCL2, BRAF, CDKN2A, MAPK14, ESR1, ESR2, HRAS, IGF1R, KRAS, NRAS, MAPK1, RAF1 | 6.46E-17 |
| GHRLOS | hsa01521: EGFR tyrosine kinase inhibitor resistance | BAX, BCL2, BRAF, FGFR3, HRAS, IGF1R, IL6R, KRAS, NRAS, MAPK1, RAF1, VEGFA | 2.25E-16 |
| GLIDR | hsa05161: Hepatitis B | FAS, BAX, BCL2, BRAF, MAPK14, HRAS, CXCL8, KRAS, MYC, NRAS, MAPK1, RAF1, TGFB1, TNF, DDX58 | 4.09E-17 |
| GLIDR | hsa01522: Endocrine resistance | BAX, BCL2, BRAF, CDKN2A, MAPK14, ESR1, ESR2, HRAS, IGF1R, KRAS, NRAS, MAPK1, RAF1 | 6.46E-17 |
| GLIDR | hsa01521: EGFR tyrosine kinase inhibitor resistance | BAX, BCL2, BRAF, FGFR3, HRAS, IGF1R, IL6R, KRAS, NRAS, MAPK1, RAF1, VEGFA | 2.25E-16 |
| HAR1A | hsa05219: Bladder cancer | BRAF, CDKN2A, FGFR3, HRAS, CXCL8, KRAS, MYC, NRAS, MAPK1, RAF1, VEGFA | 5.12E-18 |
| HAR1A | hsa05205: Proteoglycans in cancer | FAS, BRAF, MAPK14, ESR1, HRAS, IGF1R, KRAS, MYC, NRAS, MAPK1, RAF1, TGFB1, TNF, VEGFA | 4.30E-14 |
| HAR1A | hsa05163: Human cytomegalovirus infection | FAS, BAX, CDKN2A, MAPK14, HRAS, IL6R, CXCL8, KRAS, MYC, NRAS, MAPK1, RAF1, TNF, VEGFA | 1.57E-13 |
| HAR1A | hsa05161: Hepatitis B | FAS, BAX, BCL2, BRAF, MAPK14, HRAS, CXCL8, KRAS, MYC, NRAS, MAPK1, RAF1, TGFB1, TNF, DDX58 | 4.09E-17 |
| HAR1A | hsa05160: Hepatitis C | FAS, BAX, BRAF, HRAS, KRAS, LDLR, MYC, NRAS, MAPK1, RAF1, TNF, DDX58 | 1.08E-12 |
| HAR1A | hsa04010: MAPK signaling pathway | FAS, BRAF, MAPK14, FGFR3, HRAS, IGF1R, KRAS, MAX, MAP3K11, MYC, NRAS, MAPK1, RAF1, TGFB1, TNF, VEGFA | 1.38E-14 |
| HAR1A | hsa01522: Endocrine resistance | BAX, BCL2, BRAF, CDKN2A, MAPK14, ESR1, ESR2, HRAS, IGF1R, KRAS, NRAS, MAPK1, RAF1 | 6.46E-17 |
| HAR1A | hsa01521: EGFR tyrosine kinase inhibitor resistance | BAX, BCL2, BRAF, FGFR3, HRAS, IGF1R, IL6R, KRAS, NRAS, MAPK1, RAF1, VEGFA | 2.25E-16 |
| HCG11 | hsa05219: Bladder cancer | BRAF, CDKN2A, FGFR3, HRAS, CXCL8, KRAS, MYC, NRAS, MAPK1, RAF1, VEGFA | 5.12E-18 |
| HCG11 | hsa05205: Proteoglycans in cancer | FAS, BRAF, MAPK14, ESR1, HRAS, IGF1R, KRAS, MYC, NRAS, MAPK1, RAF1, TGFB1, TNF, VEGFA | 4.30E-14 |
| HCG11 | hsa05163: Human cytomegalovirus infection | FAS, BAX, CDKN2A, MAPK14, HRAS, IL6R, CXCL8, KRAS, MYC, NRAS, MAPK1, RAF1, TNF, VEGFA | 1.57E-13 |
| HCG11 | hsa05161: Hepatitis B | FAS, BAX, BCL2, BRAF, MAPK14, HRAS, CXCL8, KRAS, MYC, NRAS, MAPK1, RAF1, TGFB1, TNF, DDX58 | 4.09E-17 |
| HCG11 | hsa05160: Hepatitis C | FAS, BAX, BRAF, HRAS, KRAS, LDLR, MYC, NRAS, MAPK1, RAF1, TNF, DDX58 | 1.08E-12 |
| HCG11 | hsa04010: MAPK signaling pathway | FAS, BRAF, MAPK14, FGFR3, HRAS, IGF1R, KRAS, MAX, MAP3K11, MYC, NRAS, MAPK1, RAF1, TGFB1, TNF, VEGFA | 1.38E-14 |
| HCG11 | hsa01522: Endocrine resistance | BAX, BCL2, BRAF, CDKN2A, MAPK14, ESR1, ESR2, HRAS, IGF1R, KRAS, NRAS, MAPK1, RAF1 | 6.46E-17 |
| HCG11 | hsa01521: EGFR tyrosine kinase inhibitor resistance | BAX, BCL2, BRAF, FGFR3, HRAS, IGF1R, IL6R, KRAS, NRAS, MAPK1, RAF1, VEGFA | 2.25E-16 |
| HCP5 | hsa05219: Bladder cancer | BRAF, CDKN2A, FGFR3, HRAS, CXCL8, KRAS, MYC, NRAS, MAPK1, RAF1, VEGFA | 5.12E-18 |
| HCP5 | hsa05205: Proteoglycans in cancer | FAS, BRAF, MAPK14, ESR1, HRAS, IGF1R, KRAS, MYC, NRAS, MAPK1, RAF1, TGFB1, TNF, VEGFA | 4.30E-14 |
| HCP5 | hsa05163: Human cytomegalovirus infection | FAS, BAX, CDKN2A, MAPK14, HRAS, IL6R, CXCL8, KRAS, MYC, NRAS, MAPK1, RAF1, TNF, VEGFA | 1.57E-13 |
| HCP5 | hsa05161: Hepatitis B | FAS, BAX, BCL2, BRAF, MAPK14, HRAS, CXCL8, KRAS, MYC, NRAS, MAPK1, RAF1, TGFB1, TNF, DDX58 | 4.09E-17 |
| HCP5 | hsa05160: Hepatitis C | FAS, BAX, BRAF, HRAS, KRAS, LDLR, MYC, NRAS, MAPK1, RAF1, TNF, DDX58 | 1.08E-12 |
| HCP5 | hsa04010: MAPK signaling pathway | FAS, BRAF, MAPK14, FGFR3, HRAS, IGF1R, KRAS, MAX, MAP3K11, MYC, NRAS, MAPK1, RAF1, TGFB1, TNF, VEGFA | 1.38E-14 |
| HCP5 | hsa01522: Endocrine resistance | BAX, BCL2, BRAF, CDKN2A, MAPK14, ESR1, ESR2, HRAS, IGF1R, KRAS, NRAS, MAPK1, RAF1 | 6.46E-17 |
| HCP5 | hsa01521: EGFR tyrosine kinase inhibitor resistance | BAX, BCL2, BRAF, FGFR3, HRAS, IGF1R, IL6R, KRAS, NRAS, MAPK1, RAF1, VEGFA | 2.25E-16 |
| HEXA-AS1 | hsa05219: Bladder cancer | BRAF, CDKN2A, FGFR3, HRAS, CXCL8, KRAS, MYC, NRAS, MAPK1, RAF1, VEGFA | 5.12E-18 |
| HEXA-AS1 | hsa05205: Proteoglycans in cancer | FAS, BRAF, MAPK14, ESR1, HRAS, IGF1R, KRAS, MYC, NRAS, MAPK1, RAF1, TGFB1, TNF, VEGFA | 4.30E-14 |
| HEXA-AS1 | hsa05163: Human cytomegalovirus infection | FAS, BAX, CDKN2A, MAPK14, HRAS, IL6R, CXCL8, KRAS, MYC, NRAS, MAPK1, RAF1, TNF, VEGFA | 1.57E-13 |
| HEXA-AS1 | hsa05161: Hepatitis B | FAS, BAX, BCL2, BRAF, MAPK14, HRAS, CXCL8, KRAS, MYC, NRAS, MAPK1, RAF1, TGFB1, TNF, DDX58 | 4.09E-17 |
| HEXA-AS1 | hsa05160: Hepatitis C | FAS, BAX, BRAF, HRAS, KRAS, LDLR, MYC, NRAS, MAPK1, RAF1, TNF, DDX58 | 1.08E-12 |
| HEXA-AS1 | hsa04010: MAPK signaling pathway | FAS, BRAF, MAPK14, FGFR3, HRAS, IGF1R, KRAS, MAX, MAP3K11, MYC, NRAS, MAPK1, RAF1, TGFB1, TNF, VEGFA | 1.38E-14 |
| HEXA-AS1 | hsa01522: Endocrine resistance | BAX, BCL2, BRAF, CDKN2A, MAPK14, ESR1, ESR2, HRAS, IGF1R, KRAS, NRAS, MAPK1, RAF1 | 6.46E-17 |
| HEXA-AS1 | hsa01521: EGFR tyrosine kinase inhibitor resistance | BAX, BCL2, BRAF, FGFR3, HRAS, IGF1R, IL6R, KRAS, NRAS, MAPK1, RAF1, VEGFA | 2.25E-16 |
| IDI2-AS1 | hsa05219: Bladder cancer | BRAF, CDKN2A, FGFR3, HRAS, CXCL8, KRAS, MYC, NRAS, MAPK1, RAF1, VEGFA | 5.12E-18 |
| IDI2-AS1 | hsa05205: Proteoglycans in cancer | FAS, BRAF, MAPK14, ESR1, HRAS, IGF1R, KRAS, MYC, NRAS, MAPK1, RAF1, TGFB1, TNF, VEGFA | 4.30E-14 |
| IDI2-AS1 | hsa05161: Hepatitis B | FAS, BAX, BCL2, BRAF, MAPK14, HRAS, CXCL8, KRAS, MYC, NRAS, MAPK1, RAF1, TGFB1, TNF, DDX58 | 4.09E-17 |
| IDI2-AS1 | hsa05160: Hepatitis C | FAS, BAX, BRAF, HRAS, KRAS, LDLR, MYC, NRAS, MAPK1, RAF1, TNF, DDX58 | 1.08E-12 |
| IDI2-AS1 | hsa04010: MAPK signaling pathway | FAS, BRAF, MAPK14, FGFR3, HRAS, IGF1R, KRAS, MAX, MAP3K11, MYC, NRAS, MAPK1, RAF1, TGFB1, TNF, VEGFA | 1.38E-14 |
| IDI2-AS1 | hsa01522: Endocrine resistance | BAX, BCL2, BRAF, CDKN2A, MAPK14, ESR1, ESR2, HRAS, IGF1R, KRAS, NRAS, MAPK1, RAF1 | 6.46E-17 |
| IDI2-AS1 | hsa01521: EGFR tyrosine kinase inhibitor resistance | BAX, BCL2, BRAF, FGFR3, HRAS, IGF1R, IL6R, KRAS, NRAS, MAPK1, RAF1, VEGFA | 2.25E-16 |
| INE1 | hsa05219: Bladder cancer | BRAF, CDKN2A, FGFR3, HRAS, CXCL8, KRAS, MYC, NRAS, MAPK1, RAF1, VEGFA | 5.12E-18 |
| INE1 | hsa05205: Proteoglycans in cancer | FAS, BRAF, MAPK14, ESR1, HRAS, IGF1R, KRAS, MYC, NRAS, MAPK1, RAF1, TGFB1, TNF, VEGFA | 4.30E-14 |
| INE1 | hsa05163: Human cytomegalovirus infection | FAS, BAX, CDKN2A, MAPK14, HRAS, IL6R, CXCL8, KRAS, MYC, NRAS, MAPK1, RAF1, TNF, VEGFA | 1.57E-13 |
| INE1 | hsa05161: Hepatitis B | FAS, BAX, BCL2, BRAF, MAPK14, HRAS, CXCL8, KRAS, MYC, NRAS, MAPK1, RAF1, TGFB1, TNF, DDX58 | 4.09E-17 |
| INE1 | hsa05160: Hepatitis C | FAS, BAX, BRAF, HRAS, KRAS, LDLR, MYC, NRAS, MAPK1, RAF1, TNF, DDX58 | 1.08E-12 |
| INE1 | hsa04010: MAPK signaling pathway | FAS, BRAF, MAPK14, FGFR3, HRAS, IGF1R, KRAS, MAX, MAP3K11, MYC, NRAS, MAPK1, RAF1, TGFB1, TNF, VEGFA | 1.38E-14 |
| INE1 | hsa01522: Endocrine resistance | BAX, BCL2, BRAF, CDKN2A, MAPK14, ESR1, ESR2, HRAS, IGF1R, KRAS, NRAS, MAPK1, RAF1 | 6.46E-17 |
| INE1 | hsa01521: EGFR tyrosine kinase inhibitor resistance | BAX, BCL2, BRAF, FGFR3, HRAS, IGF1R, IL6R, KRAS, NRAS, MAPK1, RAF1, VEGFA | 2.25E-16 |
| JPX | hsa05205: Proteoglycans in cancer | FAS, BRAF, MAPK14, ESR1, HRAS, IGF1R, KRAS, MYC, NRAS, MAPK1, RAF1, TGFB1, TNF, VEGFA | 4.30E-14 |
| JPX | hsa04010: MAPK signaling pathway | FAS, BRAF, MAPK14, FGFR3, HRAS, IGF1R, KRAS, MAX, MAP3K11, MYC, NRAS, MAPK1, RAF1, TGFB1, TNF, VEGFA | 1.38E-14 |
| JPX | hsa01522: Endocrine resistance | BAX, BCL2, BRAF, CDKN2A, MAPK14, ESR1, ESR2, HRAS, IGF1R, KRAS, NRAS, MAPK1, RAF1 | 6.46E-17 |
| KIF9-AS1 | hsa05219: Bladder cancer | BRAF, CDKN2A, FGFR3, HRAS, CXCL8, KRAS, MYC, NRAS, MAPK1, RAF1, VEGFA | 5.12E-18 |
| KIF9-AS1 | hsa05205: Proteoglycans in cancer | FAS, BRAF, MAPK14, ESR1, HRAS, IGF1R, KRAS, MYC, NRAS, MAPK1, RAF1, TGFB1, TNF, VEGFA | 4.30E-14 |
| KIF9-AS1 | hsa05163: Human cytomegalovirus infection | FAS, BAX, CDKN2A, MAPK14, HRAS, IL6R, CXCL8, KRAS, MYC, NRAS, MAPK1, RAF1, TNF, VEGFA | 1.57E-13 |
| KIF9-AS1 | hsa05161: Hepatitis B | FAS, BAX, BCL2, BRAF, MAPK14, HRAS, CXCL8, KRAS, MYC, NRAS, MAPK1, RAF1, TGFB1, TNF, DDX58 | 4.09E-17 |
| KIF9-AS1 | hsa05160: Hepatitis C | FAS, BAX, BRAF, HRAS, KRAS, LDLR, MYC, NRAS, MAPK1, RAF1, TNF, DDX58 | 1.08E-12 |
| KIF9-AS1 | hsa04010: MAPK signaling pathway | FAS, BRAF, MAPK14, FGFR3, HRAS, IGF1R, KRAS, MAX, MAP3K11, MYC, NRAS, MAPK1, RAF1, TGFB1, TNF, VEGFA | 1.38E-14 |
| KIF9-AS1 | hsa01522: Endocrine resistance | BAX, BCL2, BRAF, CDKN2A, MAPK14, ESR1, ESR2, HRAS, IGF1R, KRAS, NRAS, MAPK1, RAF1 | 6.46E-17 |
| KIF9-AS1 | hsa01521: EGFR tyrosine kinase inhibitor resistance | BAX, BCL2, BRAF, FGFR3, HRAS, IGF1R, IL6R, KRAS, NRAS, MAPK1, RAF1, VEGFA | 2.25E-16 |
| KRTAP5-AS1 | hsa05205: Proteoglycans in cancer | FAS, BRAF, MAPK14, ESR1, HRAS, IGF1R, KRAS, MYC, NRAS, MAPK1, RAF1, TGFB1, TNF, VEGFA | 4.30E-14 |
| KRTAP5-AS1 | hsa05163: Human cytomegalovirus infection | FAS, BAX, CDKN2A, MAPK14, HRAS, IL6R, CXCL8, KRAS, MYC, NRAS, MAPK1, RAF1, TNF, VEGFA | 1.57E-13 |
| KRTAP5-AS1 | hsa05161: Hepatitis B | FAS, BAX, BCL2, BRAF, MAPK14, HRAS, CXCL8, KRAS, MYC, NRAS, MAPK1, RAF1, TGFB1, TNF, DDX58 | 4.09E-17 |
| KRTAP5-AS1 | hsa05160: Hepatitis C | FAS, BAX, BRAF, HRAS, KRAS, LDLR, MYC, NRAS, MAPK1, RAF1, TNF, DDX58 | 1.08E-12 |
| KRTAP5-AS1 | hsa04010: MAPK signaling pathway | FAS, BRAF, MAPK14, FGFR3, HRAS, IGF1R, KRAS, MAX, MAP3K11, MYC, NRAS, MAPK1, RAF1, TGFB1, TNF, VEGFA | 1.38E-14 |
| KRTAP5-AS1 | hsa01522: Endocrine resistance | BAX, BCL2, BRAF, CDKN2A, MAPK14, ESR1, ESR2, HRAS, IGF1R, KRAS, NRAS, MAPK1, RAF1 | 6.46E-17 |
| KRTAP5-AS1 | hsa01521: EGFR tyrosine kinase inhibitor resistance | BAX, BCL2, BRAF, FGFR3, HRAS, IGF1R, IL6R, KRAS, NRAS, MAPK1, RAF1, VEGFA | 2.25E-16 |
| LINC00173 | hsa05219: Bladder cancer | BRAF, CDKN2A, FGFR3, HRAS, CXCL8, KRAS, MYC, NRAS, MAPK1, RAF1, VEGFA | 5.12E-18 |
| LINC00173 | hsa05205: Proteoglycans in cancer | FAS, BRAF, MAPK14, ESR1, HRAS, IGF1R, KRAS, MYC, NRAS, MAPK1, RAF1, TGFB1, TNF, VEGFA | 4.30E-14 |
| LINC00173 | hsa05163: Human cytomegalovirus infection | FAS, BAX, CDKN2A, MAPK14, HRAS, IL6R, CXCL8, KRAS, MYC, NRAS, MAPK1, RAF1, TNF, VEGFA | 1.57E-13 |
| LINC00173 | hsa05161: Hepatitis B | FAS, BAX, BCL2, BRAF, MAPK14, HRAS, CXCL8, KRAS, MYC, NRAS, MAPK1, RAF1, TGFB1, TNF, DDX58 | 4.09E-17 |
| LINC00173 | hsa05160: Hepatitis C | FAS, BAX, BRAF, HRAS, KRAS, LDLR, MYC, NRAS, MAPK1, RAF1, TNF, DDX58 | 1.08E-12 |
| LINC00173 | hsa04010: MAPK signaling pathway | FAS, BRAF, MAPK14, FGFR3, HRAS, IGF1R, KRAS, MAX, MAP3K11, MYC, NRAS, MAPK1, RAF1, TGFB1, TNF, VEGFA | 1.38E-14 |
| LINC00173 | hsa01522: Endocrine resistance | BAX, BCL2, BRAF, CDKN2A, MAPK14, ESR1, ESR2, HRAS, IGF1R, KRAS, NRAS, MAPK1, RAF1 | 6.46E-17 |
| LINC00173 | hsa01521: EGFR tyrosine kinase inhibitor resistance | BAX, BCL2, BRAF, FGFR3, HRAS, IGF1R, IL6R, KRAS, NRAS, MAPK1, RAF1, VEGFA | 2.25E-16 |
| LINC00265 | hsa05219: Bladder cancer | BRAF, CDKN2A, FGFR3, HRAS, CXCL8, KRAS, MYC, NRAS, MAPK1, RAF1, VEGFA | 5.12E-18 |
| LINC00265 | hsa05205: Proteoglycans in cancer | FAS, BRAF, MAPK14, ESR1, HRAS, IGF1R, KRAS, MYC, NRAS, MAPK1, RAF1, TGFB1, TNF, VEGFA | 4.30E-14 |
| LINC00265 | hsa05163: Human cytomegalovirus infection | FAS, BAX, CDKN2A, MAPK14, HRAS, IL6R, CXCL8, KRAS, MYC, NRAS, MAPK1, RAF1, TNF, VEGFA | 1.57E-13 |
| LINC00265 | hsa05161: Hepatitis B | FAS, BAX, BCL2, BRAF, MAPK14, HRAS, CXCL8, KRAS, MYC, NRAS, MAPK1, RAF1, TGFB1, TNF, DDX58 | 4.09E-17 |
| LINC00265 | hsa05160: Hepatitis C | FAS, BAX, BRAF, HRAS, KRAS, LDLR, MYC, NRAS, MAPK1, RAF1, TNF, DDX58 | 1.08E-12 |
| LINC00265 | hsa04010: MAPK signaling pathway | FAS, BRAF, MAPK14, FGFR3, HRAS, IGF1R, KRAS, MAX, MAP3K11, MYC, NRAS, MAPK1, RAF1, TGFB1, TNF, VEGFA | 1.38E-14 |
| LINC00265 | hsa01522: Endocrine resistance | BAX, BCL2, BRAF, CDKN2A, MAPK14, ESR1, ESR2, HRAS, IGF1R, KRAS, NRAS, MAPK1, RAF1 | 6.46E-17 |
| LINC00265 | hsa01521: EGFR tyrosine kinase inhibitor resistance | BAX, BCL2, BRAF, FGFR3, HRAS, IGF1R, IL6R, KRAS, NRAS, MAPK1, RAF1, VEGFA | 2.25E-16 |
| LINC00282 | hsa05205: Proteoglycans in cancer | FAS, BRAF, MAPK14, ESR1, HRAS, IGF1R, KRAS, MYC, NRAS, MAPK1, RAF1, TGFB1, TNF, VEGFA | 4.30E-14 |
| LINC00282 | hsa05163: Human cytomegalovirus infection | FAS, BAX, CDKN2A, MAPK14, HRAS, IL6R, CXCL8, KRAS, MYC, NRAS, MAPK1, RAF1, TNF, VEGFA | 1.57E-13 |
| LINC00282 | hsa05161: Hepatitis B | FAS, BAX, BCL2, BRAF, MAPK14, HRAS, CXCL8, KRAS, MYC, NRAS, MAPK1, RAF1, TGFB1, TNF, DDX58 | 4.09E-17 |
| LINC00282 | hsa04010: MAPK signaling pathway | FAS, BRAF, MAPK14, FGFR3, HRAS, IGF1R, KRAS, MAX, MAP3K11, MYC, NRAS, MAPK1, RAF1, TGFB1, TNF, VEGFA | 1.38E-14 |
| LINC00282 | hsa01522: Endocrine resistance | BAX, BCL2, BRAF, CDKN2A, MAPK14, ESR1, ESR2, HRAS, IGF1R, KRAS, NRAS, MAPK1, RAF1 | 6.46E-17 |
| LINC00294 | hsa05219: Bladder cancer | BRAF, CDKN2A, FGFR3, HRAS, CXCL8, KRAS, MYC, NRAS, MAPK1, RAF1, VEGFA | 5.12E-18 |
| LINC00294 | hsa05205: Proteoglycans in cancer | FAS, BRAF, MAPK14, ESR1, HRAS, IGF1R, KRAS, MYC, NRAS, MAPK1, RAF1, TGFB1, TNF, VEGFA | 4.30E-14 |
| LINC00294 | hsa05163: Human cytomegalovirus infection | FAS, BAX, CDKN2A, MAPK14, HRAS, IL6R, CXCL8, KRAS, MYC, NRAS, MAPK1, RAF1, TNF, VEGFA | 1.57E-13 |
| LINC00294 | hsa05161: Hepatitis B | FAS, BAX, BCL2, BRAF, MAPK14, HRAS, CXCL8, KRAS, MYC, NRAS, MAPK1, RAF1, TGFB1, TNF, DDX58 | 4.09E-17 |
| LINC00294 | hsa05160: Hepatitis C | FAS, BAX, BRAF, HRAS, KRAS, LDLR, MYC, NRAS, MAPK1, RAF1, TNF, DDX58 | 1.08E-12 |
| LINC00294 | hsa04010: MAPK signaling pathway | FAS, BRAF, MAPK14, FGFR3, HRAS, IGF1R, KRAS, MAX, MAP3K11, MYC, NRAS, MAPK1, RAF1, TGFB1, TNF, VEGFA | 1.38E-14 |
| LINC00294 | hsa01522: Endocrine resistance | BAX, BCL2, BRAF, CDKN2A, MAPK14, ESR1, ESR2, HRAS, IGF1R, KRAS, NRAS, MAPK1, RAF1 | 6.46E-17 |
| LINC00294 | hsa01521: EGFR tyrosine kinase inhibitor resistance | BAX, BCL2, BRAF, FGFR3, HRAS, IGF1R, IL6R, KRAS, NRAS, MAPK1, RAF1, VEGFA | 2.25E-16 |
| LINC00304 | hsa05163: Human cytomegalovirus infection | FAS, BAX, CDKN2A, MAPK14, HRAS, IL6R, CXCL8, KRAS, MYC, NRAS, MAPK1, RAF1, TNF, VEGFA | 1.57E-13 |
| LINC00304 | hsa05161: Hepatitis B | FAS, BAX, BCL2, BRAF, MAPK14, HRAS, CXCL8, KRAS, MYC, NRAS, MAPK1, RAF1, TGFB1, TNF, DDX58 | 4.09E-17 |
| LINC00304 | hsa05160: Hepatitis C | FAS, BAX, BRAF, HRAS, KRAS, LDLR, MYC, NRAS, MAPK1, RAF1, TNF, DDX58 | 1.08E-12 |
| LINC00304 | hsa01522: Endocrine resistance | BAX, BCL2, BRAF, CDKN2A, MAPK14, ESR1, ESR2, HRAS, IGF1R, KRAS, NRAS, MAPK1, RAF1 | 6.46E-17 |
| LINC00304 | hsa01521: EGFR tyrosine kinase inhibitor resistance | BAX, BCL2, BRAF, FGFR3, HRAS, IGF1R, IL6R, KRAS, NRAS, MAPK1, RAF1, VEGFA | 2.25E-16 |
| LINC00310 | hsa05163: Human cytomegalovirus infection | FAS, BAX, CDKN2A, MAPK14, HRAS, IL6R, CXCL8, KRAS, MYC, NRAS, MAPK1, RAF1, TNF, VEGFA | 1.57E-13 |
| LINC00310 | hsa05161: Hepatitis B | FAS, BAX, BCL2, BRAF, MAPK14, HRAS, CXCL8, KRAS, MYC, NRAS, MAPK1, RAF1, TGFB1, TNF, DDX58 | 4.09E-17 |
| LINC00310 | hsa05160: Hepatitis C | FAS, BAX, BRAF, HRAS, KRAS, LDLR, MYC, NRAS, MAPK1, RAF1, TNF, DDX58 | 1.08E-12 |
| LINC00310 | hsa01522: Endocrine resistance | BAX, BCL2, BRAF, CDKN2A, MAPK14, ESR1, ESR2, HRAS, IGF1R, KRAS, NRAS, MAPK1, RAF1 | 6.46E-17 |
| LINC00310 | hsa01521: EGFR tyrosine kinase inhibitor resistance | BAX, BCL2, BRAF, FGFR3, HRAS, IGF1R, IL6R, KRAS, NRAS, MAPK1, RAF1, VEGFA | 2.25E-16 |
| LINC00324 | hsa05205: Proteoglycans in cancer | FAS, BRAF, MAPK14, ESR1, HRAS, IGF1R, KRAS, MYC, NRAS, MAPK1, RAF1, TGFB1, TNF, VEGFA | 4.30E-14 |
| LINC00324 | hsa05163: Human cytomegalovirus infection | FAS, BAX, CDKN2A, MAPK14, HRAS, IL6R, CXCL8, KRAS, MYC, NRAS, MAPK1, RAF1, TNF, VEGFA | 1.57E-13 |
| LINC00324 | hsa05161: Hepatitis B | FAS, BAX, BCL2, BRAF, MAPK14, HRAS, CXCL8, KRAS, MYC, NRAS, MAPK1, RAF1, TGFB1, TNF, DDX58 | 4.09E-17 |
| LINC00324 | hsa05160: Hepatitis C | FAS, BAX, BRAF, HRAS, KRAS, LDLR, MYC, NRAS, MAPK1, RAF1, TNF, DDX58 | 1.08E-12 |
| LINC00324 | hsa04010: MAPK signaling pathway | FAS, BRAF, MAPK14, FGFR3, HRAS, IGF1R, KRAS, MAX, MAP3K11, MYC, NRAS, MAPK1, RAF1, TGFB1, TNF, VEGFA | 1.38E-14 |
| LINC00324 | hsa01521: EGFR tyrosine kinase inhibitor resistance | BAX, BCL2, BRAF, FGFR3, HRAS, IGF1R, IL6R, KRAS, NRAS, MAPK1, RAF1, VEGFA | 2.25E-16 |
| LINC00339 | hsa05219: Bladder cancer | BRAF, CDKN2A, FGFR3, HRAS, CXCL8, KRAS, MYC, NRAS, MAPK1, RAF1, VEGFA | 5.12E-18 |
| LINC00339 | hsa05205: Proteoglycans in cancer | FAS, BRAF, MAPK14, ESR1, HRAS, IGF1R, KRAS, MYC, NRAS, MAPK1, RAF1, TGFB1, TNF, VEGFA | 4.30E-14 |
| LINC00339 | hsa05163: Human cytomegalovirus infection | FAS, BAX, CDKN2A, MAPK14, HRAS, IL6R, CXCL8, KRAS, MYC, NRAS, MAPK1, RAF1, TNF, VEGFA | 1.57E-13 |
| LINC00339 | hsa05161: Hepatitis B | FAS, BAX, BCL2, BRAF, MAPK14, HRAS, CXCL8, KRAS, MYC, NRAS, MAPK1, RAF1, TGFB1, TNF, DDX58 | 4.09E-17 |
| LINC00339 | hsa05160: Hepatitis C | FAS, BAX, BRAF, HRAS, KRAS, LDLR, MYC, NRAS, MAPK1, RAF1, TNF, DDX58 | 1.08E-12 |
| LINC00339 | hsa04010: MAPK signaling pathway | FAS, BRAF, MAPK14, FGFR3, HRAS, IGF1R, KRAS, MAX, MAP3K11, MYC, NRAS, MAPK1, RAF1, TGFB1, TNF, VEGFA | 1.38E-14 |
| LINC00339 | hsa01522: Endocrine resistance | BAX, BCL2, BRAF, CDKN2A, MAPK14, ESR1, ESR2, HRAS, IGF1R, KRAS, NRAS, MAPK1, RAF1 | 6.46E-17 |
| LINC00339 | hsa01521: EGFR tyrosine kinase inhibitor resistance | BAX, BCL2, BRAF, FGFR3, HRAS, IGF1R, IL6R, KRAS, NRAS, MAPK1, RAF1, VEGFA | 2.25E-16 |
| LINC00467 | hsa05219: Bladder cancer | BRAF, CDKN2A, FGFR3, HRAS, CXCL8, KRAS, MYC, NRAS, MAPK1, RAF1, VEGFA | 5.12E-18 |
| LINC00467 | hsa05205: Proteoglycans in cancer | FAS, BRAF, MAPK14, ESR1, HRAS, IGF1R, KRAS, MYC, NRAS, MAPK1, RAF1, TGFB1, TNF, VEGFA | 4.30E-14 |
| LINC00467 | hsa05163: Human cytomegalovirus infection | FAS, BAX, CDKN2A, MAPK14, HRAS, IL6R, CXCL8, KRAS, MYC, NRAS, MAPK1, RAF1, TNF, VEGFA | 1.57E-13 |
| LINC00467 | hsa05161: Hepatitis B | FAS, BAX, BCL2, BRAF, MAPK14, HRAS, CXCL8, KRAS, MYC, NRAS, MAPK1, RAF1, TGFB1, TNF, DDX58 | 4.09E-17 |
| LINC00467 | hsa05160: Hepatitis C | FAS, BAX, BRAF, HRAS, KRAS, LDLR, MYC, NRAS, MAPK1, RAF1, TNF, DDX58 | 1.08E-12 |
| LINC00467 | hsa04010: MAPK signaling pathway | FAS, BRAF, MAPK14, FGFR3, HRAS, IGF1R, KRAS, MAX, MAP3K11, MYC, NRAS, MAPK1, RAF1, TGFB1, TNF, VEGFA | 1.38E-14 |
| LINC00467 | hsa01522: Endocrine resistance | BAX, BCL2, BRAF, CDKN2A, MAPK14, ESR1, ESR2, HRAS, IGF1R, KRAS, NRAS, MAPK1, RAF1 | 6.46E-17 |
| LINC00467 | hsa01521: EGFR tyrosine kinase inhibitor resistance | BAX, BCL2, BRAF, FGFR3, HRAS, IGF1R, IL6R, KRAS, NRAS, MAPK1, RAF1, VEGFA | 2.25E-16 |
| LINC00469 | hsa05161: Hepatitis B | FAS, BAX, BCL2, BRAF, MAPK14, HRAS, CXCL8, KRAS, MYC, NRAS, MAPK1, RAF1, TGFB1, TNF, DDX58 | 4.09E-17 |
| LINC00469 | hsa01522: Endocrine resistance | BAX, BCL2, BRAF, CDKN2A, MAPK14, ESR1, ESR2, HRAS, IGF1R, KRAS, NRAS, MAPK1, RAF1 | 6.46E-17 |
| LINC00469 | hsa01521: EGFR tyrosine kinase inhibitor resistance | BAX, BCL2, BRAF, FGFR3, HRAS, IGF1R, IL6R, KRAS, NRAS, MAPK1, RAF1, VEGFA | 2.25E-16 |
| LINC00484 | hsa05219: Bladder cancer | BRAF, CDKN2A, FGFR3, HRAS, CXCL8, KRAS, MYC, NRAS, MAPK1, RAF1, VEGFA | 5.12E-18 |
| LINC00484 | hsa05163: Human cytomegalovirus infection | FAS, BAX, CDKN2A, MAPK14, HRAS, IL6R, CXCL8, KRAS, MYC, NRAS, MAPK1, RAF1, TNF, VEGFA | 1.57E-13 |
| LINC00484 | hsa01522: Endocrine resistance | BAX, BCL2, BRAF, CDKN2A, MAPK14, ESR1, ESR2, HRAS, IGF1R, KRAS, NRAS, MAPK1, RAF1 | 6.46E-17 |
| LINC00593 | hsa05205: Proteoglycans in cancer | FAS, BRAF, MAPK14, ESR1, HRAS, IGF1R, KRAS, MYC, NRAS, MAPK1, RAF1, TGFB1, TNF, VEGFA | 4.30E-14 |
| LINC00593 | hsa05163: Human cytomegalovirus infection | FAS, BAX, CDKN2A, MAPK14, HRAS, IL6R, CXCL8, KRAS, MYC, NRAS, MAPK1, RAF1, TNF, VEGFA | 1.57E-13 |
| LINC00593 | hsa05161: Hepatitis B | FAS, BAX, BCL2, BRAF, MAPK14, HRAS, CXCL8, KRAS, MYC, NRAS, MAPK1, RAF1, TGFB1, TNF, DDX58 | 4.09E-17 |
| LINC00593 | hsa05160: Hepatitis C | FAS, BAX, BRAF, HRAS, KRAS, LDLR, MYC, NRAS, MAPK1, RAF1, TNF, DDX58 | 1.08E-12 |
| LINC00593 | hsa04010: MAPK signaling pathway | FAS, BRAF, MAPK14, FGFR3, HRAS, IGF1R, KRAS, MAX, MAP3K11, MYC, NRAS, MAPK1, RAF1, TGFB1, TNF, VEGFA | 1.38E-14 |
| LINC00593 | hsa01521: EGFR tyrosine kinase inhibitor resistance | BAX, BCL2, BRAF, FGFR3, HRAS, IGF1R, IL6R, KRAS, NRAS, MAPK1, RAF1, VEGFA | 2.25E-16 |
| LINC00664 | hsa05219: Bladder cancer | BRAF, CDKN2A, FGFR3, HRAS, CXCL8, KRAS, MYC, NRAS, MAPK1, RAF1, VEGFA | 5.12E-18 |
| LINC00664 | hsa05205: Proteoglycans in cancer | FAS, BRAF, MAPK14, ESR1, HRAS, IGF1R, KRAS, MYC, NRAS, MAPK1, RAF1, TGFB1, TNF, VEGFA | 4.30E-14 |
| LINC00664 | hsa05163: Human cytomegalovirus infection | FAS, BAX, CDKN2A, MAPK14, HRAS, IL6R, CXCL8, KRAS, MYC, NRAS, MAPK1, RAF1, TNF, VEGFA | 1.57E-13 |
| LINC00664 | hsa04010: MAPK signaling pathway | FAS, BRAF, MAPK14, FGFR3, HRAS, IGF1R, KRAS, MAX, MAP3K11, MYC, NRAS, MAPK1, RAF1, TGFB1, TNF, VEGFA | 1.38E-14 |
| LINC00664 | hsa01521: EGFR tyrosine kinase inhibitor resistance | BAX, BCL2, BRAF, FGFR3, HRAS, IGF1R, IL6R, KRAS, NRAS, MAPK1, RAF1, VEGFA | 2.25E-16 |
| LINC00667 | hsa05219: Bladder cancer | BRAF, CDKN2A, FGFR3, HRAS, CXCL8, KRAS, MYC, NRAS, MAPK1, RAF1, VEGFA | 5.12E-18 |
| LINC00667 | hsa05205: Proteoglycans in cancer | FAS, BRAF, MAPK14, ESR1, HRAS, IGF1R, KRAS, MYC, NRAS, MAPK1, RAF1, TGFB1, TNF, VEGFA | 4.30E-14 |
| LINC00667 | hsa05163: Human cytomegalovirus infection | FAS, BAX, CDKN2A, MAPK14, HRAS, IL6R, CXCL8, KRAS, MYC, NRAS, MAPK1, RAF1, TNF, VEGFA | 1.57E-13 |
| LINC00667 | hsa05161: Hepatitis B | FAS, BAX, BCL2, BRAF, MAPK14, HRAS, CXCL8, KRAS, MYC, NRAS, MAPK1, RAF1, TGFB1, TNF, DDX58 | 4.09E-17 |
| LINC00667 | hsa05160: Hepatitis C | FAS, BAX, BRAF, HRAS, KRAS, LDLR, MYC, NRAS, MAPK1, RAF1, TNF, DDX58 | 1.08E-12 |
| LINC00667 | hsa04010: MAPK signaling pathway | FAS, BRAF, MAPK14, FGFR3, HRAS, IGF1R, KRAS, MAX, MAP3K11, MYC, NRAS, MAPK1, RAF1, TGFB1, TNF, VEGFA | 1.38E-14 |
| LINC00667 | hsa01522: Endocrine resistance | BAX, BCL2, BRAF, CDKN2A, MAPK14, ESR1, ESR2, HRAS, IGF1R, KRAS, NRAS, MAPK1, RAF1 | 6.46E-17 |
| LINC00667 | hsa01521: EGFR tyrosine kinase inhibitor resistance | BAX, BCL2, BRAF, FGFR3, HRAS, IGF1R, IL6R, KRAS, NRAS, MAPK1, RAF1, VEGFA | 2.25E-16 |
| LINC00671 | hsa05205: Proteoglycans in cancer | FAS, BRAF, MAPK14, ESR1, HRAS, IGF1R, KRAS, MYC, NRAS, MAPK1, RAF1, TGFB1, TNF, VEGFA | 4.30E-14 |
| LINC00671 | hsa05163: Human cytomegalovirus infection | FAS, BAX, CDKN2A, MAPK14, HRAS, IL6R, CXCL8, KRAS, MYC, NRAS, MAPK1, RAF1, TNF, VEGFA | 1.57E-13 |
| LINC00671 | hsa05161: Hepatitis B | FAS, BAX, BCL2, BRAF, MAPK14, HRAS, CXCL8, KRAS, MYC, NRAS, MAPK1, RAF1, TGFB1, TNF, DDX58 | 4.09E-17 |
| LINC00671 | hsa04010: MAPK signaling pathway | FAS, BRAF, MAPK14, FGFR3, HRAS, IGF1R, KRAS, MAX, MAP3K11, MYC, NRAS, MAPK1, RAF1, TGFB1, TNF, VEGFA | 1.38E-14 |
| LINC00671 | hsa01522: Endocrine resistance | BAX, BCL2, BRAF, CDKN2A, MAPK14, ESR1, ESR2, HRAS, IGF1R, KRAS, NRAS, MAPK1, RAF1 | 6.46E-17 |
| LINC00685 | hsa05205: Proteoglycans in cancer | FAS, BRAF, MAPK14, ESR1, HRAS, IGF1R, KRAS, MYC, NRAS, MAPK1, RAF1, TGFB1, TNF, VEGFA | 4.30E-14 |
| LINC00685 | hsa05163: Human cytomegalovirus infection | FAS, BAX, CDKN2A, MAPK14, HRAS, IL6R, CXCL8, KRAS, MYC, NRAS, MAPK1, RAF1, TNF, VEGFA | 1.57E-13 |
| LINC00685 | hsa05161: Hepatitis B | FAS, BAX, BCL2, BRAF, MAPK14, HRAS, CXCL8, KRAS, MYC, NRAS, MAPK1, RAF1, TGFB1, TNF, DDX58 | 4.09E-17 |
| LINC00685 | hsa04010: MAPK signaling pathway | FAS, BRAF, MAPK14, FGFR3, HRAS, IGF1R, KRAS, MAX, MAP3K11, MYC, NRAS, MAPK1, RAF1, TGFB1, TNF, VEGFA | 1.38E-14 |
| LINC00685 | hsa01522: Endocrine resistance | BAX, BCL2, BRAF, CDKN2A, MAPK14, ESR1, ESR2, HRAS, IGF1R, KRAS, NRAS, MAPK1, RAF1 | 6.46E-17 |
| LINC00847 | hsa05219: Bladder cancer | BRAF, CDKN2A, FGFR3, HRAS, CXCL8, KRAS, MYC, NRAS, MAPK1, RAF1, VEGFA | 5.12E-18 |
| LINC00847 | hsa05205: Proteoglycans in cancer | FAS, BRAF, MAPK14, ESR1, HRAS, IGF1R, KRAS, MYC, NRAS, MAPK1, RAF1, TGFB1, TNF, VEGFA | 4.30E-14 |
| LINC00847 | hsa05163: Human cytomegalovirus infection | FAS, BAX, CDKN2A, MAPK14, HRAS, IL6R, CXCL8, KRAS, MYC, NRAS, MAPK1, RAF1, TNF, VEGFA | 1.57E-13 |
| LINC00847 | hsa05161: Hepatitis B | FAS, BAX, BCL2, BRAF, MAPK14, HRAS, CXCL8, KRAS, MYC, NRAS, MAPK1, RAF1, TGFB1, TNF, DDX58 | 4.09E-17 |
| LINC00847 | hsa05160: Hepatitis C | FAS, BAX, BRAF, HRAS, KRAS, LDLR, MYC, NRAS, MAPK1, RAF1, TNF, DDX58 | 1.08E-12 |
| LINC00847 | hsa04010: MAPK signaling pathway | FAS, BRAF, MAPK14, FGFR3, HRAS, IGF1R, KRAS, MAX, MAP3K11, MYC, NRAS, MAPK1, RAF1, TGFB1, TNF, VEGFA | 1.38E-14 |
| LINC00847 | hsa01522: Endocrine resistance | BAX, BCL2, BRAF, CDKN2A, MAPK14, ESR1, ESR2, HRAS, IGF1R, KRAS, NRAS, MAPK1, RAF1 | 6.46E-17 |
| LINC00847 | hsa01521: EGFR tyrosine kinase inhibitor resistance | BAX, BCL2, BRAF, FGFR3, HRAS, IGF1R, IL6R, KRAS, NRAS, MAPK1, RAF1, VEGFA | 2.25E-16 |
| LINC00852 | hsa05219: Bladder cancer | BRAF, CDKN2A, FGFR3, HRAS, CXCL8, KRAS, MYC, NRAS, MAPK1, RAF1, VEGFA | 5.12E-18 |
| LINC00852 | hsa05205: Proteoglycans in cancer | FAS, BRAF, MAPK14, ESR1, HRAS, IGF1R, KRAS, MYC, NRAS, MAPK1, RAF1, TGFB1, TNF, VEGFA | 4.30E-14 |
| LINC00852 | hsa05163: Human cytomegalovirus infection | FAS, BAX, CDKN2A, MAPK14, HRAS, IL6R, CXCL8, KRAS, MYC, NRAS, MAPK1, RAF1, TNF, VEGFA | 1.57E-13 |
| LINC00852 | hsa05161: Hepatitis B | FAS, BAX, BCL2, BRAF, MAPK14, HRAS, CXCL8, KRAS, MYC, NRAS, MAPK1, RAF1, TGFB1, TNF, DDX58 | 4.09E-17 |
| LINC00852 | hsa05160: Hepatitis C | FAS, BAX, BRAF, HRAS, KRAS, LDLR, MYC, NRAS, MAPK1, RAF1, TNF, DDX58 | 1.08E-12 |
| LINC00852 | hsa04010: MAPK signaling pathway | FAS, BRAF, MAPK14, FGFR3, HRAS, IGF1R, KRAS, MAX, MAP3K11, MYC, NRAS, MAPK1, RAF1, TGFB1, TNF, VEGFA | 1.38E-14 |
| LINC00852 | hsa01522: Endocrine resistance | BAX, BCL2, BRAF, CDKN2A, MAPK14, ESR1, ESR2, HRAS, IGF1R, KRAS, NRAS, MAPK1, RAF1 | 6.46E-17 |
| LINC00852 | hsa01521: EGFR tyrosine kinase inhibitor resistance | BAX, BCL2, BRAF, FGFR3, HRAS, IGF1R, IL6R, KRAS, NRAS, MAPK1, RAF1, VEGFA | 2.25E-16 |
| LINC00863 | hsa04010: MAPK signaling pathway | FAS, BRAF, MAPK14, FGFR3, HRAS, IGF1R, KRAS, MAX, MAP3K11, MYC, NRAS, MAPK1, RAF1, TGFB1, TNF, VEGFA | 1.38E-14 |
| LINC00869 | hsa05219: Bladder cancer | BRAF, CDKN2A, FGFR3, HRAS, CXCL8, KRAS, MYC, NRAS, MAPK1, RAF1, VEGFA | 5.12E-18 |
| LINC00869 | hsa05205: Proteoglycans in cancer | FAS, BRAF, MAPK14, ESR1, HRAS, IGF1R, KRAS, MYC, NRAS, MAPK1, RAF1, TGFB1, TNF, VEGFA | 4.30E-14 |
| LINC00869 | hsa05163: Human cytomegalovirus infection | FAS, BAX, CDKN2A, MAPK14, HRAS, IL6R, CXCL8, KRAS, MYC, NRAS, MAPK1, RAF1, TNF, VEGFA | 1.57E-13 |
| LINC00869 | hsa05161: Hepatitis B | FAS, BAX, BCL2, BRAF, MAPK14, HRAS, CXCL8, KRAS, MYC, NRAS, MAPK1, RAF1, TGFB1, TNF, DDX58 | 4.09E-17 |
| LINC00869 | hsa05160: Hepatitis C | FAS, BAX, BRAF, HRAS, KRAS, LDLR, MYC, NRAS, MAPK1, RAF1, TNF, DDX58 | 1.08E-12 |
| LINC00869 | hsa04010: MAPK signaling pathway | FAS, BRAF, MAPK14, FGFR3, HRAS, IGF1R, KRAS, MAX, MAP3K11, MYC, NRAS, MAPK1, RAF1, TGFB1, TNF, VEGFA | 1.38E-14 |
| LINC00869 | hsa01522: Endocrine resistance | BAX, BCL2, BRAF, CDKN2A, MAPK14, ESR1, ESR2, HRAS, IGF1R, KRAS, NRAS, MAPK1, RAF1 | 6.46E-17 |
| LINC00869 | hsa01521: EGFR tyrosine kinase inhibitor resistance | BAX, BCL2, BRAF, FGFR3, HRAS, IGF1R, IL6R, KRAS, NRAS, MAPK1, RAF1, VEGFA | 2.25E-16 |
| LINC00884 | hsa05205: Proteoglycans in cancer | FAS, BRAF, MAPK14, ESR1, HRAS, IGF1R, KRAS, MYC, NRAS, MAPK1, RAF1, TGFB1, TNF, VEGFA | 4.30E-14 |
| LINC00884 | hsa05163: Human cytomegalovirus infection | FAS, BAX, CDKN2A, MAPK14, HRAS, IL6R, CXCL8, KRAS, MYC, NRAS, MAPK1, RAF1, TNF, VEGFA | 1.57E-13 |
| LINC00884 | hsa05161: Hepatitis B | FAS, BAX, BCL2, BRAF, MAPK14, HRAS, CXCL8, KRAS, MYC, NRAS, MAPK1, RAF1, TGFB1, TNF, DDX58 | 4.09E-17 |
| LINC00884 | hsa04010: MAPK signaling pathway | FAS, BRAF, MAPK14, FGFR3, HRAS, IGF1R, KRAS, MAX, MAP3K11, MYC, NRAS, MAPK1, RAF1, TGFB1, TNF, VEGFA | 1.38E-14 |
| LINC00884 | hsa01522: Endocrine resistance | BAX, BCL2, BRAF, CDKN2A, MAPK14, ESR1, ESR2, HRAS, IGF1R, KRAS, NRAS, MAPK1, RAF1 | 6.46E-17 |
| LINC00894 | hsa05161: Hepatitis B | FAS, BAX, BCL2, BRAF, MAPK14, HRAS, CXCL8, KRAS, MYC, NRAS, MAPK1, RAF1, TGFB1, TNF, DDX58 | 4.09E-17 |
| LINC00894 | hsa04010: MAPK signaling pathway | FAS, BRAF, MAPK14, FGFR3, HRAS, IGF1R, KRAS, MAX, MAP3K11, MYC, NRAS, MAPK1, RAF1, TGFB1, TNF, VEGFA | 1.38E-14 |
| LINC00894 | hsa01522: Endocrine resistance | BAX, BCL2, BRAF, CDKN2A, MAPK14, ESR1, ESR2, HRAS, IGF1R, KRAS, NRAS, MAPK1, RAF1 | 6.46E-17 |
| LINC00894 | hsa01521: EGFR tyrosine kinase inhibitor resistance | BAX, BCL2, BRAF, FGFR3, HRAS, IGF1R, IL6R, KRAS, NRAS, MAPK1, RAF1, VEGFA | 2.25E-16 |
| LINC00954 | hsa05219: Bladder cancer | BRAF, CDKN2A, FGFR3, HRAS, CXCL8, KRAS, MYC, NRAS, MAPK1, RAF1, VEGFA | 5.12E-18 |
| LINC00954 | hsa05205: Proteoglycans in cancer | FAS, BRAF, MAPK14, ESR1, HRAS, IGF1R, KRAS, MYC, NRAS, MAPK1, RAF1, TGFB1, TNF, VEGFA | 4.30E-14 |
| LINC00954 | hsa05163: Human cytomegalovirus infection | FAS, BAX, CDKN2A, MAPK14, HRAS, IL6R, CXCL8, KRAS, MYC, NRAS, MAPK1, RAF1, TNF, VEGFA | 1.57E-13 |
| LINC00954 | hsa05161: Hepatitis B | FAS, BAX, BCL2, BRAF, MAPK14, HRAS, CXCL8, KRAS, MYC, NRAS, MAPK1, RAF1, TGFB1, TNF, DDX58 | 4.09E-17 |
| LINC00954 | hsa05160: Hepatitis C | FAS, BAX, BRAF, HRAS, KRAS, LDLR, MYC, NRAS, MAPK1, RAF1, TNF, DDX58 | 1.08E-12 |
| LINC00954 | hsa04010: MAPK signaling pathway | FAS, BRAF, MAPK14, FGFR3, HRAS, IGF1R, KRAS, MAX, MAP3K11, MYC, NRAS, MAPK1, RAF1, TGFB1, TNF, VEGFA | 1.38E-14 |
| LINC00954 | hsa01522: Endocrine resistance | BAX, BCL2, BRAF, CDKN2A, MAPK14, ESR1, ESR2, HRAS, IGF1R, KRAS, NRAS, MAPK1, RAF1 | 6.46E-17 |
| LINC00954 | hsa01521: EGFR tyrosine kinase inhibitor resistance | BAX, BCL2, BRAF, FGFR3, HRAS, IGF1R, IL6R, KRAS, NRAS, MAPK1, RAF1, VEGFA | 2.25E-16 |
| LINC00960 | hsa05219: Bladder cancer | BRAF, CDKN2A, FGFR3, HRAS, CXCL8, KRAS, MYC, NRAS, MAPK1, RAF1, VEGFA | 5.12E-18 |
| LINC00960 | hsa04010: MAPK signaling pathway | FAS, BRAF, MAPK14, FGFR3, HRAS, IGF1R, KRAS, MAX, MAP3K11, MYC, NRAS, MAPK1, RAF1, TGFB1, TNF, VEGFA | 1.38E-14 |
| LINC00960 | hsa01521: EGFR tyrosine kinase inhibitor resistance | BAX, BCL2, BRAF, FGFR3, HRAS, IGF1R, IL6R, KRAS, NRAS, MAPK1, RAF1, VEGFA | 2.25E-16 |
| LINC00996 | hsa05219: Bladder cancer | BRAF, CDKN2A, FGFR3, HRAS, CXCL8, KRAS, MYC, NRAS, MAPK1, RAF1, VEGFA | 5.12E-18 |
| LINC00996 | hsa05205: Proteoglycans in cancer | FAS, BRAF, MAPK14, ESR1, HRAS, IGF1R, KRAS, MYC, NRAS, MAPK1, RAF1, TGFB1, TNF, VEGFA | 4.30E-14 |
| LINC00996 | hsa05163: Human cytomegalovirus infection | FAS, BAX, CDKN2A, MAPK14, HRAS, IL6R, CXCL8, KRAS, MYC, NRAS, MAPK1, RAF1, TNF, VEGFA | 1.57E-13 |
| LINC00996 | hsa04010: MAPK signaling pathway | FAS, BRAF, MAPK14, FGFR3, HRAS, IGF1R, KRAS, MAX, MAP3K11, MYC, NRAS, MAPK1, RAF1, TGFB1, TNF, VEGFA | 1.38E-14 |
| LINC00996 | hsa01521: EGFR tyrosine kinase inhibitor resistance | BAX, BCL2, BRAF, FGFR3, HRAS, IGF1R, IL6R, KRAS, NRAS, MAPK1, RAF1, VEGFA | 2.25E-16 |
| LINC00998 | hsa05205: Proteoglycans in cancer | FAS, BRAF, MAPK14, ESR1, HRAS, IGF1R, KRAS, MYC, NRAS, MAPK1, RAF1, TGFB1, TNF, VEGFA | 4.30E-14 |
| LINC00998 | hsa05163: Human cytomegalovirus infection | FAS, BAX, CDKN2A, MAPK14, HRAS, IL6R, CXCL8, KRAS, MYC, NRAS, MAPK1, RAF1, TNF, VEGFA | 1.57E-13 |
| LINC00998 | hsa05161: Hepatitis B | FAS, BAX, BCL2, BRAF, MAPK14, HRAS, CXCL8, KRAS, MYC, NRAS, MAPK1, RAF1, TGFB1, TNF, DDX58 | 4.09E-17 |
| LINC00998 | hsa05160: Hepatitis C | FAS, BAX, BRAF, HRAS, KRAS, LDLR, MYC, NRAS, MAPK1, RAF1, TNF, DDX58 | 1.08E-12 |
| LINC00998 | hsa04010: MAPK signaling pathway | FAS, BRAF, MAPK14, FGFR3, HRAS, IGF1R, KRAS, MAX, MAP3K11, MYC, NRAS, MAPK1, RAF1, TGFB1, TNF, VEGFA | 1.38E-14 |
| LINC01089 | hsa04010: MAPK signaling pathway | FAS, BRAF, MAPK14, FGFR3, HRAS, IGF1R, KRAS, MAX, MAP3K11, MYC, NRAS, MAPK1, RAF1, TGFB1, TNF, VEGFA | 1.38E-14 |
| LINC01118 | hsa05163: Human cytomegalovirus infection | FAS, BAX, CDKN2A, MAPK14, HRAS, IL6R, CXCL8, KRAS, MYC, NRAS, MAPK1, RAF1, TNF, VEGFA | 1.57E-13 |
| LINC01118 | hsa04010: MAPK signaling pathway | FAS, BRAF, MAPK14, FGFR3, HRAS, IGF1R, KRAS, MAX, MAP3K11, MYC, NRAS, MAPK1, RAF1, TGFB1, TNF, VEGFA | 1.38E-14 |
| LINC01118 | hsa01521: EGFR tyrosine kinase inhibitor resistance | BAX, BCL2, BRAF, FGFR3, HRAS, IGF1R, IL6R, KRAS, NRAS, MAPK1, RAF1, VEGFA | 2.25E-16 |
| LINC01125 | hsa05163: Human cytomegalovirus infection | FAS, BAX, CDKN2A, MAPK14, HRAS, IL6R, CXCL8, KRAS, MYC, NRAS, MAPK1, RAF1, TNF, VEGFA | 1.57E-13 |
| LINC01125 | hsa05161: Hepatitis B | FAS, BAX, BCL2, BRAF, MAPK14, HRAS, CXCL8, KRAS, MYC, NRAS, MAPK1, RAF1, TGFB1, TNF, DDX58 | 4.09E-17 |
| LINC01125 | hsa05160: Hepatitis C | FAS, BAX, BRAF, HRAS, KRAS, LDLR, MYC, NRAS, MAPK1, RAF1, TNF, DDX58 | 1.08E-12 |
| LINC01125 | hsa01522: Endocrine resistance | BAX, BCL2, BRAF, CDKN2A, MAPK14, ESR1, ESR2, HRAS, IGF1R, KRAS, NRAS, MAPK1, RAF1 | 6.46E-17 |
| LINC01125 | hsa01521: EGFR tyrosine kinase inhibitor resistance | BAX, BCL2, BRAF, FGFR3, HRAS, IGF1R, IL6R, KRAS, NRAS, MAPK1, RAF1, VEGFA | 2.25E-16 |
| LINC01126 | hsa05219: Bladder cancer | BRAF, CDKN2A, FGFR3, HRAS, CXCL8, KRAS, MYC, NRAS, MAPK1, RAF1, VEGFA | 5.12E-18 |
| LINC01126 | hsa05205: Proteoglycans in cancer | FAS, BRAF, MAPK14, ESR1, HRAS, IGF1R, KRAS, MYC, NRAS, MAPK1, RAF1, TGFB1, TNF, VEGFA | 4.30E-14 |
| LINC01126 | hsa05163: Human cytomegalovirus infection | FAS, BAX, CDKN2A, MAPK14, HRAS, IL6R, CXCL8, KRAS, MYC, NRAS, MAPK1, RAF1, TNF, VEGFA | 1.57E-13 |
| LINC01126 | hsa05161: Hepatitis B | FAS, BAX, BCL2, BRAF, MAPK14, HRAS, CXCL8, KRAS, MYC, NRAS, MAPK1, RAF1, TGFB1, TNF, DDX58 | 4.09E-17 |
| LINC01126 | hsa05160: Hepatitis C | FAS, BAX, BRAF, HRAS, KRAS, LDLR, MYC, NRAS, MAPK1, RAF1, TNF, DDX58 | 1.08E-12 |
| LINC01126 | hsa04010: MAPK signaling pathway | FAS, BRAF, MAPK14, FGFR3, HRAS, IGF1R, KRAS, MAX, MAP3K11, MYC, NRAS, MAPK1, RAF1, TGFB1, TNF, VEGFA | 1.38E-14 |
| LINC01126 | hsa01522: Endocrine resistance | BAX, BCL2, BRAF, CDKN2A, MAPK14, ESR1, ESR2, HRAS, IGF1R, KRAS, NRAS, MAPK1, RAF1 | 6.46E-17 |
| LINC01126 | hsa01521: EGFR tyrosine kinase inhibitor resistance | BAX, BCL2, BRAF, FGFR3, HRAS, IGF1R, IL6R, KRAS, NRAS, MAPK1, RAF1, VEGFA | 2.25E-16 |
| LINC01128 | hsa05161: Hepatitis B | FAS, BAX, BCL2, BRAF, MAPK14, HRAS, CXCL8, KRAS, MYC, NRAS, MAPK1, RAF1, TGFB1, TNF, DDX58 | 4.09E-17 |
| LINC01128 | hsa01522: Endocrine resistance | BAX, BCL2, BRAF, CDKN2A, MAPK14, ESR1, ESR2, HRAS, IGF1R, KRAS, NRAS, MAPK1, RAF1 | 6.46E-17 |
| LINC01128 | hsa01521: EGFR tyrosine kinase inhibitor resistance | BAX, BCL2, BRAF, FGFR3, HRAS, IGF1R, IL6R, KRAS, NRAS, MAPK1, RAF1, VEGFA | 2.25E-16 |
| LINC01134 | hsa05205: Proteoglycans in cancer | FAS, BRAF, MAPK14, ESR1, HRAS, IGF1R, KRAS, MYC, NRAS, MAPK1, RAF1, TGFB1, TNF, VEGFA | 4.30E-14 |
| LINC01134 | hsa05163: Human cytomegalovirus infection | FAS, BAX, CDKN2A, MAPK14, HRAS, IL6R, CXCL8, KRAS, MYC, NRAS, MAPK1, RAF1, TNF, VEGFA | 1.57E-13 |
| LINC01134 | hsa05161: Hepatitis B | FAS, BAX, BCL2, BRAF, MAPK14, HRAS, CXCL8, KRAS, MYC, NRAS, MAPK1, RAF1, TGFB1, TNF, DDX58 | 4.09E-17 |
| LINC01134 | hsa04010: MAPK signaling pathway | FAS, BRAF, MAPK14, FGFR3, HRAS, IGF1R, KRAS, MAX, MAP3K11, MYC, NRAS, MAPK1, RAF1, TGFB1, TNF, VEGFA | 1.38E-14 |
| LINC01134 | hsa01522: Endocrine resistance | BAX, BCL2, BRAF, CDKN2A, MAPK14, ESR1, ESR2, HRAS, IGF1R, KRAS, NRAS, MAPK1, RAF1 | 6.46E-17 |
| LINC01144 | hsa05163: Human cytomegalovirus infection | FAS, BAX, CDKN2A, MAPK14, HRAS, IL6R, CXCL8, KRAS, MYC, NRAS, MAPK1, RAF1, TNF, VEGFA | 1.57E-13 |
| LINC01144 | hsa05161: Hepatitis B | FAS, BAX, BCL2, BRAF, MAPK14, HRAS, CXCL8, KRAS, MYC, NRAS, MAPK1, RAF1, TGFB1, TNF, DDX58 | 4.09E-17 |
| LINC01144 | hsa05160: Hepatitis C | FAS, BAX, BRAF, HRAS, KRAS, LDLR, MYC, NRAS, MAPK1, RAF1, TNF, DDX58 | 1.08E-12 |
| LINC01144 | hsa01522: Endocrine resistance | BAX, BCL2, BRAF, CDKN2A, MAPK14, ESR1, ESR2, HRAS, IGF1R, KRAS, NRAS, MAPK1, RAF1 | 6.46E-17 |
| LINC01144 | hsa01521: EGFR tyrosine kinase inhibitor resistance | BAX, BCL2, BRAF, FGFR3, HRAS, IGF1R, IL6R, KRAS, NRAS, MAPK1, RAF1, VEGFA | 2.25E-16 |
| LINC01146 | hsa05163: Human cytomegalovirus infection | FAS, BAX, CDKN2A, MAPK14, HRAS, IL6R, CXCL8, KRAS, MYC, NRAS, MAPK1, RAF1, TNF, VEGFA | 1.57E-13 |
| LINC01146 | hsa05161: Hepatitis B | FAS, BAX, BCL2, BRAF, MAPK14, HRAS, CXCL8, KRAS, MYC, NRAS, MAPK1, RAF1, TGFB1, TNF, DDX58 | 4.09E-17 |
| LINC01146 | hsa05160: Hepatitis C | FAS, BAX, BRAF, HRAS, KRAS, LDLR, MYC, NRAS, MAPK1, RAF1, TNF, DDX58 | 1.08E-12 |
| LINC01146 | hsa01522: Endocrine resistance | BAX, BCL2, BRAF, CDKN2A, MAPK14, ESR1, ESR2, HRAS, IGF1R, KRAS, NRAS, MAPK1, RAF1 | 6.46E-17 |
| LINC01146 | hsa01521: EGFR tyrosine kinase inhibitor resistance | BAX, BCL2, BRAF, FGFR3, HRAS, IGF1R, IL6R, KRAS, NRAS, MAPK1, RAF1, VEGFA | 2.25E-16 |
| LINC01366 | hsa05219: Bladder cancer | BRAF, CDKN2A, FGFR3, HRAS, CXCL8, KRAS, MYC, NRAS, MAPK1, RAF1, VEGFA | 5.12E-18 |
| LINC01366 | hsa05205: Proteoglycans in cancer | FAS, BRAF, MAPK14, ESR1, HRAS, IGF1R, KRAS, MYC, NRAS, MAPK1, RAF1, TGFB1, TNF, VEGFA | 4.30E-14 |
| LINC01366 | hsa05163: Human cytomegalovirus infection | FAS, BAX, CDKN2A, MAPK14, HRAS, IL6R, CXCL8, KRAS, MYC, NRAS, MAPK1, RAF1, TNF, VEGFA | 1.57E-13 |
| LINC01366 | hsa04010: MAPK signaling pathway | FAS, BRAF, MAPK14, FGFR3, HRAS, IGF1R, KRAS, MAX, MAP3K11, MYC, NRAS, MAPK1, RAF1, TGFB1, TNF, VEGFA | 1.38E-14 |
| LINC01366 | hsa01521: EGFR tyrosine kinase inhibitor resistance | BAX, BCL2, BRAF, FGFR3, HRAS, IGF1R, IL6R, KRAS, NRAS, MAPK1, RAF1, VEGFA | 2.25E-16 |
| LINC01547 | hsa05205: Proteoglycans in cancer | FAS, BRAF, MAPK14, ESR1, HRAS, IGF1R, KRAS, MYC, NRAS, MAPK1, RAF1, TGFB1, TNF, VEGFA | 4.30E-14 |
| LINC01547 | hsa05163: Human cytomegalovirus infection | FAS, BAX, CDKN2A, MAPK14, HRAS, IL6R, CXCL8, KRAS, MYC, NRAS, MAPK1, RAF1, TNF, VEGFA | 1.57E-13 |
| LINC01547 | hsa05161: Hepatitis B | FAS, BAX, BCL2, BRAF, MAPK14, HRAS, CXCL8, KRAS, MYC, NRAS, MAPK1, RAF1, TGFB1, TNF, DDX58 | 4.09E-17 |
| LINC01547 | hsa04010: MAPK signaling pathway | FAS, BRAF, MAPK14, FGFR3, HRAS, IGF1R, KRAS, MAX, MAP3K11, MYC, NRAS, MAPK1, RAF1, TGFB1, TNF, VEGFA | 1.38E-14 |
| LINC01547 | hsa01522: Endocrine resistance | BAX, BCL2, BRAF, CDKN2A, MAPK14, ESR1, ESR2, HRAS, IGF1R, KRAS, NRAS, MAPK1, RAF1 | 6.46E-17 |
| LINC01579 | hsa05219: Bladder cancer | BRAF, CDKN2A, FGFR3, HRAS, CXCL8, KRAS, MYC, NRAS, MAPK1, RAF1, VEGFA | 5.12E-18 |
| LINC01579 | hsa05205: Proteoglycans in cancer | FAS, BRAF, MAPK14, ESR1, HRAS, IGF1R, KRAS, MYC, NRAS, MAPK1, RAF1, TGFB1, TNF, VEGFA | 4.30E-14 |
| LINC01579 | hsa05163: Human cytomegalovirus infection | FAS, BAX, CDKN2A, MAPK14, HRAS, IL6R, CXCL8, KRAS, MYC, NRAS, MAPK1, RAF1, TNF, VEGFA | 1.57E-13 |
| LINC01579 | hsa05161: Hepatitis B | FAS, BAX, BCL2, BRAF, MAPK14, HRAS, CXCL8, KRAS, MYC, NRAS, MAPK1, RAF1, TGFB1, TNF, DDX58 | 4.09E-17 |
| LINC01579 | hsa05160: Hepatitis C | FAS, BAX, BRAF, HRAS, KRAS, LDLR, MYC, NRAS, MAPK1, RAF1, TNF, DDX58 | 1.08E-12 |
| LINC01579 | hsa04010: MAPK signaling pathway | FAS, BRAF, MAPK14, FGFR3, HRAS, IGF1R, KRAS, MAX, MAP3K11, MYC, NRAS, MAPK1, RAF1, TGFB1, TNF, VEGFA | 1.38E-14 |
| LINC01579 | hsa01522: Endocrine resistance | BAX, BCL2, BRAF, CDKN2A, MAPK14, ESR1, ESR2, HRAS, IGF1R, KRAS, NRAS, MAPK1, RAF1 | 6.46E-17 |
| LINC01579 | hsa01521: EGFR tyrosine kinase inhibitor resistance | BAX, BCL2, BRAF, FGFR3, HRAS, IGF1R, IL6R, KRAS, NRAS, MAPK1, RAF1, VEGFA | 2.25E-16 |
| MALAT1 | hsa05219: Bladder cancer | BRAF, CDKN2A, FGFR3, HRAS, CXCL8, KRAS, MYC, NRAS, MAPK1, RAF1, VEGFA | 5.12E-18 |
| MALAT1 | hsa05205: Proteoglycans in cancer | FAS, BRAF, MAPK14, ESR1, HRAS, IGF1R, KRAS, MYC, NRAS, MAPK1, RAF1, TGFB1, TNF, VEGFA | 4.30E-14 |
| MALAT1 | hsa05161: Hepatitis B | FAS, BAX, BCL2, BRAF, MAPK14, HRAS, CXCL8, KRAS, MYC, NRAS, MAPK1, RAF1, TGFB1, TNF, DDX58 | 4.09E-17 |
| MALAT1 | hsa05160: Hepatitis C | FAS, BAX, BRAF, HRAS, KRAS, LDLR, MYC, NRAS, MAPK1, RAF1, TNF, DDX58 | 1.08E-12 |
| MALAT1 | hsa04010: MAPK signaling pathway | FAS, BRAF, MAPK14, FGFR3, HRAS, IGF1R, KRAS, MAX, MAP3K11, MYC, NRAS, MAPK1, RAF1, TGFB1, TNF, VEGFA | 1.38E-14 |
| MALAT1 | hsa01522: Endocrine resistance | BAX, BCL2, BRAF, CDKN2A, MAPK14, ESR1, ESR2, HRAS, IGF1R, KRAS, NRAS, MAPK1, RAF1 | 6.46E-17 |
| MALAT1 | hsa01521: EGFR tyrosine kinase inhibitor resistance | BAX, BCL2, BRAF, FGFR3, HRAS, IGF1R, IL6R, KRAS, NRAS, MAPK1, RAF1, VEGFA | 2.25E-16 |
| MBNL1-AS1 | hsa05205: Proteoglycans in cancer | FAS, BRAF, MAPK14, ESR1, HRAS, IGF1R, KRAS, MYC, NRAS, MAPK1, RAF1, TGFB1, TNF, VEGFA | 4.30E-14 |
| MBNL1-AS1 | hsa05163: Human cytomegalovirus infection | FAS, BAX, CDKN2A, MAPK14, HRAS, IL6R, CXCL8, KRAS, MYC, NRAS, MAPK1, RAF1, TNF, VEGFA | 1.57E-13 |
| MBNL1-AS1 | hsa05161: Hepatitis B | FAS, BAX, BCL2, BRAF, MAPK14, HRAS, CXCL8, KRAS, MYC, NRAS, MAPK1, RAF1, TGFB1, TNF, DDX58 | 4.09E-17 |
| MBNL1-AS1 | hsa05160: Hepatitis C | FAS, BAX, BRAF, HRAS, KRAS, LDLR, MYC, NRAS, MAPK1, RAF1, TNF, DDX58 | 1.08E-12 |
| MBNL1-AS1 | hsa04010: MAPK signaling pathway | FAS, BRAF, MAPK14, FGFR3, HRAS, IGF1R, KRAS, MAX, MAP3K11, MYC, NRAS, MAPK1, RAF1, TGFB1, TNF, VEGFA | 1.38E-14 |
| MBNL1-AS1 | hsa01522: Endocrine resistance | BAX, BCL2, BRAF, CDKN2A, MAPK14, ESR1, ESR2, HRAS, IGF1R, KRAS, NRAS, MAPK1, RAF1 | 6.46E-17 |
| MCM3AP-AS1 | hsa05219: Bladder cancer | BRAF, CDKN2A, FGFR3, HRAS, CXCL8, KRAS, MYC, NRAS, MAPK1, RAF1, VEGFA | 5.12E-18 |
| MCM3AP-AS1 | hsa05205: Proteoglycans in cancer | FAS, BRAF, MAPK14, ESR1, HRAS, IGF1R, KRAS, MYC, NRAS, MAPK1, RAF1, TGFB1, TNF, VEGFA | 4.30E-14 |
| MCM3AP-AS1 | hsa05163: Human cytomegalovirus infection | FAS, BAX, CDKN2A, MAPK14, HRAS, IL6R, CXCL8, KRAS, MYC, NRAS, MAPK1, RAF1, TNF, VEGFA | 1.57E-13 |
| MCM3AP-AS1 | hsa05161: Hepatitis B | FAS, BAX, BCL2, BRAF, MAPK14, HRAS, CXCL8, KRAS, MYC, NRAS, MAPK1, RAF1, TGFB1, TNF, DDX58 | 4.09E-17 |
| MCM3AP-AS1 | hsa05160: Hepatitis C | FAS, BAX, BRAF, HRAS, KRAS, LDLR, MYC, NRAS, MAPK1, RAF1, TNF, DDX58 | 1.08E-12 |
| MCM3AP-AS1 | hsa04010: MAPK signaling pathway | FAS, BRAF, MAPK14, FGFR3, HRAS, IGF1R, KRAS, MAX, MAP3K11, MYC, NRAS, MAPK1, RAF1, TGFB1, TNF, VEGFA | 1.38E-14 |
| MCM3AP-AS1 | hsa01522: Endocrine resistance | BAX, BCL2, BRAF, CDKN2A, MAPK14, ESR1, ESR2, HRAS, IGF1R, KRAS, NRAS, MAPK1, RAF1 | 6.46E-17 |
| MCM3AP-AS1 | hsa01521: EGFR tyrosine kinase inhibitor resistance | BAX, BCL2, BRAF, FGFR3, HRAS, IGF1R, IL6R, KRAS, NRAS, MAPK1, RAF1, VEGFA | 2.25E-16 |
| MIR17HG | hsa05219: Bladder cancer | BRAF, CDKN2A, FGFR3, HRAS, CXCL8, KRAS, MYC, NRAS, MAPK1, RAF1, VEGFA | 5.12E-18 |
| MIR17HG | hsa05205: Proteoglycans in cancer | FAS, BRAF, MAPK14, ESR1, HRAS, IGF1R, KRAS, MYC, NRAS, MAPK1, RAF1, TGFB1, TNF, VEGFA | 4.30E-14 |
| MIR17HG | hsa05163: Human cytomegalovirus infection | FAS, BAX, CDKN2A, MAPK14, HRAS, IL6R, CXCL8, KRAS, MYC, NRAS, MAPK1, RAF1, TNF, VEGFA | 1.57E-13 |
| MIR17HG | hsa05161: Hepatitis B | FAS, BAX, BCL2, BRAF, MAPK14, HRAS, CXCL8, KRAS, MYC, NRAS, MAPK1, RAF1, TGFB1, TNF, DDX58 | 4.09E-17 |
| MIR17HG | hsa05160: Hepatitis C | FAS, BAX, BRAF, HRAS, KRAS, LDLR, MYC, NRAS, MAPK1, RAF1, TNF, DDX58 | 1.08E-12 |
| MIR17HG | hsa04010: MAPK signaling pathway | FAS, BRAF, MAPK14, FGFR3, HRAS, IGF1R, KRAS, MAX, MAP3K11, MYC, NRAS, MAPK1, RAF1, TGFB1, TNF, VEGFA | 1.38E-14 |
| MIR17HG | hsa01522: Endocrine resistance | BAX, BCL2, BRAF, CDKN2A, MAPK14, ESR1, ESR2, HRAS, IGF1R, KRAS, NRAS, MAPK1, RAF1 | 6.46E-17 |
| MIR17HG | hsa01521: EGFR tyrosine kinase inhibitor resistance | BAX, BCL2, BRAF, FGFR3, HRAS, IGF1R, IL6R, KRAS, NRAS, MAPK1, RAF1, VEGFA | 2.25E-16 |
| MZF1-AS1 | hsa05219: Bladder cancer | BRAF, CDKN2A, FGFR3, HRAS, CXCL8, KRAS, MYC, NRAS, MAPK1, RAF1, VEGFA | 5.12E-18 |
| MZF1-AS1 | hsa05205: Proteoglycans in cancer | FAS, BRAF, MAPK14, ESR1, HRAS, IGF1R, KRAS, MYC, NRAS, MAPK1, RAF1, TGFB1, TNF, VEGFA | 4.30E-14 |
| MZF1-AS1 | hsa05163: Human cytomegalovirus infection | FAS, BAX, CDKN2A, MAPK14, HRAS, IL6R, CXCL8, KRAS, MYC, NRAS, MAPK1, RAF1, TNF, VEGFA | 1.57E-13 |
| MZF1-AS1 | hsa05161: Hepatitis B | FAS, BAX, BCL2, BRAF, MAPK14, HRAS, CXCL8, KRAS, MYC, NRAS, MAPK1, RAF1, TGFB1, TNF, DDX58 | 4.09E-17 |
| MZF1-AS1 | hsa05160: Hepatitis C | FAS, BAX, BRAF, HRAS, KRAS, LDLR, MYC, NRAS, MAPK1, RAF1, TNF, DDX58 | 1.08E-12 |
| MZF1-AS1 | hsa04010: MAPK signaling pathway | FAS, BRAF, MAPK14, FGFR3, HRAS, IGF1R, KRAS, MAX, MAP3K11, MYC, NRAS, MAPK1, RAF1, TGFB1, TNF, VEGFA | 1.38E-14 |
| MZF1-AS1 | hsa01522: Endocrine resistance | BAX, BCL2, BRAF, CDKN2A, MAPK14, ESR1, ESR2, HRAS, IGF1R, KRAS, NRAS, MAPK1, RAF1 | 6.46E-17 |
| MZF1-AS1 | hsa01521: EGFR tyrosine kinase inhibitor resistance | BAX, BCL2, BRAF, FGFR3, HRAS, IGF1R, IL6R, KRAS, NRAS, MAPK1, RAF1, VEGFA | 2.25E-16 |
| NCAM1-AS1 | hsa05161: Hepatitis B | FAS, BAX, BCL2, BRAF, MAPK14, HRAS, CXCL8, KRAS, MYC, NRAS, MAPK1, RAF1, TGFB1, TNF, DDX58 | 4.09E-17 |
| NCAM1-AS1 | hsa01522: Endocrine resistance | BAX, BCL2, BRAF, CDKN2A, MAPK14, ESR1, ESR2, HRAS, IGF1R, KRAS, NRAS, MAPK1, RAF1 | 6.46E-17 |
| NCAM1-AS1 | hsa01521: EGFR tyrosine kinase inhibitor resistance | BAX, BCL2, BRAF, FGFR3, HRAS, IGF1R, IL6R, KRAS, NRAS, MAPK1, RAF1, VEGFA | 2.25E-16 |
| NCBP2-AS2 | hsa05219: Bladder cancer | BRAF, CDKN2A, FGFR3, HRAS, CXCL8, KRAS, MYC, NRAS, MAPK1, RAF1, VEGFA | 5.12E-18 |
| NCBP2-AS2 | hsa05205: Proteoglycans in cancer | FAS, BRAF, MAPK14, ESR1, HRAS, IGF1R, KRAS, MYC, NRAS, MAPK1, RAF1, TGFB1, TNF, VEGFA | 4.30E-14 |
| NCBP2-AS2 | hsa05163: Human cytomegalovirus infection | FAS, BAX, CDKN2A, MAPK14, HRAS, IL6R, CXCL8, KRAS, MYC, NRAS, MAPK1, RAF1, TNF, VEGFA | 1.57E-13 |
| NCBP2-AS2 | hsa05161: Hepatitis B | FAS, BAX, BCL2, BRAF, MAPK14, HRAS, CXCL8, KRAS, MYC, NRAS, MAPK1, RAF1, TGFB1, TNF, DDX58 | 4.09E-17 |
| NCBP2-AS2 | hsa05160: Hepatitis C | FAS, BAX, BRAF, HRAS, KRAS, LDLR, MYC, NRAS, MAPK1, RAF1, TNF, DDX58 | 1.08E-12 |
| NCBP2-AS2 | hsa04010: MAPK signaling pathway | FAS, BRAF, MAPK14, FGFR3, HRAS, IGF1R, KRAS, MAX, MAP3K11, MYC, NRAS, MAPK1, RAF1, TGFB1, TNF, VEGFA | 1.38E-14 |
| NCBP2-AS2 | hsa01522: Endocrine resistance | BAX, BCL2, BRAF, CDKN2A, MAPK14, ESR1, ESR2, HRAS, IGF1R, KRAS, NRAS, MAPK1, RAF1 | 6.46E-17 |
| NCBP2-AS2 | hsa01521: EGFR tyrosine kinase inhibitor resistance | BAX, BCL2, BRAF, FGFR3, HRAS, IGF1R, IL6R, KRAS, NRAS, MAPK1, RAF1, VEGFA | 2.25E-16 |
| NDUFA6-AS1 | hsa05163: Human cytomegalovirus infection | FAS, BAX, CDKN2A, MAPK14, HRAS, IL6R, CXCL8, KRAS, MYC, NRAS, MAPK1, RAF1, TNF, VEGFA | 1.57E-13 |
| NDUFA6-AS1 | hsa05161: Hepatitis B | FAS, BAX, BCL2, BRAF, MAPK14, HRAS, CXCL8, KRAS, MYC, NRAS, MAPK1, RAF1, TGFB1, TNF, DDX58 | 4.09E-17 |
| NDUFA6-AS1 | hsa05160: Hepatitis C | FAS, BAX, BRAF, HRAS, KRAS, LDLR, MYC, NRAS, MAPK1, RAF1, TNF, DDX58 | 1.08E-12 |
| NDUFA6-AS1 | hsa01522: Endocrine resistance | BAX, BCL2, BRAF, CDKN2A, MAPK14, ESR1, ESR2, HRAS, IGF1R, KRAS, NRAS, MAPK1, RAF1 | 6.46E-17 |
| NDUFA6-AS1 | hsa01521: EGFR tyrosine kinase inhibitor resistance | BAX, BCL2, BRAF, FGFR3, HRAS, IGF1R, IL6R, KRAS, NRAS, MAPK1, RAF1, VEGFA | 2.25E-16 |
| NFYC-AS1 | hsa05219: Bladder cancer | BRAF, CDKN2A, FGFR3, HRAS, CXCL8, KRAS, MYC, NRAS, MAPK1, RAF1, VEGFA | 5.12E-18 |
| NFYC-AS1 | hsa05205: Proteoglycans in cancer | FAS, BRAF, MAPK14, ESR1, HRAS, IGF1R, KRAS, MYC, NRAS, MAPK1, RAF1, TGFB1, TNF, VEGFA | 4.30E-14 |
| NFYC-AS1 | hsa05163: Human cytomegalovirus infection | FAS, BAX, CDKN2A, MAPK14, HRAS, IL6R, CXCL8, KRAS, MYC, NRAS, MAPK1, RAF1, TNF, VEGFA | 1.57E-13 |
| NFYC-AS1 | hsa05161: Hepatitis B | FAS, BAX, BCL2, BRAF, MAPK14, HRAS, CXCL8, KRAS, MYC, NRAS, MAPK1, RAF1, TGFB1, TNF, DDX58 | 4.09E-17 |
| NFYC-AS1 | hsa05160: Hepatitis C | FAS, BAX, BRAF, HRAS, KRAS, LDLR, MYC, NRAS, MAPK1, RAF1, TNF, DDX58 | 1.08E-12 |
| NFYC-AS1 | hsa04010: MAPK signaling pathway | FAS, BRAF, MAPK14, FGFR3, HRAS, IGF1R, KRAS, MAX, MAP3K11, MYC, NRAS, MAPK1, RAF1, TGFB1, TNF, VEGFA | 1.38E-14 |
| NFYC-AS1 | hsa01522: Endocrine resistance | BAX, BCL2, BRAF, CDKN2A, MAPK14, ESR1, ESR2, HRAS, IGF1R, KRAS, NRAS, MAPK1, RAF1 | 6.46E-17 |
| NFYC-AS1 | hsa01521: EGFR tyrosine kinase inhibitor resistance | BAX, BCL2, BRAF, FGFR3, HRAS, IGF1R, IL6R, KRAS, NRAS, MAPK1, RAF1, VEGFA | 2.25E-16 |
| NIFK-AS1 | hsa05219: Bladder cancer | BRAF, CDKN2A, FGFR3, HRAS, CXCL8, KRAS, MYC, NRAS, MAPK1, RAF1, VEGFA | 5.12E-18 |
| NIFK-AS1 | hsa05205: Proteoglycans in cancer | FAS, BRAF, MAPK14, ESR1, HRAS, IGF1R, KRAS, MYC, NRAS, MAPK1, RAF1, TGFB1, TNF, VEGFA | 4.30E-14 |
| NIFK-AS1 | hsa05163: Human cytomegalovirus infection | FAS, BAX, CDKN2A, MAPK14, HRAS, IL6R, CXCL8, KRAS, MYC, NRAS, MAPK1, RAF1, TNF, VEGFA | 1.57E-13 |
| NIFK-AS1 | hsa05161: Hepatitis B | FAS, BAX, BCL2, BRAF, MAPK14, HRAS, CXCL8, KRAS, MYC, NRAS, MAPK1, RAF1, TGFB1, TNF, DDX58 | 4.09E-17 |
| NIFK-AS1 | hsa05160: Hepatitis C | FAS, BAX, BRAF, HRAS, KRAS, LDLR, MYC, NRAS, MAPK1, RAF1, TNF, DDX58 | 1.08E-12 |
| NIFK-AS1 | hsa04010: MAPK signaling pathway | FAS, BRAF, MAPK14, FGFR3, HRAS, IGF1R, KRAS, MAX, MAP3K11, MYC, NRAS, MAPK1, RAF1, TGFB1, TNF, VEGFA | 1.38E-14 |
| NIFK-AS1 | hsa01522: Endocrine resistance | BAX, BCL2, BRAF, CDKN2A, MAPK14, ESR1, ESR2, HRAS, IGF1R, KRAS, NRAS, MAPK1, RAF1 | 6.46E-17 |
| NIFK-AS1 | hsa01521: EGFR tyrosine kinase inhibitor resistance | BAX, BCL2, BRAF, FGFR3, HRAS, IGF1R, IL6R, KRAS, NRAS, MAPK1, RAF1, VEGFA | 2.25E-16 |
| OIP5-AS1 | hsa05219: Bladder cancer | BRAF, CDKN2A, FGFR3, HRAS, CXCL8, KRAS, MYC, NRAS, MAPK1, RAF1, VEGFA | 5.12E-18 |
| OIP5-AS1 | hsa05205: Proteoglycans in cancer | FAS, BRAF, MAPK14, ESR1, HRAS, IGF1R, KRAS, MYC, NRAS, MAPK1, RAF1, TGFB1, TNF, VEGFA | 4.30E-14 |
| OIP5-AS1 | hsa05163: Human cytomegalovirus infection | FAS, BAX, CDKN2A, MAPK14, HRAS, IL6R, CXCL8, KRAS, MYC, NRAS, MAPK1, RAF1, TNF, VEGFA | 1.57E-13 |
| OIP5-AS1 | hsa05161: Hepatitis B | FAS, BAX, BCL2, BRAF, MAPK14, HRAS, CXCL8, KRAS, MYC, NRAS, MAPK1, RAF1, TGFB1, TNF, DDX58 | 4.09E-17 |
| OIP5-AS1 | hsa05160: Hepatitis C | FAS, BAX, BRAF, HRAS, KRAS, LDLR, MYC, NRAS, MAPK1, RAF1, TNF, DDX58 | 1.08E-12 |
| OIP5-AS1 | hsa04010: MAPK signaling pathway | FAS, BRAF, MAPK14, FGFR3, HRAS, IGF1R, KRAS, MAX, MAP3K11, MYC, NRAS, MAPK1, RAF1, TGFB1, TNF, VEGFA | 1.38E-14 |
| OIP5-AS1 | hsa01522: Endocrine resistance | BAX, BCL2, BRAF, CDKN2A, MAPK14, ESR1, ESR2, HRAS, IGF1R, KRAS, NRAS, MAPK1, RAF1 | 6.46E-17 |
| OIP5-AS1 | hsa01521: EGFR tyrosine kinase inhibitor resistance | BAX, BCL2, BRAF, FGFR3, HRAS, IGF1R, IL6R, KRAS, NRAS, MAPK1, RAF1, VEGFA | 2.25E-16 |
| PARD6G-AS1 | hsa05205: Proteoglycans in cancer | FAS, BRAF, MAPK14, ESR1, HRAS, IGF1R, KRAS, MYC, NRAS, MAPK1, RAF1, TGFB1, TNF, VEGFA | 4.30E-14 |
| PARD6G-AS1 | hsa05163: Human cytomegalovirus infection | FAS, BAX, CDKN2A, MAPK14, HRAS, IL6R, CXCL8, KRAS, MYC, NRAS, MAPK1, RAF1, TNF, VEGFA | 1.57E-13 |
| PARD6G-AS1 | hsa05161: Hepatitis B | FAS, BAX, BCL2, BRAF, MAPK14, HRAS, CXCL8, KRAS, MYC, NRAS, MAPK1, RAF1, TGFB1, TNF, DDX58 | 4.09E-17 |
| PARD6G-AS1 | hsa05160: Hepatitis C | FAS, BAX, BRAF, HRAS, KRAS, LDLR, MYC, NRAS, MAPK1, RAF1, TNF, DDX58 | 1.08E-12 |
| PARD6G-AS1 | hsa04010: MAPK signaling pathway | FAS, BRAF, MAPK14, FGFR3, HRAS, IGF1R, KRAS, MAX, MAP3K11, MYC, NRAS, MAPK1, RAF1, TGFB1, TNF, VEGFA | 1.38E-14 |
| PAXIP1-AS2 | hsa05219: Bladder cancer | BRAF, CDKN2A, FGFR3, HRAS, CXCL8, KRAS, MYC, NRAS, MAPK1, RAF1, VEGFA | 5.12E-18 |
| PAXIP1-AS2 | hsa05205: Proteoglycans in cancer | FAS, BRAF, MAPK14, ESR1, HRAS, IGF1R, KRAS, MYC, NRAS, MAPK1, RAF1, TGFB1, TNF, VEGFA | 4.30E-14 |
| PAXIP1-AS2 | hsa05163: Human cytomegalovirus infection | FAS, BAX, CDKN2A, MAPK14, HRAS, IL6R, CXCL8, KRAS, MYC, NRAS, MAPK1, RAF1, TNF, VEGFA | 1.57E-13 |
| PAXIP1-AS2 | hsa05161: Hepatitis B | FAS, BAX, BCL2, BRAF, MAPK14, HRAS, CXCL8, KRAS, MYC, NRAS, MAPK1, RAF1, TGFB1, TNF, DDX58 | 4.09E-17 |
| PAXIP1-AS2 | hsa05160: Hepatitis C | FAS, BAX, BRAF, HRAS, KRAS, LDLR, MYC, NRAS, MAPK1, RAF1, TNF, DDX58 | 1.08E-12 |
| PAXIP1-AS2 | hsa04010: MAPK signaling pathway | FAS, BRAF, MAPK14, FGFR3, HRAS, IGF1R, KRAS, MAX, MAP3K11, MYC, NRAS, MAPK1, RAF1, TGFB1, TNF, VEGFA | 1.38E-14 |
| PAXIP1-AS2 | hsa01522: Endocrine resistance | BAX, BCL2, BRAF, CDKN2A, MAPK14, ESR1, ESR2, HRAS, IGF1R, KRAS, NRAS, MAPK1, RAF1 | 6.46E-17 |
| PAXIP1-AS2 | hsa01521: EGFR tyrosine kinase inhibitor resistance | BAX, BCL2, BRAF, FGFR3, HRAS, IGF1R, IL6R, KRAS, NRAS, MAPK1, RAF1, VEGFA | 2.25E-16 |
| PCBP1-AS1 | hsa05219: Bladder cancer | BRAF, CDKN2A, FGFR3, HRAS, CXCL8, KRAS, MYC, NRAS, MAPK1, RAF1, VEGFA | 5.12E-18 |
| PCBP1-AS1 | hsa05205: Proteoglycans in cancer | FAS, BRAF, MAPK14, ESR1, HRAS, IGF1R, KRAS, MYC, NRAS, MAPK1, RAF1, TGFB1, TNF, VEGFA | 4.30E-14 |
| PCBP1-AS1 | hsa05163: Human cytomegalovirus infection | FAS, BAX, CDKN2A, MAPK14, HRAS, IL6R, CXCL8, KRAS, MYC, NRAS, MAPK1, RAF1, TNF, VEGFA | 1.57E-13 |
| PCBP1-AS1 | hsa05161: Hepatitis B | FAS, BAX, BCL2, BRAF, MAPK14, HRAS, CXCL8, KRAS, MYC, NRAS, MAPK1, RAF1, TGFB1, TNF, DDX58 | 4.09E-17 |
| PCBP1-AS1 | hsa05160: Hepatitis C | FAS, BAX, BRAF, HRAS, KRAS, LDLR, MYC, NRAS, MAPK1, RAF1, TNF, DDX58 | 1.08E-12 |
| PCBP1-AS1 | hsa04010: MAPK signaling pathway | FAS, BRAF, MAPK14, FGFR3, HRAS, IGF1R, KRAS, MAX, MAP3K11, MYC, NRAS, MAPK1, RAF1, TGFB1, TNF, VEGFA | 1.38E-14 |
| PCBP1-AS1 | hsa01522: Endocrine resistance | BAX, BCL2, BRAF, CDKN2A, MAPK14, ESR1, ESR2, HRAS, IGF1R, KRAS, NRAS, MAPK1, RAF1 | 6.46E-17 |
| PCBP1-AS1 | hsa01521: EGFR tyrosine kinase inhibitor resistance | BAX, BCL2, BRAF, FGFR3, HRAS, IGF1R, IL6R, KRAS, NRAS, MAPK1, RAF1, VEGFA | 2.25E-16 |
| PRKCQ-AS1 | hsa05163: Human cytomegalovirus infection | FAS, BAX, CDKN2A, MAPK14, HRAS, IL6R, CXCL8, KRAS, MYC, NRAS, MAPK1, RAF1, TNF, VEGFA | 1.57E-13 |
| PRKCQ-AS1 | hsa05161: Hepatitis B | FAS, BAX, BCL2, BRAF, MAPK14, HRAS, CXCL8, KRAS, MYC, NRAS, MAPK1, RAF1, TGFB1, TNF, DDX58 | 4.09E-17 |
| PRKCQ-AS1 | hsa05160: Hepatitis C | FAS, BAX, BRAF, HRAS, KRAS, LDLR, MYC, NRAS, MAPK1, RAF1, TNF, DDX58 | 1.08E-12 |
| PRKCQ-AS1 | hsa01522: Endocrine resistance | BAX, BCL2, BRAF, CDKN2A, MAPK14, ESR1, ESR2, HRAS, IGF1R, KRAS, NRAS, MAPK1, RAF1 | 6.46E-17 |
| PRKCQ-AS1 | hsa01521: EGFR tyrosine kinase inhibitor resistance | BAX, BCL2, BRAF, FGFR3, HRAS, IGF1R, IL6R, KRAS, NRAS, MAPK1, RAF1, VEGFA | 2.25E-16 |
| PSMD5-AS1 | hsa05205: Proteoglycans in cancer | FAS, BRAF, MAPK14, ESR1, HRAS, IGF1R, KRAS, MYC, NRAS, MAPK1, RAF1, TGFB1, TNF, VEGFA | 4.30E-14 |
| PSMD5-AS1 | hsa05163: Human cytomegalovirus infection | FAS, BAX, CDKN2A, MAPK14, HRAS, IL6R, CXCL8, KRAS, MYC, NRAS, MAPK1, RAF1, TNF, VEGFA | 1.57E-13 |
| PSMD5-AS1 | hsa05161: Hepatitis B | FAS, BAX, BCL2, BRAF, MAPK14, HRAS, CXCL8, KRAS, MYC, NRAS, MAPK1, RAF1, TGFB1, TNF, DDX58 | 4.09E-17 |
| PSMD5-AS1 | hsa05160: Hepatitis C | FAS, BAX, BRAF, HRAS, KRAS, LDLR, MYC, NRAS, MAPK1, RAF1, TNF, DDX58 | 1.08E-12 |
| PSMD5-AS1 | hsa04010: MAPK signaling pathway | FAS, BRAF, MAPK14, FGFR3, HRAS, IGF1R, KRAS, MAX, MAP3K11, MYC, NRAS, MAPK1, RAF1, TGFB1, TNF, VEGFA | 1.38E-14 |
| PSMD5-AS1 | hsa01521: EGFR tyrosine kinase inhibitor resistance | BAX, BCL2, BRAF, FGFR3, HRAS, IGF1R, IL6R, KRAS, NRAS, MAPK1, RAF1, VEGFA | 2.25E-16 |
| PTGES2-AS1 | hsa05205: Proteoglycans in cancer | FAS, BRAF, MAPK14, ESR1, HRAS, IGF1R, KRAS, MYC, NRAS, MAPK1, RAF1, TGFB1, TNF, VEGFA | 4.30E-14 |
| PTGES2-AS1 | hsa05163: Human cytomegalovirus infection | FAS, BAX, CDKN2A, MAPK14, HRAS, IL6R, CXCL8, KRAS, MYC, NRAS, MAPK1, RAF1, TNF, VEGFA | 1.57E-13 |
| PTGES2-AS1 | hsa05161: Hepatitis B | FAS, BAX, BCL2, BRAF, MAPK14, HRAS, CXCL8, KRAS, MYC, NRAS, MAPK1, RAF1, TGFB1, TNF, DDX58 | 4.09E-17 |
| PTGES2-AS1 | hsa05160: Hepatitis C | FAS, BAX, BRAF, HRAS, KRAS, LDLR, MYC, NRAS, MAPK1, RAF1, TNF, DDX58 | 1.08E-12 |
| PTGES2-AS1 | hsa04010: MAPK signaling pathway | FAS, BRAF, MAPK14, FGFR3, HRAS, IGF1R, KRAS, MAX, MAP3K11, MYC, NRAS, MAPK1, RAF1, TGFB1, TNF, VEGFA | 1.38E-14 |
| PTGES2-AS1 | hsa01521: EGFR tyrosine kinase inhibitor resistance | BAX, BCL2, BRAF, FGFR3, HRAS, IGF1R, IL6R, KRAS, NRAS, MAPK1, RAF1, VEGFA | 2.25E-16 |
| PVT1 | hsa05219: Bladder cancer | BRAF, CDKN2A, FGFR3, HRAS, CXCL8, KRAS, MYC, NRAS, MAPK1, RAF1, VEGFA | 5.12E-18 |
| PVT1 | hsa05205: Proteoglycans in cancer | FAS, BRAF, MAPK14, ESR1, HRAS, IGF1R, KRAS, MYC, NRAS, MAPK1, RAF1, TGFB1, TNF, VEGFA | 4.30E-14 |
| PVT1 | hsa05161: Hepatitis B | FAS, BAX, BCL2, BRAF, MAPK14, HRAS, CXCL8, KRAS, MYC, NRAS, MAPK1, RAF1, TGFB1, TNF, DDX58 | 4.09E-17 |
| PVT1 | hsa05160: Hepatitis C | FAS, BAX, BRAF, HRAS, KRAS, LDLR, MYC, NRAS, MAPK1, RAF1, TNF, DDX58 | 1.08E-12 |
| PVT1 | hsa04010: MAPK signaling pathway | FAS, BRAF, MAPK14, FGFR3, HRAS, IGF1R, KRAS, MAX, MAP3K11, MYC, NRAS, MAPK1, RAF1, TGFB1, TNF, VEGFA | 1.38E-14 |
| PVT1 | hsa01522: Endocrine resistance | BAX, BCL2, BRAF, CDKN2A, MAPK14, ESR1, ESR2, HRAS, IGF1R, KRAS, NRAS, MAPK1, RAF1 | 6.46E-17 |
| PVT1 | hsa01521: EGFR tyrosine kinase inhibitor resistance | BAX, BCL2, BRAF, FGFR3, HRAS, IGF1R, IL6R, KRAS, NRAS, MAPK1, RAF1, VEGFA | 2.25E-16 |
| RBM12B-AS1 | hsa05205: Proteoglycans in cancer | FAS, BRAF, MAPK14, ESR1, HRAS, IGF1R, KRAS, MYC, NRAS, MAPK1, RAF1, TGFB1, TNF, VEGFA | 4.30E-14 |
| RBM12B-AS1 | hsa01522: Endocrine resistance | BAX, BCL2, BRAF, CDKN2A, MAPK14, ESR1, ESR2, HRAS, IGF1R, KRAS, NRAS, MAPK1, RAF1 | 6.46E-17 |
| RUSC1-AS1 | hsa05219: Bladder cancer | BRAF, CDKN2A, FGFR3, HRAS, CXCL8, KRAS, MYC, NRAS, MAPK1, RAF1, VEGFA | 5.12E-18 |
| RUSC1-AS1 | hsa05205: Proteoglycans in cancer | FAS, BRAF, MAPK14, ESR1, HRAS, IGF1R, KRAS, MYC, NRAS, MAPK1, RAF1, TGFB1, TNF, VEGFA | 4.30E-14 |
| RUSC1-AS1 | hsa05163: Human cytomegalovirus infection | FAS, BAX, CDKN2A, MAPK14, HRAS, IL6R, CXCL8, KRAS, MYC, NRAS, MAPK1, RAF1, TNF, VEGFA | 1.57E-13 |
| RUSC1-AS1 | hsa05161: Hepatitis B | FAS, BAX, BCL2, BRAF, MAPK14, HRAS, CXCL8, KRAS, MYC, NRAS, MAPK1, RAF1, TGFB1, TNF, DDX58 | 4.09E-17 |
| RUSC1-AS1 | hsa05160: Hepatitis C | FAS, BAX, BRAF, HRAS, KRAS, LDLR, MYC, NRAS, MAPK1, RAF1, TNF, DDX58 | 1.08E-12 |
| RUSC1-AS1 | hsa04010: MAPK signaling pathway | FAS, BRAF, MAPK14, FGFR3, HRAS, IGF1R, KRAS, MAX, MAP3K11, MYC, NRAS, MAPK1, RAF1, TGFB1, TNF, VEGFA | 1.38E-14 |
| RUSC1-AS1 | hsa01522: Endocrine resistance | BAX, BCL2, BRAF, CDKN2A, MAPK14, ESR1, ESR2, HRAS, IGF1R, KRAS, NRAS, MAPK1, RAF1 | 6.46E-17 |
| RUSC1-AS1 | hsa01521: EGFR tyrosine kinase inhibitor resistance | BAX, BCL2, BRAF, FGFR3, HRAS, IGF1R, IL6R, KRAS, NRAS, MAPK1, RAF1, VEGFA | 2.25E-16 |
| SNAI3-AS1 | hsa05219: Bladder cancer | BRAF, CDKN2A, FGFR3, HRAS, CXCL8, KRAS, MYC, NRAS, MAPK1, RAF1, VEGFA | 5.12E-18 |
| SNAI3-AS1 | hsa05205: Proteoglycans in cancer | FAS, BRAF, MAPK14, ESR1, HRAS, IGF1R, KRAS, MYC, NRAS, MAPK1, RAF1, TGFB1, TNF, VEGFA | 4.30E-14 |
| SNAI3-AS1 | hsa05163: Human cytomegalovirus infection | FAS, BAX, CDKN2A, MAPK14, HRAS, IL6R, CXCL8, KRAS, MYC, NRAS, MAPK1, RAF1, TNF, VEGFA | 1.57E-13 |
| SNAI3-AS1 | hsa05161: Hepatitis B | FAS, BAX, BCL2, BRAF, MAPK14, HRAS, CXCL8, KRAS, MYC, NRAS, MAPK1, RAF1, TGFB1, TNF, DDX58 | 4.09E-17 |
| SNAI3-AS1 | hsa05160: Hepatitis C | FAS, BAX, BRAF, HRAS, KRAS, LDLR, MYC, NRAS, MAPK1, RAF1, TNF, DDX58 | 1.08E-12 |
| SNAI3-AS1 | hsa04010: MAPK signaling pathway | FAS, BRAF, MAPK14, FGFR3, HRAS, IGF1R, KRAS, MAX, MAP3K11, MYC, NRAS, MAPK1, RAF1, TGFB1, TNF, VEGFA | 1.38E-14 |
| SNAI3-AS1 | hsa01522: Endocrine resistance | BAX, BCL2, BRAF, CDKN2A, MAPK14, ESR1, ESR2, HRAS, IGF1R, KRAS, NRAS, MAPK1, RAF1 | 6.46E-17 |
| SNAI3-AS1 | hsa01521: EGFR tyrosine kinase inhibitor resistance | BAX, BCL2, BRAF, FGFR3, HRAS, IGF1R, IL6R, KRAS, NRAS, MAPK1, RAF1, VEGFA | 2.25E-16 |
| SND1-IT1 | hsa05219: Bladder cancer | BRAF, CDKN2A, FGFR3, HRAS, CXCL8, KRAS, MYC, NRAS, MAPK1, RAF1, VEGFA | 5.12E-18 |
| SND1-IT1 | hsa05205: Proteoglycans in cancer | FAS, BRAF, MAPK14, ESR1, HRAS, IGF1R, KRAS, MYC, NRAS, MAPK1, RAF1, TGFB1, TNF, VEGFA | 4.30E-14 |
| SND1-IT1 | hsa05163: Human cytomegalovirus infection | FAS, BAX, CDKN2A, MAPK14, HRAS, IL6R, CXCL8, KRAS, MYC, NRAS, MAPK1, RAF1, TNF, VEGFA | 1.57E-13 |
| SND1-IT1 | hsa05161: Hepatitis B | FAS, BAX, BCL2, BRAF, MAPK14, HRAS, CXCL8, KRAS, MYC, NRAS, MAPK1, RAF1, TGFB1, TNF, DDX58 | 4.09E-17 |
| SND1-IT1 | hsa05160: Hepatitis C | FAS, BAX, BRAF, HRAS, KRAS, LDLR, MYC, NRAS, MAPK1, RAF1, TNF, DDX58 | 1.08E-12 |
| SND1-IT1 | hsa04010: MAPK signaling pathway | FAS, BRAF, MAPK14, FGFR3, HRAS, IGF1R, KRAS, MAX, MAP3K11, MYC, NRAS, MAPK1, RAF1, TGFB1, TNF, VEGFA | 1.38E-14 |
| SND1-IT1 | hsa01522: Endocrine resistance | BAX, BCL2, BRAF, CDKN2A, MAPK14, ESR1, ESR2, HRAS, IGF1R, KRAS, NRAS, MAPK1, RAF1 | 6.46E-17 |
| SND1-IT1 | hsa01521: EGFR tyrosine kinase inhibitor resistance | BAX, BCL2, BRAF, FGFR3, HRAS, IGF1R, IL6R, KRAS, NRAS, MAPK1, RAF1, VEGFA | 2.25E-16 |
| SNHG1 | hsa01522: Endocrine resistance | BAX, BCL2, BRAF, CDKN2A, MAPK14, ESR1, ESR2, HRAS, IGF1R, KRAS, NRAS, MAPK1, RAF1 | 6.46E-17 |
| SNHG11 | hsa05163: Human cytomegalovirus infection | FAS, BAX, CDKN2A, MAPK14, HRAS, IL6R, CXCL8, KRAS, MYC, NRAS, MAPK1, RAF1, TNF, VEGFA | 1.57E-13 |
| SNHG11 | hsa05161: Hepatitis B | FAS, BAX, BCL2, BRAF, MAPK14, HRAS, CXCL8, KRAS, MYC, NRAS, MAPK1, RAF1, TGFB1, TNF, DDX58 | 4.09E-17 |
| SNHG11 | hsa05160: Hepatitis C | FAS, BAX, BRAF, HRAS, KRAS, LDLR, MYC, NRAS, MAPK1, RAF1, TNF, DDX58 | 1.08E-12 |
| SNHG11 | hsa01522: Endocrine resistance | BAX, BCL2, BRAF, CDKN2A, MAPK14, ESR1, ESR2, HRAS, IGF1R, KRAS, NRAS, MAPK1, RAF1 | 6.46E-17 |
| SNHG11 | hsa01521: EGFR tyrosine kinase inhibitor resistance | BAX, BCL2, BRAF, FGFR3, HRAS, IGF1R, IL6R, KRAS, NRAS, MAPK1, RAF1, VEGFA | 2.25E-16 |
| SNHG20 | hsa05161: Hepatitis B | FAS, BAX, BCL2, BRAF, MAPK14, HRAS, CXCL8, KRAS, MYC, NRAS, MAPK1, RAF1, TGFB1, TNF, DDX58 | 4.09E-17 |
| SNHG20 | hsa05160: Hepatitis C | FAS, BAX, BRAF, HRAS, KRAS, LDLR, MYC, NRAS, MAPK1, RAF1, TNF, DDX58 | 1.08E-12 |
| SNHG4 | hsa05161: Hepatitis B | FAS, BAX, BCL2, BRAF, MAPK14, HRAS, CXCL8, KRAS, MYC, NRAS, MAPK1, RAF1, TGFB1, TNF, DDX58 | 4.09E-17 |
| SNHG4 | hsa01522: Endocrine resistance | BAX, BCL2, BRAF, CDKN2A, MAPK14, ESR1, ESR2, HRAS, IGF1R, KRAS, NRAS, MAPK1, RAF1 | 6.46E-17 |
| SNHG4 | hsa01521: EGFR tyrosine kinase inhibitor resistance | BAX, BCL2, BRAF, FGFR3, HRAS, IGF1R, IL6R, KRAS, NRAS, MAPK1, RAF1, VEGFA | 2.25E-16 |
| SNX29P2 | hsa01522: Endocrine resistance | BAX, BCL2, BRAF, CDKN2A, MAPK14, ESR1, ESR2, HRAS, IGF1R, KRAS, NRAS, MAPK1, RAF1 | 6.46E-17 |
| SRP14-AS1 | hsa05219: Bladder cancer | BRAF, CDKN2A, FGFR3, HRAS, CXCL8, KRAS, MYC, NRAS, MAPK1, RAF1, VEGFA | 5.12E-18 |
| SRP14-AS1 | hsa05205: Proteoglycans in cancer | FAS, BRAF, MAPK14, ESR1, HRAS, IGF1R, KRAS, MYC, NRAS, MAPK1, RAF1, TGFB1, TNF, VEGFA | 4.30E-14 |
| SRP14-AS1 | hsa05163: Human cytomegalovirus infection | FAS, BAX, CDKN2A, MAPK14, HRAS, IL6R, CXCL8, KRAS, MYC, NRAS, MAPK1, RAF1, TNF, VEGFA | 1.57E-13 |
| SRP14-AS1 | hsa05161: Hepatitis B | FAS, BAX, BCL2, BRAF, MAPK14, HRAS, CXCL8, KRAS, MYC, NRAS, MAPK1, RAF1, TGFB1, TNF, DDX58 | 4.09E-17 |
| SRP14-AS1 | hsa05160: Hepatitis C | FAS, BAX, BRAF, HRAS, KRAS, LDLR, MYC, NRAS, MAPK1, RAF1, TNF, DDX58 | 1.08E-12 |
| SRP14-AS1 | hsa04010: MAPK signaling pathway | FAS, BRAF, MAPK14, FGFR3, HRAS, IGF1R, KRAS, MAX, MAP3K11, MYC, NRAS, MAPK1, RAF1, TGFB1, TNF, VEGFA | 1.38E-14 |
| SRP14-AS1 | hsa01522: Endocrine resistance | BAX, BCL2, BRAF, CDKN2A, MAPK14, ESR1, ESR2, HRAS, IGF1R, KRAS, NRAS, MAPK1, RAF1 | 6.46E-17 |
| SRP14-AS1 | hsa01521: EGFR tyrosine kinase inhibitor resistance | BAX, BCL2, BRAF, FGFR3, HRAS, IGF1R, IL6R, KRAS, NRAS, MAPK1, RAF1, VEGFA | 2.25E-16 |
| STARD7-AS1 | hsa01522: Endocrine resistance | BAX, BCL2, BRAF, CDKN2A, MAPK14, ESR1, ESR2, HRAS, IGF1R, KRAS, NRAS, MAPK1, RAF1 | 6.46E-17 |
| TMEM191A | hsa05163: Human cytomegalovirus infection | FAS, BAX, CDKN2A, MAPK14, HRAS, IL6R, CXCL8, KRAS, MYC, NRAS, MAPK1, RAF1, TNF, VEGFA | 1.57E-13 |
| TMEM191A | hsa05161: Hepatitis B | FAS, BAX, BCL2, BRAF, MAPK14, HRAS, CXCL8, KRAS, MYC, NRAS, MAPK1, RAF1, TGFB1, TNF, DDX58 | 4.09E-17 |
| TMEM191A | hsa05160: Hepatitis C | FAS, BAX, BRAF, HRAS, KRAS, LDLR, MYC, NRAS, MAPK1, RAF1, TNF, DDX58 | 1.08E-12 |
| TMEM191A | hsa01522: Endocrine resistance | BAX, BCL2, BRAF, CDKN2A, MAPK14, ESR1, ESR2, HRAS, IGF1R, KRAS, NRAS, MAPK1, RAF1 | 6.46E-17 |
| TMEM191A | hsa01521: EGFR tyrosine kinase inhibitor resistance | BAX, BCL2, BRAF, FGFR3, HRAS, IGF1R, IL6R, KRAS, NRAS, MAPK1, RAF1, VEGFA | 2.25E-16 |
| TNRC6C-AS1 | hsa05205: Proteoglycans in cancer | FAS, BRAF, MAPK14, ESR1, HRAS, IGF1R, KRAS, MYC, NRAS, MAPK1, RAF1, TGFB1, TNF, VEGFA | 4.30E-14 |
| TNRC6C-AS1 | hsa01522: Endocrine resistance | BAX, BCL2, BRAF, CDKN2A, MAPK14, ESR1, ESR2, HRAS, IGF1R, KRAS, NRAS, MAPK1, RAF1 | 6.46E-17 |
| TOB1-AS1 | hsa05205: Proteoglycans in cancer | FAS, BRAF, MAPK14, ESR1, HRAS, IGF1R, KRAS, MYC, NRAS, MAPK1, RAF1, TGFB1, TNF, VEGFA | 4.30E-14 |
| TOB1-AS1 | hsa05163: Human cytomegalovirus infection | FAS, BAX, CDKN2A, MAPK14, HRAS, IL6R, CXCL8, KRAS, MYC, NRAS, MAPK1, RAF1, TNF, VEGFA | 1.57E-13 |
| TOB1-AS1 | hsa05161: Hepatitis B | FAS, BAX, BCL2, BRAF, MAPK14, HRAS, CXCL8, KRAS, MYC, NRAS, MAPK1, RAF1, TGFB1, TNF, DDX58 | 4.09E-17 |
| TOB1-AS1 | hsa05160: Hepatitis C | FAS, BAX, BRAF, HRAS, KRAS, LDLR, MYC, NRAS, MAPK1, RAF1, TNF, DDX58 | 1.08E-12 |
| TOB1-AS1 | hsa04010: MAPK signaling pathway | FAS, BRAF, MAPK14, FGFR3, HRAS, IGF1R, KRAS, MAX, MAP3K11, MYC, NRAS, MAPK1, RAF1, TGFB1, TNF, VEGFA | 1.38E-14 |
| TOB1-AS1 | hsa01521: EGFR tyrosine kinase inhibitor resistance | BAX, BCL2, BRAF, FGFR3, HRAS, IGF1R, IL6R, KRAS, NRAS, MAPK1, RAF1, VEGFA | 2.25E-16 |
| TOPORS-AS1 | hsa05219: Bladder cancer | BRAF, CDKN2A, FGFR3, HRAS, CXCL8, KRAS, MYC, NRAS, MAPK1, RAF1, VEGFA | 5.12E-18 |
| TOPORS-AS1 | hsa05205: Proteoglycans in cancer | FAS, BRAF, MAPK14, ESR1, HRAS, IGF1R, KRAS, MYC, NRAS, MAPK1, RAF1, TGFB1, TNF, VEGFA | 4.30E-14 |
| TOPORS-AS1 | hsa05163: Human cytomegalovirus infection | FAS, BAX, CDKN2A, MAPK14, HRAS, IL6R, CXCL8, KRAS, MYC, NRAS, MAPK1, RAF1, TNF, VEGFA | 1.57E-13 |
| TOPORS-AS1 | hsa05161: Hepatitis B | FAS, BAX, BCL2, BRAF, MAPK14, HRAS, CXCL8, KRAS, MYC, NRAS, MAPK1, RAF1, TGFB1, TNF, DDX58 | 4.09E-17 |
| TOPORS-AS1 | hsa05160: Hepatitis C | FAS, BAX, BRAF, HRAS, KRAS, LDLR, MYC, NRAS, MAPK1, RAF1, TNF, DDX58 | 1.08E-12 |
| TOPORS-AS1 | hsa04010: MAPK signaling pathway | FAS, BRAF, MAPK14, FGFR3, HRAS, IGF1R, KRAS, MAX, MAP3K11, MYC, NRAS, MAPK1, RAF1, TGFB1, TNF, VEGFA | 1.38E-14 |
| TOPORS-AS1 | hsa01522: Endocrine resistance | BAX, BCL2, BRAF, CDKN2A, MAPK14, ESR1, ESR2, HRAS, IGF1R, KRAS, NRAS, MAPK1, RAF1 | 6.46E-17 |
| TOPORS-AS1 | hsa01521: EGFR tyrosine kinase inhibitor resistance | BAX, BCL2, BRAF, FGFR3, HRAS, IGF1R, IL6R, KRAS, NRAS, MAPK1, RAF1, VEGFA | 2.25E-16 |
| TP73-AS1 | hsa01522: Endocrine resistance | BAX, BCL2, BRAF, CDKN2A, MAPK14, ESR1, ESR2, HRAS, IGF1R, KRAS, NRAS, MAPK1, RAF1 | 6.46E-17 |
| TRAF3IP2-AS1 | hsa05219: Bladder cancer | BRAF, CDKN2A, FGFR3, HRAS, CXCL8, KRAS, MYC, NRAS, MAPK1, RAF1, VEGFA | 5.12E-18 |
| TRAF3IP2-AS1 | hsa05205: Proteoglycans in cancer | FAS, BRAF, MAPK14, ESR1, HRAS, IGF1R, KRAS, MYC, NRAS, MAPK1, RAF1, TGFB1, TNF, VEGFA | 4.30E-14 |
| TRAF3IP2-AS1 | hsa05163: Human cytomegalovirus infection | FAS, BAX, CDKN2A, MAPK14, HRAS, IL6R, CXCL8, KRAS, MYC, NRAS, MAPK1, RAF1, TNF, VEGFA | 1.57E-13 |
| TRAF3IP2-AS1 | hsa05161: Hepatitis B | FAS, BAX, BCL2, BRAF, MAPK14, HRAS, CXCL8, KRAS, MYC, NRAS, MAPK1, RAF1, TGFB1, TNF, DDX58 | 4.09E-17 |
| TRAF3IP2-AS1 | hsa05160: Hepatitis C | FAS, BAX, BRAF, HRAS, KRAS, LDLR, MYC, NRAS, MAPK1, RAF1, TNF, DDX58 | 1.08E-12 |
| TRAF3IP2-AS1 | hsa04010: MAPK signaling pathway | FAS, BRAF, MAPK14, FGFR3, HRAS, IGF1R, KRAS, MAX, MAP3K11, MYC, NRAS, MAPK1, RAF1, TGFB1, TNF, VEGFA | 1.38E-14 |
| TRAF3IP2-AS1 | hsa01522: Endocrine resistance | BAX, BCL2, BRAF, CDKN2A, MAPK14, ESR1, ESR2, HRAS, IGF1R, KRAS, NRAS, MAPK1, RAF1 | 6.46E-17 |
| TRAF3IP2-AS1 | hsa01521: EGFR tyrosine kinase inhibitor resistance | BAX, BCL2, BRAF, FGFR3, HRAS, IGF1R, IL6R, KRAS, NRAS, MAPK1, RAF1, VEGFA | 2.25E-16 |
| TRAM2-AS1 | hsa05219: Bladder cancer | BRAF, CDKN2A, FGFR3, HRAS, CXCL8, KRAS, MYC, NRAS, MAPK1, RAF1, VEGFA | 5.12E-18 |
| TRAM2-AS1 | hsa05205: Proteoglycans in cancer | FAS, BRAF, MAPK14, ESR1, HRAS, IGF1R, KRAS, MYC, NRAS, MAPK1, RAF1, TGFB1, TNF, VEGFA | 4.30E-14 |
| TRAM2-AS1 | hsa05163: Human cytomegalovirus infection | FAS, BAX, CDKN2A, MAPK14, HRAS, IL6R, CXCL8, KRAS, MYC, NRAS, MAPK1, RAF1, TNF, VEGFA | 1.57E-13 |
| TRAM2-AS1 | hsa05161: Hepatitis B | FAS, BAX, BCL2, BRAF, MAPK14, HRAS, CXCL8, KRAS, MYC, NRAS, MAPK1, RAF1, TGFB1, TNF, DDX58 | 4.09E-17 |
| TRAM2-AS1 | hsa05160: Hepatitis C | FAS, BAX, BRAF, HRAS, KRAS, LDLR, MYC, NRAS, MAPK1, RAF1, TNF, DDX58 | 1.08E-12 |
| TRAM2-AS1 | hsa04010: MAPK signaling pathway | FAS, BRAF, MAPK14, FGFR3, HRAS, IGF1R, KRAS, MAX, MAP3K11, MYC, NRAS, MAPK1, RAF1, TGFB1, TNF, VEGFA | 1.38E-14 |
| TRAM2-AS1 | hsa01522: Endocrine resistance | BAX, BCL2, BRAF, CDKN2A, MAPK14, ESR1, ESR2, HRAS, IGF1R, KRAS, NRAS, MAPK1, RAF1 | 6.46E-17 |
| TRAM2-AS1 | hsa01521: EGFR tyrosine kinase inhibitor resistance | BAX, BCL2, BRAF, FGFR3, HRAS, IGF1R, IL6R, KRAS, NRAS, MAPK1, RAF1, VEGFA | 2.25E-16 |
| TTTY15 | hsa05161: Hepatitis B | FAS, BAX, BCL2, BRAF, MAPK14, HRAS, CXCL8, KRAS, MYC, NRAS, MAPK1, RAF1, TGFB1, TNF, DDX58 | 4.09E-17 |
| TTTY15 | hsa01522: Endocrine resistance | BAX, BCL2, BRAF, CDKN2A, MAPK14, ESR1, ESR2, HRAS, IGF1R, KRAS, NRAS, MAPK1, RAF1 | 6.46E-17 |
| TTTY15 | hsa01521: EGFR tyrosine kinase inhibitor resistance | BAX, BCL2, BRAF, FGFR3, HRAS, IGF1R, IL6R, KRAS, NRAS, MAPK1, RAF1, VEGFA | 2.25E-16 |
| TUG1 | hsa05219: Bladder cancer | BRAF, CDKN2A, FGFR3, HRAS, CXCL8, KRAS, MYC, NRAS, MAPK1, RAF1, VEGFA | 5.12E-18 |
| TUG1 | hsa05205: Proteoglycans in cancer | FAS, BRAF, MAPK14, ESR1, HRAS, IGF1R, KRAS, MYC, NRAS, MAPK1, RAF1, TGFB1, TNF, VEGFA | 4.30E-14 |
| TUG1 | hsa05163: Human cytomegalovirus infection | FAS, BAX, CDKN2A, MAPK14, HRAS, IL6R, CXCL8, KRAS, MYC, NRAS, MAPK1, RAF1, TNF, VEGFA | 1.57E-13 |
| TUG1 | hsa05161: Hepatitis B | FAS, BAX, BCL2, BRAF, MAPK14, HRAS, CXCL8, KRAS, MYC, NRAS, MAPK1, RAF1, TGFB1, TNF, DDX58 | 4.09E-17 |
| TUG1 | hsa05160: Hepatitis C | FAS, BAX, BRAF, HRAS, KRAS, LDLR, MYC, NRAS, MAPK1, RAF1, TNF, DDX58 | 1.08E-12 |
| TUG1 | hsa04010: MAPK signaling pathway | FAS, BRAF, MAPK14, FGFR3, HRAS, IGF1R, KRAS, MAX, MAP3K11, MYC, NRAS, MAPK1, RAF1, TGFB1, TNF, VEGFA | 1.38E-14 |
| TUG1 | hsa01522: Endocrine resistance | BAX, BCL2, BRAF, CDKN2A, MAPK14, ESR1, ESR2, HRAS, IGF1R, KRAS, NRAS, MAPK1, RAF1 | 6.46E-17 |
| TUG1 | hsa01521: EGFR tyrosine kinase inhibitor resistance | BAX, BCL2, BRAF, FGFR3, HRAS, IGF1R, IL6R, KRAS, NRAS, MAPK1, RAF1, VEGFA | 2.25E-16 |
| UBA6-AS1 | hsa05163: Human cytomegalovirus infection | FAS, BAX, CDKN2A, MAPK14, HRAS, IL6R, CXCL8, KRAS, MYC, NRAS, MAPK1, RAF1, TNF, VEGFA | 1.57E-13 |
| UBA6-AS1 | hsa05161: Hepatitis B | FAS, BAX, BCL2, BRAF, MAPK14, HRAS, CXCL8, KRAS, MYC, NRAS, MAPK1, RAF1, TGFB1, TNF, DDX58 | 4.09E-17 |
| UBA6-AS1 | hsa05160: Hepatitis C | FAS, BAX, BRAF, HRAS, KRAS, LDLR, MYC, NRAS, MAPK1, RAF1, TNF, DDX58 | 1.08E-12 |
| UBA6-AS1 | hsa01522: Endocrine resistance | BAX, BCL2, BRAF, CDKN2A, MAPK14, ESR1, ESR2, HRAS, IGF1R, KRAS, NRAS, MAPK1, RAF1 | 6.46E-17 |
| UBA6-AS1 | hsa01521: EGFR tyrosine kinase inhibitor resistance | BAX, BCL2, BRAF, FGFR3, HRAS, IGF1R, IL6R, KRAS, NRAS, MAPK1, RAF1, VEGFA | 2.25E-16 |
| UBL7-AS1 | hsa05205: Proteoglycans in cancer | FAS, BRAF, MAPK14, ESR1, HRAS, IGF1R, KRAS, MYC, NRAS, MAPK1, RAF1, TGFB1, TNF, VEGFA | 4.30E-14 |
| UBL7-AS1 | hsa01522: Endocrine resistance | BAX, BCL2, BRAF, CDKN2A, MAPK14, ESR1, ESR2, HRAS, IGF1R, KRAS, NRAS, MAPK1, RAF1 | 6.46E-17 |
| UBOX5-AS1 | hsa01522: Endocrine resistance | BAX, BCL2, BRAF, CDKN2A, MAPK14, ESR1, ESR2, HRAS, IGF1R, KRAS, NRAS, MAPK1, RAF1 | 6.46E-17 |
| URB1-AS1 | hsa05219: Bladder cancer | BRAF, CDKN2A, FGFR3, HRAS, CXCL8, KRAS, MYC, NRAS, MAPK1, RAF1, VEGFA | 5.12E-18 |
| URB1-AS1 | hsa05205: Proteoglycans in cancer | FAS, BRAF, MAPK14, ESR1, HRAS, IGF1R, KRAS, MYC, NRAS, MAPK1, RAF1, TGFB1, TNF, VEGFA | 4.30E-14 |
| URB1-AS1 | hsa05163: Human cytomegalovirus infection | FAS, BAX, CDKN2A, MAPK14, HRAS, IL6R, CXCL8, KRAS, MYC, NRAS, MAPK1, RAF1, TNF, VEGFA | 1.57E-13 |
| URB1-AS1 | hsa05161: Hepatitis B | FAS, BAX, BCL2, BRAF, MAPK14, HRAS, CXCL8, KRAS, MYC, NRAS, MAPK1, RAF1, TGFB1, TNF, DDX58 | 4.09E-17 |
| URB1-AS1 | hsa05160: Hepatitis C | FAS, BAX, BRAF, HRAS, KRAS, LDLR, MYC, NRAS, MAPK1, RAF1, TNF, DDX58 | 1.08E-12 |
| URB1-AS1 | hsa04010: MAPK signaling pathway | FAS, BRAF, MAPK14, FGFR3, HRAS, IGF1R, KRAS, MAX, MAP3K11, MYC, NRAS, MAPK1, RAF1, TGFB1, TNF, VEGFA | 1.38E-14 |
| URB1-AS1 | hsa01522: Endocrine resistance | BAX, BCL2, BRAF, CDKN2A, MAPK14, ESR1, ESR2, HRAS, IGF1R, KRAS, NRAS, MAPK1, RAF1 | 6.46E-17 |
| URB1-AS1 | hsa01521: EGFR tyrosine kinase inhibitor resistance | BAX, BCL2, BRAF, FGFR3, HRAS, IGF1R, IL6R, KRAS, NRAS, MAPK1, RAF1, VEGFA | 2.25E-16 |
| USP27X-AS1 | hsa05205: Proteoglycans in cancer | FAS, BRAF, MAPK14, ESR1, HRAS, IGF1R, KRAS, MYC, NRAS, MAPK1, RAF1, TGFB1, TNF, VEGFA | 4.30E-14 |
| USP27X-AS1 | hsa05163: Human cytomegalovirus infection | FAS, BAX, CDKN2A, MAPK14, HRAS, IL6R, CXCL8, KRAS, MYC, NRAS, MAPK1, RAF1, TNF, VEGFA | 1.57E-13 |
| USP27X-AS1 | hsa05161: Hepatitis B | FAS, BAX, BCL2, BRAF, MAPK14, HRAS, CXCL8, KRAS, MYC, NRAS, MAPK1, RAF1, TGFB1, TNF, DDX58 | 4.09E-17 |
| USP27X-AS1 | hsa05160: Hepatitis C | FAS, BAX, BRAF, HRAS, KRAS, LDLR, MYC, NRAS, MAPK1, RAF1, TNF, DDX58 | 1.08E-12 |
| USP27X-AS1 | hsa04010: MAPK signaling pathway | FAS, BRAF, MAPK14, FGFR3, HRAS, IGF1R, KRAS, MAX, MAP3K11, MYC, NRAS, MAPK1, RAF1, TGFB1, TNF, VEGFA | 1.38E-14 |
| VAC14-AS1 | hsa05219: Bladder cancer | BRAF, CDKN2A, FGFR3, HRAS, CXCL8, KRAS, MYC, NRAS, MAPK1, RAF1, VEGFA | 5.12E-18 |
| VAC14-AS1 | hsa05205: Proteoglycans in cancer | FAS, BRAF, MAPK14, ESR1, HRAS, IGF1R, KRAS, MYC, NRAS, MAPK1, RAF1, TGFB1, TNF, VEGFA | 4.30E-14 |
| VAC14-AS1 | hsa05163: Human cytomegalovirus infection | FAS, BAX, CDKN2A, MAPK14, HRAS, IL6R, CXCL8, KRAS, MYC, NRAS, MAPK1, RAF1, TNF, VEGFA | 1.57E-13 |
| VAC14-AS1 | hsa05161: Hepatitis B | FAS, BAX, BCL2, BRAF, MAPK14, HRAS, CXCL8, KRAS, MYC, NRAS, MAPK1, RAF1, TGFB1, TNF, DDX58 | 4.09E-17 |
| VAC14-AS1 | hsa05160: Hepatitis C | FAS, BAX, BRAF, HRAS, KRAS, LDLR, MYC, NRAS, MAPK1, RAF1, TNF, DDX58 | 1.08E-12 |
| VAC14-AS1 | hsa04010: MAPK signaling pathway | FAS, BRAF, MAPK14, FGFR3, HRAS, IGF1R, KRAS, MAX, MAP3K11, MYC, NRAS, MAPK1, RAF1, TGFB1, TNF, VEGFA | 1.38E-14 |
| VAC14-AS1 | hsa01522: Endocrine resistance | BAX, BCL2, BRAF, CDKN2A, MAPK14, ESR1, ESR2, HRAS, IGF1R, KRAS, NRAS, MAPK1, RAF1 | 6.46E-17 |
| VAC14-AS1 | hsa01521: EGFR tyrosine kinase inhibitor resistance | BAX, BCL2, BRAF, FGFR3, HRAS, IGF1R, IL6R, KRAS, NRAS, MAPK1, RAF1, VEGFA | 2.25E-16 |
| ZBED5-AS1 | hsa05219: Bladder cancer | BRAF, CDKN2A, FGFR3, HRAS, CXCL8, KRAS, MYC, NRAS, MAPK1, RAF1, VEGFA | 5.12E-18 |
| ZBED5-AS1 | hsa05205: Proteoglycans in cancer | FAS, BRAF, MAPK14, ESR1, HRAS, IGF1R, KRAS, MYC, NRAS, MAPK1, RAF1, TGFB1, TNF, VEGFA | 4.30E-14 |
| ZBED5-AS1 | hsa05163: Human cytomegalovirus infection | FAS, BAX, CDKN2A, MAPK14, HRAS, IL6R, CXCL8, KRAS, MYC, NRAS, MAPK1, RAF1, TNF, VEGFA | 1.57E-13 |
| ZBED5-AS1 | hsa05161: Hepatitis B | FAS, BAX, BCL2, BRAF, MAPK14, HRAS, CXCL8, KRAS, MYC, NRAS, MAPK1, RAF1, TGFB1, TNF, DDX58 | 4.09E-17 |
| ZBED5-AS1 | hsa05160: Hepatitis C | FAS, BAX, BRAF, HRAS, KRAS, LDLR, MYC, NRAS, MAPK1, RAF1, TNF, DDX58 | 1.08E-12 |
| ZBED5-AS1 | hsa04010: MAPK signaling pathway | FAS, BRAF, MAPK14, FGFR3, HRAS, IGF1R, KRAS, MAX, MAP3K11, MYC, NRAS, MAPK1, RAF1, TGFB1, TNF, VEGFA | 1.38E-14 |
| ZBED5-AS1 | hsa01522: Endocrine resistance | BAX, BCL2, BRAF, CDKN2A, MAPK14, ESR1, ESR2, HRAS, IGF1R, KRAS, NRAS, MAPK1, RAF1 | 6.46E-17 |
| ZBED5-AS1 | hsa01521: EGFR tyrosine kinase inhibitor resistance | BAX, BCL2, BRAF, FGFR3, HRAS, IGF1R, IL6R, KRAS, NRAS, MAPK1, RAF1, VEGFA | 2.25E-16 |
| ZBTB11-AS1 | hsa05163: Human cytomegalovirus infection | FAS, BAX, CDKN2A, MAPK14, HRAS, IL6R, CXCL8, KRAS, MYC, NRAS, MAPK1, RAF1, TNF, VEGFA | 1.57E-13 |
| ZBTB11-AS1 | hsa05161: Hepatitis B | FAS, BAX, BCL2, BRAF, MAPK14, HRAS, CXCL8, KRAS, MYC, NRAS, MAPK1, RAF1, TGFB1, TNF, DDX58 | 4.09E-17 |
| ZBTB11-AS1 | hsa05160: Hepatitis C | FAS, BAX, BRAF, HRAS, KRAS, LDLR, MYC, NRAS, MAPK1, RAF1, TNF, DDX58 | 1.08E-12 |
| ZBTB11-AS1 | hsa01522: Endocrine resistance | BAX, BCL2, BRAF, CDKN2A, MAPK14, ESR1, ESR2, HRAS, IGF1R, KRAS, NRAS, MAPK1, RAF1 | 6.46E-17 |
| ZBTB11-AS1 | hsa01521: EGFR tyrosine kinase inhibitor resistance | BAX, BCL2, BRAF, FGFR3, HRAS, IGF1R, IL6R, KRAS, NRAS, MAPK1, RAF1, VEGFA | 2.25E-16 |
| ZFAS1 | hsa05219: Bladder cancer | BRAF, CDKN2A, FGFR3, HRAS, CXCL8, KRAS, MYC, NRAS, MAPK1, RAF1, VEGFA | 5.12E-18 |
| ZFAS1 | hsa05205: Proteoglycans in cancer | FAS, BRAF, MAPK14, ESR1, HRAS, IGF1R, KRAS, MYC, NRAS, MAPK1, RAF1, TGFB1, TNF, VEGFA | 4.30E-14 |
| ZFAS1 | hsa05163: Human cytomegalovirus infection | FAS, BAX, CDKN2A, MAPK14, HRAS, IL6R, CXCL8, KRAS, MYC, NRAS, MAPK1, RAF1, TNF, VEGFA | 1.57E-13 |
| ZFAS1 | hsa05161: Hepatitis B | FAS, BAX, BCL2, BRAF, MAPK14, HRAS, CXCL8, KRAS, MYC, NRAS, MAPK1, RAF1, TGFB1, TNF, DDX58 | 4.09E-17 |
| ZFAS1 | hsa05160: Hepatitis C | FAS, BAX, BRAF, HRAS, KRAS, LDLR, MYC, NRAS, MAPK1, RAF1, TNF, DDX58 | 1.08E-12 |
| ZFAS1 | hsa04010: MAPK signaling pathway | FAS, BRAF, MAPK14, FGFR3, HRAS, IGF1R, KRAS, MAX, MAP3K11, MYC, NRAS, MAPK1, RAF1, TGFB1, TNF, VEGFA | 1.38E-14 |
| ZFAS1 | hsa01522: Endocrine resistance | BAX, BCL2, BRAF, CDKN2A, MAPK14, ESR1, ESR2, HRAS, IGF1R, KRAS, NRAS, MAPK1, RAF1 | 6.46E-17 |
| ZFAS1 | hsa01521: EGFR tyrosine kinase inhibitor resistance | BAX, BCL2, BRAF, FGFR3, HRAS, IGF1R, IL6R, KRAS, NRAS, MAPK1, RAF1, VEGFA | 2.25E-16 |
| ZNF295-AS1 | hsa05219: Bladder cancer | BRAF, CDKN2A, FGFR3, HRAS, CXCL8, KRAS, MYC, NRAS, MAPK1, RAF1, VEGFA | 5.12E-18 |
| ZNF295-AS1 | hsa05205: Proteoglycans in cancer | FAS, BRAF, MAPK14, ESR1, HRAS, IGF1R, KRAS, MYC, NRAS, MAPK1, RAF1, TGFB1, TNF, VEGFA | 4.30E-14 |
| ZNF295-AS1 | hsa05163: Human cytomegalovirus infection | FAS, BAX, CDKN2A, MAPK14, HRAS, IL6R, CXCL8, KRAS, MYC, NRAS, MAPK1, RAF1, TNF, VEGFA | 1.57E-13 |
| ZNF295-AS1 | hsa04010: MAPK signaling pathway | FAS, BRAF, MAPK14, FGFR3, HRAS, IGF1R, KRAS, MAX, MAP3K11, MYC, NRAS, MAPK1, RAF1, TGFB1, TNF, VEGFA | 1.38E-14 |
| ZNF295-AS1 | hsa01521: EGFR tyrosine kinase inhibitor resistance | BAX, BCL2, BRAF, FGFR3, HRAS, IGF1R, IL6R, KRAS, NRAS, MAPK1, RAF1, VEGFA | 2.25E-16 |
| ZNF436-AS1 | hsa04010: MAPK signaling pathway | FAS, BRAF, MAPK14, FGFR3, HRAS, IGF1R, KRAS, MAX, MAP3K11, MYC, NRAS, MAPK1, RAF1, TGFB1, TNF, VEGFA | 1.38E-14 |
| ZNF561-AS1 | hsa05163: Human cytomegalovirus infection | FAS, BAX, CDKN2A, MAPK14, HRAS, IL6R, CXCL8, KRAS, MYC, NRAS, MAPK1, RAF1, TNF, VEGFA | 1.57E-13 |
| ZNF561-AS1 | hsa05161: Hepatitis B | FAS, BAX, BCL2, BRAF, MAPK14, HRAS, CXCL8, KRAS, MYC, NRAS, MAPK1, RAF1, TGFB1, TNF, DDX58 | 4.09E-17 |
| ZNF561-AS1 | hsa05160: Hepatitis C | FAS, BAX, BRAF, HRAS, KRAS, LDLR, MYC, NRAS, MAPK1, RAF1, TNF, DDX58 | 1.08E-12 |
| ZNF561-AS1 | hsa01522: Endocrine resistance | BAX, BCL2, BRAF, CDKN2A, MAPK14, ESR1, ESR2, HRAS, IGF1R, KRAS, NRAS, MAPK1, RAF1 | 6.46E-17 |
| ZNF561-AS1 | hsa01521: EGFR tyrosine kinase inhibitor resistance | BAX, BCL2, BRAF, FGFR3, HRAS, IGF1R, IL6R, KRAS, NRAS, MAPK1, RAF1, VEGFA | 2.25E-16 |
| ZNF582-AS1 | hsa05163: Human cytomegalovirus infection | FAS, BAX, CDKN2A, MAPK14, HRAS, IL6R, CXCL8, KRAS, MYC, NRAS, MAPK1, RAF1, TNF, VEGFA | 1.57E-13 |
| ZNF582-AS1 | hsa05161: Hepatitis B | FAS, BAX, BCL2, BRAF, MAPK14, HRAS, CXCL8, KRAS, MYC, NRAS, MAPK1, RAF1, TGFB1, TNF, DDX58 | 4.09E-17 |
| ZNF582-AS1 | hsa05160: Hepatitis C | FAS, BAX, BRAF, HRAS, KRAS, LDLR, MYC, NRAS, MAPK1, RAF1, TNF, DDX58 | 1.08E-12 |
| ZNF582-AS1 | hsa01522: Endocrine resistance | BAX, BCL2, BRAF, CDKN2A, MAPK14, ESR1, ESR2, HRAS, IGF1R, KRAS, NRAS, MAPK1, RAF1 | 6.46E-17 |
| ZNF582-AS1 | hsa01521: EGFR tyrosine kinase inhibitor resistance | BAX, BCL2, BRAF, FGFR3, HRAS, IGF1R, IL6R, KRAS, NRAS, MAPK1, RAF1, VEGFA | 2.25E-16 |
| ZNF674-AS1 | hsa05219: Bladder cancer | BRAF, CDKN2A, FGFR3, HRAS, CXCL8, KRAS, MYC, NRAS, MAPK1, RAF1, VEGFA | 5.12E-18 |
| ZNF674-AS1 | hsa05205: Proteoglycans in cancer | FAS, BRAF, MAPK14, ESR1, HRAS, IGF1R, KRAS, MYC, NRAS, MAPK1, RAF1, TGFB1, TNF, VEGFA | 4.30E-14 |
| ZNF674-AS1 | hsa05163: Human cytomegalovirus infection | FAS, BAX, CDKN2A, MAPK14, HRAS, IL6R, CXCL8, KRAS, MYC, NRAS, MAPK1, RAF1, TNF, VEGFA | 1.57E-13 |
| ZNF674-AS1 | hsa05161: Hepatitis B | FAS, BAX, BCL2, BRAF, MAPK14, HRAS, CXCL8, KRAS, MYC, NRAS, MAPK1, RAF1, TGFB1, TNF, DDX58 | 4.09E-17 |
| ZNF674-AS1 | hsa05160: Hepatitis C | FAS, BAX, BRAF, HRAS, KRAS, LDLR, MYC, NRAS, MAPK1, RAF1, TNF, DDX58 | 1.08E-12 |
| ZNF674-AS1 | hsa04010: MAPK signaling pathway | FAS, BRAF, MAPK14, FGFR3, HRAS, IGF1R, KRAS, MAX, MAP3K11, MYC, NRAS, MAPK1, RAF1, TGFB1, TNF, VEGFA | 1.38E-14 |
| ZNF674-AS1 | hsa01522: Endocrine resistance | BAX, BCL2, BRAF, CDKN2A, MAPK14, ESR1, ESR2, HRAS, IGF1R, KRAS, NRAS, MAPK1, RAF1 | 6.46E-17 |
| ZNF674-AS1 | hsa01521: EGFR tyrosine kinase inhibitor resistance | BAX, BCL2, BRAF, FGFR3, HRAS, IGF1R, IL6R, KRAS, NRAS, MAPK1, RAF1, VEGFA | 2.25E-16 |
| ZSCAN16-AS1 | hsa05219: Bladder cancer | BRAF, CDKN2A, FGFR3, HRAS, CXCL8, KRAS, MYC, NRAS, MAPK1, RAF1, VEGFA | 5.12E-18 |
| ZSCAN16-AS1 | hsa05205: Proteoglycans in cancer | FAS, BRAF, MAPK14, ESR1, HRAS, IGF1R, KRAS, MYC, NRAS, MAPK1, RAF1, TGFB1, TNF, VEGFA | 4.30E-14 |
| ZSCAN16-AS1 | hsa05163: Human cytomegalovirus infection | FAS, BAX, CDKN2A, MAPK14, HRAS, IL6R, CXCL8, KRAS, MYC, NRAS, MAPK1, RAF1, TNF, VEGFA | 1.57E-13 |
| ZSCAN16-AS1 | hsa05161: Hepatitis B | FAS, BAX, BCL2, BRAF, MAPK14, HRAS, CXCL8, KRAS, MYC, NRAS, MAPK1, RAF1, TGFB1, TNF, DDX58 | 4.09E-17 |
| ZSCAN16-AS1 | hsa05160: Hepatitis C | FAS, BAX, BRAF, HRAS, KRAS, LDLR, MYC, NRAS, MAPK1, RAF1, TNF, DDX58 | 1.08E-12 |
| ZSCAN16-AS1 | hsa04010: MAPK signaling pathway | FAS, BRAF, MAPK14, FGFR3, HRAS, IGF1R, KRAS, MAX, MAP3K11, MYC, NRAS, MAPK1, RAF1, TGFB1, TNF, VEGFA | 1.38E-14 |
| ZSCAN16-AS1 | hsa01522: Endocrine resistance | BAX, BCL2, BRAF, CDKN2A, MAPK14, ESR1, ESR2, HRAS, IGF1R, KRAS, NRAS, MAPK1, RAF1 | 6.46E-17 |
| ZSCAN16-AS1 | hsa01521: EGFR tyrosine kinase inhibitor resistance | BAX, BCL2, BRAF, FGFR3, HRAS, IGF1R, IL6R, KRAS, NRAS, MAPK1, RAF1, VEGFA | 2.25E-16 |
