# Supplementary material for: Competitive endogenous RNA network and pathway-based analysis of LncRNA single-nucleotide polymorphism in myasthenia gravis
Source: Sci Rep. 2021 Dec 14;11:23920. doi: 10.1038/s41598-021-03357-x (PMC8671434; doi:10.1038/s41598-021-03357-x)
Supplement: Supplementary file 8 — Supplementary Table S5. [file 41598_2021_3357_MOESM8_ESM.docx]

**Table S5. Detailed information of MG risk miRNAs**

| **Disease name** | **miRNA name** | **Detection method** | **Reference** |
| --- | --- | --- | --- |
| Myasthenia gravis | hsa-let-7a-5p^1-4^ | miRNA array, qRT-PCR etc. | 22835429 |
|  |  | miRNA array etc. | 23196978 |
|  |  | miRNA array etc. | 24734107 |
|  |  | qRT-PCR etc. | 26943954 |
| Myasthenia gravis | hsa-let-7b^1-3,5^ | miRNA array, qRT-PCR etc. | 22835429 |
|  |  | miRNA array etc. | 23196978 |
|  |  | miRNA array etc. | 24637658 |
|  |  | miRNA array etc. | 24734107 |
| Myasthenia gravis | hsa-let-7c^1-3^ | miRNA array, qRT-PCR etc. | 22835429 |
|  |  | miRNA array etc. | 23196978 |
|  |  | miRNA array etc. | 24734107 |
| Myasthenia gravis | hsa-let-7d-5p^1-3^ | miRNA array, qRT-PCR etc. | 22835429 |
|  |  | miRNA array etc. | 23196978 |
|  |  | miRNA array etc. | 24734107 |
| Myasthenia gravis | hsa-let-7d-3p^4^ | qRT-PCR etc. | 26943954 |
| Myasthenia gravis | hsa-let-7e^5^ | miRNA array etc. | 24637658 |
| Myasthenia gravis | hsa-let-7f-5p^1-4^ | miRNA array etc. | 22835429 |
|  |  | miRNA array etc. | 23196978 |
|  |  | miRNA array etc. | 24734107 |
|  |  | qRT-PCR etc. | 26943954 |
| Myasthenia gravis | hsa-let-7g^1,5^ | miRNA array etc. | 22835429 |
|  |  | miRNA array etc. | 24637658 |
| Myasthenia gravis | hsa-let-7i^1-3^ | miRNA array etc. | 22835429 |
|  |  | miRNA array etc. | 23196978 |
|  |  | miRNA array etc. | 24734107 |
| Myasthenia gravis | hsa-miR-106a-5p^6,7^ | miRNA array etc. | 29661539 |
|  |  | qRT-PCR etc. | 31889318 |
| Myasthenia gravis | hsa-miR-106b-5p^3^ | miRNA array etc. | 24734107 |
| Myasthenia gravis | hsa-miR-106b-3p^8,9^ | qRT-PCR etc. | 25356381 |
|  |  | qRT-PCR etc. | 29804819 |
| Myasthenia gravis | hsa-miR-107^1,3^ | miRNA array etc. | 22835429 |
|  |  | miRNA array etc. | 24734107 |
| Myasthenia gravis | hsa-miR-10a^3^ | miRNA array etc. | 24734107 |
| Myasthenia gravis | hsa-miR-10b-5p^4^ | qRT-PCR etc. | 26943954 |
| Myasthenia gravis | hsa-miR-122^5^ | miRNA array, qRT-PCR etc. | 24637658 |
| Myasthenia gravis | hsa-miR-1225-3p^3^ | miRNA array etc. | 24734107 |
| Myasthenia gravis | hsa-miR-1225-5p^1-3^ | miRNA array etc. | 22835429 |
|  |  | miRNA array etc. | 23196978 |
|  |  | miRNA array etc. | 24734107 |
| Myasthenia gravis | hsa-miR-1228^3^ | miRNA array etc. | 24734107 |
| Myasthenia gravis | hsa-miR-1234^3^ | miRNA array etc. | 24734107 |
| Myasthenia gravis | hsa-miR-1237^3^ | miRNA array etc. | 24734107 |
| Myasthenia gravis | hsa-miR-1238^3^ | miRNA array etc. | 24734107 |
| Myasthenia gravis | hsa-miR-1249^3^ | miRNA array etc. | 24734107 |
| Myasthenia gravis | hsa-miR-125a-3p^3^ | miRNA array etc. | 24734107 |
| Myasthenia gravis | hsa-miR-125a-5p^5,10,11^ | miRNA array etc. | 24637658 |
|  |  | miRNA array, qRT-PCR etc. | 26875774 |
|  |  | qRT-PCR etc. | 29655674 |
| Myasthenia gravis | hsa-miR-125b^3^ | miRNA array etc. | 24734107 |
| Myasthenia gravis | hsa-miR-1260^1-3^ | miRNA array etc. | 22835429 |
|  |  | miRNA array etc. | 23196978 |
|  |  | miRNA array etc. | 24734107 |
| Myasthenia gravis | hsa-miR-1267^3^ | miRNA array etc. | 24734107 |
| Myasthenia gravis | hsa-miR-1272^6^ | miRNA array etc. | 29661539 |
| Myasthenia gravis | hsa-miR-1273f^6^ | miRNA array etc. | 29661539 |
| Myasthenia gravis | hsa-miR-1274a^1,2^ | miRNA array etc. | 22835429 |
|  |  | miRNA array etc. | 23196978 |
| Myasthenia gravis | hsa-miR-1274b^1,2^ | miRNA array etc. | 22835429 |
|  |  | miRNA array etc. | 23196978 |
| Myasthenia gravis | hsa-miR-1277-5p^6^ | miRNA array etc. | 29661539 |
| Myasthenia gravis | hsa-miR-1280^3^ | miRNA array etc. | 24734107 |
| Myasthenia gravis | hsa-miR-1281^3^ | miRNA array etc. | 24734107 |
| Myasthenia gravis | hsa-miR-129-3p^1-3^ | miRNA array etc. | 22835429 |
|  |  | miRNA array etc. | 23196978 |
|  |  | miRNA array etc. | 24734107 |
| Myasthenia gravis | hsa-miR-130b-3p^5,8^ | miRNA array etc. | 24637658 |
|  |  | qRT-PCR etc. | 25356381 |
| Myasthenia gravis | hsa-miR-139-5p^1,12^ | miRNA array etc. | 22835429 |
|  |  | qRT-PCR etc. | 30308012 |
| Myasthenia gravis | hsa-miR-140-3p^4,5^ | miRNA array, qRT-PCR etc. | 24637658 |
|  |  | qRT-PCR etc. | 26943954 |
| Myasthenia gravis | hsa-miR-140-5p^5,9^ | miRNA array etc. | 24637658 |
|  |  | qRT-PCR etc. | 29804819 |
| Myasthenia gravis | hsa-miR-142-3p^1,3^ | miRNA array etc. | 22835429 |
|  |  | miRNA array etc. | 24734107 |
| Myasthenia gravis | hsa-miR-142-5p^3^ | miRNA array etc. | 24734107 |
| Myasthenia gravis | hsa-miR-143-3p^12^ | qRT-PCR etc. | 30308012 |
| Myasthenia gravis | hsa-miR-143-5p^12^ | qRT-PCR etc. | 30308012 |
| Myasthenia gravis | hsa-miR-144-3p^1,3^ | miRNA array etc. | 22835429 |
|  |  | miRNA array etc. | 24734107 |
| Myasthenia gravis | hsa-miR-144-5p^3^ | miRNA array etc. | 24734107 |
| Myasthenia gravis | hsa-miR-145^13^ | qRT-PCR etc. | 24043548 |
| Myasthenia gravis | hsa-miR-146a^14,15^ | qRT-PCR etc. | 24036458 |
|  |  | qRT-PCR etc. | 32210951 |
| Myasthenia gravis | hsa-miR-1470^3^ | miRNA array etc. | 24734107 |
| Myasthenia gravis | hsa-miR-148a-5p^6^ | miRNA array etc. | 29661539 |
| Myasthenia gravis | hsa-miR-149^3^ | miRNA array etc. | 24734107 |
| Myasthenia gravis | hsa-miR-150-5p^1-3,8,12,16-19^ | miRNA array etc. | 22835429 |
|  |  | miRNA array etc. | 23196978 |
|  |  | miRNA array etc. | 24734107 |
|  |  | qRT-PCR etc. | 25356381 |
|  |  | qRT-PCR etc. | 26095457 |
|  |  | qRT-PCR etc. | 30308012 |
|  |  | qRT-PCR etc. | 29511707 |
|  |  | qRT-PCR etc. | 30847357 |
|  |  | qRT-PCR etc. | 30984166 |
| Myasthenia gravis | hsa-miR-151a-3p^4^ | qRT-PCR etc. | 26943954 |
| Myasthenia gravis | hsa-miR-152-3p^12^ | qRT-PCR etc. | 30308012 |
| Myasthenia gravis | hsa-miR-1539^3^ | miRNA array etc. | 24734107 |
| Myasthenia gravis | hsa-miR-155^20^ | qRT-PCR etc. | 24387321 |
| Myasthenia gravis | hsa-miR-15a^1,21^ | miRNA array etc. | 22835429 |
|  |  | qRT-PCR etc. | 26845678 |
| Myasthenia gravis | hsa-miR-15b^1-3,5,22^ | miRNA array etc. | 22835429 |
|  |  | miRNA array etc. | 23196978 |
|  |  | miRNA array, qRT-PCR etc. | 24637658 |
|  |  | miRNA array etc. | 24734107 |
|  |  | qRT-PCR etc. | 26087886 |
| Myasthenia gravis | hsa-miR-16^1-3,5^ | miRNA array etc. | 22835429 |
|  |  | miRNA array etc. | 23196978 |
|  |  | miRNA array etc. | 24637658 |
|  |  | miRNA array etc. | 24734107 |
| Myasthenia gravis | hsa-miR-17^3^ | miRNA array etc. | 24734107 |
| Myasthenia gravis | hsa-miR-181a^23^ | qRT-PCR etc. | 31348521 |
| Myasthenia gravis | hsa-miR-181b^3^ | miRNA array etc. | 24734107 |
| Myasthenia gravis | hsa-miR-181c^24^ | qRT-PCR etc. | 25962782 |
| Myasthenia gravis | hsa-miR-1825^3^ | miRNA array etc. | 24734107 |
| Myasthenia gravis | hsa-miR-183^3^ | miRNA array etc. | 24734107 |
| Myasthenia gravis | hsa-miR-185^1-3,5^ | miRNA array etc. | 22835429 |
|  |  | miRNA array etc. | 23196978 |
|  |  | miRNA array, qRT-PCR etc. | 24637658 |
|  |  | miRNA array etc. | 24734107 |
| Myasthenia gravis | hsa-miR-188-5p^3^ | miRNA array etc. | 24734107 |
| Myasthenia gravis | hsa-miR-18b-3p^3^ | miRNA array etc. | 24734107 |
| Myasthenia gravis | hsa-miR-191-3p^3^ | miRNA array etc. | 24734107 |
| Myasthenia gravis | hsa-miR-192^5^ | miRNA array, qRT-PCR etc. | 24637658 |
| Myasthenia gravis | hsa-miR-1933-3p^25^ | qRT-PCR etc. | 30763589 |
| Myasthenia gravis | hsa-miR-193a^3^ | miRNA array etc. | 24734107 |
| Myasthenia gravis | hsa-miR-193a-5p^3,5,12^ | miRNA array etc. | 24637658 |
|  |  | miRNA array etc. | 24734107 |
|  |  | qRT-PCR etc. | 30308012 |
| Myasthenia gravis | hsa-miR-193b-3p^12^ | qRT-PCR etc. | 30308012 |
| Myasthenia gravis | hsa-miR-193b-5p^12^ | qRT-PCR etc. | 30308012 |
| Myasthenia gravis | hsa-miR-197^1-3^ | miRNA array etc. | 22835429 |
|  |  | miRNA array etc. | 23196978 |
|  |  | miRNA array etc. | 24734107 |
| Myasthenia gravis | hsa-miR-199a-3p^5^ | miRNA array etc. | 24637658 |
| Myasthenia gravis | hsa-miR-19b-3p^9^ | qRT-PCR etc. | 29804819 |
| Myasthenia gravis | hsa-miR-19b-5p^26^ | qRT-PCR etc. | 26531692 |
| Myasthenia gravis | hsa-miR-200c^3^ | miRNA array etc. | 24734107 |
| Myasthenia gravis | hsa-miR-202-3p^6^ | miRNA array etc. | 29661539 |
| Myasthenia gravis | hsa-miR-20a^1-3,5^ | miRNA array etc. | 22835429 |
|  |  | miRNA array etc. | 23196978 |
|  |  | miRNA array etc. | 24637658 |
|  |  | miRNA array etc. | 24734107 |
| Myasthenia gravis | hsa-miR-20b^1-3,5,27,28^ | miRNA array etc. | 22835429 |
|  |  | miRNA array etc. | 23196978 |
|  |  | miRNA array, qRT-PCR etc. | 24637658 |
|  |  | miRNA array etc. | 24734107 |
|  |  | qRT-PCR etc. | 26845056 |
|  |  | qRT-PCR etc. | 27833920 |
| Myasthenia gravis | hsa-miR-21^1^ | miRNA array etc. | 22835429 |
| Myasthenia gravis | hsa-miR-210^3,8^ | miRNA array etc. | 24734107 |
|  |  | qRT-PCR etc. | 25356381 |
| Myasthenia gravis | hsa-miR-214-3p^12^ | qRT-PCR etc. | 30308012 |
| Myasthenia gravis | hsa-miR-21-5p^8,16^ | qRT-PCR etc. | 25356381 |
|  |  | qRT-PCR etc. | 26095457 |
| Myasthenia gravis | hsa-miR-21g-3p^29^ | qRT-PCR etc. | 30316681 |
| Myasthenia gravis | hsa-miR-221^5^ | miRNA array etc. | 24637658 |
| Myasthenia gravis | hsa-miR-223-5p^9^ | qRT-PCR etc. | 29804819 |
| Myasthenia gravis | hsa-miR-22-3p^12^ | qRT-PCR etc. | 30308012 |
| Myasthenia gravis | hsa-miR-23a^1,3^ | miRNA array etc. | 22835429 |
|  |  | miRNA array etc. | 24734107 |
| Myasthenia gravis | hsa-miR-24-3p^5,12^ | miRNA array etc. | 24637658 |
|  |  | qRT-PCR etc. | 30308012 |
| Myasthenia gravis | hsa-miR-25-3p^1-5^ | miRNA array etc. | 22835429 |
|  |  | miRNA array etc. | 23196978 |
|  |  | miRNA array etc. | 24637658 |
|  |  | miRNA array etc. | 24734107 |
|  |  | qRT-PCR etc. | 26943954 |
| Myasthenia gravis | hsa-miR-26a^1^ | miRNA array etc. | 22835429 |
| Myasthenia gravis | hsa-miR-27a^1^ | miRNA array etc. | 22835429 |
| Myasthenia gravis | hsa-miR-296-5p^8^ | qRT-PCR etc. | 25356381 |
| Myasthenia gravis | hsa-miR-29a^1-3^ | miRNA array etc. | 22835429 |
|  |  | miRNA array etc. | 23196978 |
|  |  | miRNA array etc. | 24734107 |
| Myasthenia gravis | hsa-miR-29b^1-3^ | miRNA array etc. | 22835429 |
|  |  | miRNA array etc. | 23196978 |
|  |  | miRNA array etc. | 24734107 |
| Myasthenia gravis | hsa-miR-29c^5^ | miRNA array etc. | 24637658 |
| Myasthenia gravis | hsa-miR-30a-3p^12^ | qRT-PCR etc. | 30308012 |
| Myasthenia gravis | hsa-miR-30a-5p^3,12^ | miRNA array etc. | 24734107 |
|  |  | qRT-PCR etc. | 30308012 |
| Myasthenia gravis | hsa-miR-30e-5p^8,9,18^ | qRT-PCR etc. | 25356381 |
|  |  | qRT-PCR etc. | 29804819 |
|  |  | qRT-PCR etc. | 30847357 |
| Myasthenia gravis | hsa-miR-320a^2^ | miRNA array, qRT-PCR etc. | 23196978 |
| Myasthenia gravis | hsa-miR-320b^2^ | miRNA array etc. | 23196978 |
| Myasthenia gravis | hsa-miR-320d^3^ | miRNA array etc. | 24734107 |
| Myasthenia gravis | hsa-miR-323b-3p^30^ | qRT-PCR etc. | 31401213 |
| Myasthenia gravis | hsa-miR-324-3p^1,5,29^ | miRNA array etc. | 22835429 |
|  |  | miRNA array etc. | 24637658 |
|  |  | qRT-PCR etc. | 30316681 |
| Myasthenia gravis | hsa-miR-328-3p^29^ | qRT-PCR etc. | 30316681 |
| Myasthenia gravis | hsa-miR-331^5^ | miRNA array etc. | 24637658 |
| Myasthenia gravis | hsa-miR-338-3p^3^ | miRNA array etc. | 24734107 |
| Myasthenia gravis | hsa-miR-342-3p^3^ | miRNA array etc. | 24734107 |
| Myasthenia gravis | hsa-miR-345^5^ | miRNA array etc. | 24637658 |
| Myasthenia gravis | hsa-miR-34a-5p^8^ | qRT-PCR etc. | 25356381 |
| Myasthenia gravis | hsa-miR-362-3p^6^ | miRNA array etc. | 29661539 |
| Myasthenia gravis | hsa-miR-362-5p^3^ | miRNA array etc. | 24734107 |
| Myasthenia gravis | hsa-miR-363-3p^1-3,8^ | miRNA array etc. | 22835429 |
|  |  | miRNA array etc. | 23196978 |
|  |  | miRNA array etc. | 24734107 |
|  |  | qRT-PCR etc. | 25356381 |
| Myasthenia gravis | hsa-miR-365^1-3^ | miRNA array etc. | 22835429 |
|  |  | miRNA array etc. | 23196978 |
|  |  | miRNA array etc. | 24734107 |
| Myasthenia gravis | hsa-miR-3651^31^ | qRT-PCR etc. | 27387891 |
| Myasthenia gravis | hsa-miR-3654^31^ | qRT-PCR etc. | 27387891 |
| Myasthenia gravis | hsa-miR-369-3p^6^ | miRNA array etc. | 29661539 |
| Myasthenia gravis | hsa-miR-375^5^ | miRNA array etc. | 24637658 |
| Myasthenia gravis | hsa-miR-376c-3p^4^ | qRT-PCR etc. | 26943954 |
| Myasthenia gravis | hsa-miR-379-5p^6^ | miRNA array etc. | 29661539 |
| Myasthenia gravis | hsa-miR-409-3p^4,30^ | qRT-PCR etc. | 26943954 |
|  |  | qRT-PCR etc. | 31401213 |
| Myasthenia gravis | hsa-miR-421^4,8^ | qRT-PCR etc. | 25356381 |
|  |  | qRT-PCR etc. | 26943954 |
| Myasthenia gravis | hsa-miR-423-3p^3^ | miRNA array etc. | 24734107 |
| Myasthenia gravis | hsa-miR-423-5p^4^ | qRT-PCR etc. | 26943954 |
| Myasthenia gravis | hsa-miR-424-5p^8^ | qRT-PCR etc. | 25356381 |
| Myasthenia gravis | hsa-miR-425-5p^5^ | miRNA array etc. | 24637658 |
| Myasthenia gravis | hsa-miR-4442^6^ | miRNA array etc. | 29661539 |
| Myasthenia gravis | hsa-miR-451^1,3^ | miRNA array etc. | 22835429 |
|  |  | miRNA array etc. | 24734107 |
| Myasthenia gravis | hsa-miR-452-5p^12^ | qRT-PCR etc. | 30308012 |
| Myasthenia gravis | hsa-miR-484^4^ | qRT-PCR etc. | 26943954 |
| Myasthenia gravis | hsa-miR-485-3p^30^ | qRT-PCR etc. | 31401213 |
| Myasthenia gravis | hsa-miR-486-5p^1-3,5^ | miRNA array etc. | 24637658 |
|  |  | miRNA array etc. | 22835429 |
|  |  | miRNA array etc. | 23196978 |
|  |  | miRNA array etc. | 24734107 |
| Myasthenia gravis | hsa-miR-487a^6^ | miRNA array etc. | 29661539 |
| Myasthenia gravis | hsa-miR-494^1-3^ | miRNA array etc. | 22835429 |
|  |  | miRNA array etc. | 23196978 |
|  |  | miRNA array etc. | 24734107 |
| Myasthenia gravis | hsa-miR-495^6^ | miRNA array etc. | 29661539 |
| Myasthenia gravis | hsa-miR-518d^5^ | miRNA array etc. | 24637658 |
| Myasthenia gravis | hsa-miR-520d-5p^5^ | miRNA array etc. | 24637658 |
| Myasthenia gravis | hsa-miR-523^5^ | miRNA array etc. | 24637658 |
| Myasthenia gravis | hsa-miR-532-5p^3^ | miRNA array etc. | 24734107 |
| Myasthenia gravis | hsa-miR-548a^5^ | miRNA array etc. | 24637658 |
| Myasthenia gravis | hsa-miR-548b-5p^6^ | miRNA array etc. | 29661539 |
| Myasthenia gravis | hsa-miR-548c^5^ | miRNA array etc. | 24637658 |
| Myasthenia gravis | hsa-miR-548c-5p^6^ | miRNA array etc. | 29661539 |
| Myasthenia gravis | hsa-miR-548k^6^ | miRNA array, qRT-PCR etc. | 29661539 |
| Myasthenia gravis | hsa-miR-553^6^ | miRNA array etc. | 29661539 |
| Myasthenia gravis | hsa-miR-563^3^ | miRNA array etc. | 24734107 |
| Myasthenia gravis | hsa-miR-564^3,6^ | miRNA array etc. | 24734107 |
|  |  | miRNA array etc. | 29661539 |
| Myasthenia gravis | hsa-miR-570-5p^6^ | miRNA array etc. | 29661539 |
| Myasthenia gravis | hsa-miR-574-3p^3^ | miRNA array etc. | 24734107 |
| Myasthenia gravis | hsa-miR-584^3^ | miRNA array etc. | 24734107 |
| Myasthenia gravis | hsa-miR-601^6^ | miRNA array etc. | 29661539 |
| Myasthenia gravis | hsa-miR-602^1-3^ | miRNA array etc. | 22835429 |
|  |  | miRNA array etc. | 23196978 |
|  |  | miRNA array etc. | 24734107 |
| Myasthenia gravis | hsa-miR-610^6^ | miRNA array etc. | 29661539 |
| Myasthenia gravis | hsa-miR-612^31^ | qRT-PCR etc. | 27387891 |
| Myasthenia gravis | hsa-miR-629^3^ | miRNA array etc. | 24734107 |
| Myasthenia gravis | hsa-miR-634^1-3^ | miRNA array etc. | 22835429 |
|  |  | miRNA array etc. | 23196978 |
|  |  | miRNA array etc. | 24734107 |
| Myasthenia gravis | hsa-miR-652-3p^5,12^ | miRNA array etc. | 24637658 |
|  |  | qRT-PCR etc. | 30308012 |
| Myasthenia gravis | hsa-miR-665^3^ | miRNA array etc. | 24734107 |
| Myasthenia gravis | hsa-miR-720^1-3^ | miRNA array etc. | 22835429 |
|  |  | miRNA array etc. | 23196978 |
|  |  | miRNA array etc. | 24734107 |
| Myasthenia gravis | hsa-miR-7-5p^11^ | qRT-PCR etc. | 29655674 |
| Myasthenia gravis | hsa-miR-766^3^ | miRNA array etc. | 24734107 |
| Myasthenia gravis | hsa-miR-770-5p^6^ | miRNA array etc. | 29661539 |
| Myasthenia gravis | hsa-miR-885-5p^5^ | miRNA array, qRT-PCR etc. | 24637658 |
| Myasthenia gravis | hsa-miR-891a^6^ | miRNA array etc. | 29661539 |
| Myasthenia gravis | hsa-miR-92a^1-3^ | miRNA array etc. | 22835429 |
|  |  | miRNA array etc. | 23196978 |
|  |  | miRNA array etc. | 24734107 |
| Myasthenia gravis | hsa-miR-92b^3^ | miRNA array etc. | 24734107 |
| Myasthenia gravis | hsa-miR-93^1-3,5^ | miRNA array etc. | 22835429 |
|  |  | miRNA array etc. | 23196978 |
|  |  | miRNA array etc. | 24637658 |
|  |  | miRNA array etc. | 24734107 |
| Myasthenia gravis | hsa-miR-933^1-3^ | miRNA array etc. | 22835429 |
|  |  | miRNA array etc. | 23196978 |
|  |  | miRNA array etc. | 24734107 |
| Myasthenia gravis | hsa-miR-940^3^ | miRNA array etc. | 24734107 |

**Reference:**

1 Jiang, L. *et al.* Altered let-7 expression in Myasthenia gravis and let-7c mediated regulation of IL-10 by directly targeting IL-10 in Jurkat cells. *Int Immunopharmacol* **14**, 217-223, doi:10.1016/j.intimp.2012.07.003 (2012).

2 Cheng, Z. *et al.* MiR-320a is downregulated in patients with myasthenia gravis and modulates inflammatory cytokines production by targeting mitogen-activated protein kinase 1. *J Clin Immunol* **33**, 567-576, doi:10.1007/s10875-012-9834-5 (2013).

3 Jiang, C. *et al.* Clinical Study of Effects of Jian Ji Ning, a Chinese Herbal Medicine Compound Preparation, in Treating Patients with Myasthenia Gravis via the Regulation of Differential MicroRNAs Expression in Serum. *Evid Based Complement Alternat Med* **2014**, 518942, doi:10.1155/2014/518942 (2014).

4 Punga, T. *et al.* Disease specific enrichment of circulating let-7 family microRNA in MuSK+ myasthenia gravis. *J Neuroimmunol* **292**, 21-26, doi:10.1016/j.jneuroim.2016.01.003 (2016).

5 Nogales-Gadea, G. *et al.* Analysis of serum miRNA profiles of myasthenia gravis patients. *PLoS One* **9**, e91927, doi:10.1371/journal.pone.0091927 (2014).

6 Li, J. *et al.* miR-548k regulates CXCL13 expression in myasthenia gravis patients with thymic hyperplasia and in Jurkat cells. *J Neuroimmunol* **320**, 125-132, doi:10.1016/j.jneuroim.2018.03.021 (2018).

7 Xu, H. *et al.* Plasma exosomal miR-106a-5p expression in myasthenia gravis. *Muscle Nerve* **61**, 401-407, doi:10.1002/mus.26785 (2020).

8 Punga, T. *et al.* Circulating miRNAs in myasthenia gravis: miR-150-5p as a new potential biomarker. *Ann Clin Transl Neurol* **1**, 49-58, doi:10.1002/acn3.24 (2014).

9 Sabre, L., Maddison, P., Sadalage, G., Ambrose, P. A. & Punga, A. R. Circulating microRNA miR-21-5p, miR-150-5p and miR-30e-5p correlate with clinical status in late onset myasthenia gravis. *J Neuroimmunol* **321**, 164-170, doi:10.1016/j.jneuroim.2018.05.003 (2018).

10 Li, J. *et al.* Altered expression of miR-125a-5p in thymoma-associated myasthenia gravis and its down-regulation of foxp3 expression in Jurkat cells. *Immunol Lett* **172**, 47-55, doi:10.1016/j.imlet.2016.02.005 (2016).

11 Cron, M. A. *et al.* Analysis of microRNA expression in the thymus of Myasthenia Gravis patients opens new research avenues. *Autoimmun Rev* **17**, 588-600, doi:10.1016/j.autrev.2018.01.008 (2018).

12 Sengupta, M. *et al.* MicroRNA and mRNA expression associated with ectopic germinal centers in thymus of myasthenia gravis. *PLoS One* **13**, e0205464, doi:10.1371/journal.pone.0205464 (2018).

13 Wang, J. *et al.* Identification of novel MicroRNA signatures linked to experimental autoimmune myasthenia gravis pathogenesis: down-regulated miR-145 promotes pathogenetic Th17 cell response. *J Neuroimmune Pharmacol* **8**, 1287-1302, doi:10.1007/s11481-013-9498-9 (2013).

14 Lu, J. *et al.* Altered expression of miR-146a in myasthenia gravis. *Neurosci Lett* **555**, 85-90, doi:10.1016/j.neulet.2013.09.014 (2013).

15 Bortone, F. *et al.* miR-146a in Myasthenia Gravis Thymus Bridges Innate Immunity With Autoimmunity and Is Linked to Therapeutic Effects of Corticosteroids. *Front Immunol* **11**, 142, doi:10.3389/fimmu.2020.00142 (2020).

16 Punga, A. R., Andersson, M., Alimohammadi, M. & Punga, T. Disease specific signature of circulating miR-150-5p and miR-21-5p in myasthenia gravis patients. *J Neurol Sci* **356**, 90-96, doi:10.1016/j.jns.2015.06.019 (2015).

17 Molin, C. J., Sabre, L., Weis, C. A., Punga, T. & Punga, A. R. Thymectomy lowers the myasthenia gravis biomarker miR-150-5p. *Neurol Neuroimmunol Neuroinflamm* **5**, e450, doi:10.1212/NXI.0000000000000450 (2018).

18 Sabre, L. *et al.* miR-30e-5p as predictor of generalization in ocular myasthenia gravis. *Ann Clin Transl Neurol* **6**, 243-251, doi:10.1002/acn3.692 (2019).

19 Cron, M. A. *et al.* Causes and Consequences of miR-150-5p Dysregulation in Myasthenia Gravis. *Front Immunol* **10**, 539, doi:10.3389/fimmu.2019.00539 (2019).

20 Wang, Y. Z. *et al.* Delivery of an miR155 inhibitor by anti-CD20 single-chain antibody into B cells reduces the acetylcholine receptor-specific autoantibodies and ameliorates experimental autoimmune myasthenia gravis. *Clin Exp Immunol* **176**, 207-221, doi:10.1111/cei.12265 (2014).

21 Liu, X. F. *et al.* MiR-15a contributes abnormal immune response in myasthenia gravis by targeting CXCL10. *Clin Immunol* **164**, 106-113, doi:10.1016/j.clim.2015.12.009 (2016).

22 Shi, L. *et al.* miR-15b is Downregulated in Myasthenia Gravis Patients and Directly Regulates the Expression of Interleukin-15 (IL-15) in Experimental Myasthenia Gravis Mice. *Med Sci Monit* **21**, 1774-1780, doi:10.12659/MSM.893458 (2015).

23 Liu, X. *et al.* MiR-181a regulates CD4(+) T cell activation and differentiation by targeting IL-2 in the pathogenesis of myasthenia gravis. *Eur J Immunol*, doi:10.1002/eji.201848007 (2019).

24 Zhang, Y. *et al.* Decreased microRNA miR-181c expression in peripheral blood mononuclear cells correlates with elevated serum levels of IL-7 and IL-17 in patients with myasthenia gravis. *Clin Exp Med* **16**, 413-421, doi:10.1007/s10238-015-0358-1 (2016).

25 Bogatikov, E., Lindblad, I., Punga, T. & Punga, A. R. miR-1933-3p is upregulated in skeletal muscles of MuSK+ EAMG mice and affects Impa1 and Mrpl27. *Neurosci Res* **151**, 46-52, doi:10.1016/j.neures.2019.02.003 (2020).

26 Wang, Z. *et al.* Aberrant decrease of microRNA19b regulates TSLP expression and contributes to Th17 cells development in myasthenia gravis related thymomas. *J Neuroimmunol* **288**, 34-39, doi:10.1016/j.jneuroim.2015.08.013 (2015).

27 Chunjie, N., Huijuan, N., Zhao, Y., Jianzhao, W. & Xiaojian, Z. Disease-specific signature of serum miR-20b and its targets IL-8 and IL-25, in myasthenia gravis patients. *Eur Cytokine Netw* **26**, 61-66, doi:10.1684/ecn.2015.0367 (2015).

28 Xin, Y. *et al.* miR-20b Inhibits T Cell Proliferation and Activation via NFAT Signaling Pathway in Thymoma-Associated Myasthenia Gravis. *Biomed Res Int* **2016**, 9595718, doi:10.1155/2016/9595718 (2016).

29 Sabre, L. *et al.* Circulating microRNA plasma profile in MuSK+ myasthenia gravis. *J Neuroimmunol* **325**, 87-91, doi:10.1016/j.jneuroim.2018.10.003 (2018).

30 Cavalcante, P. *et al.* MicroRNA signature associated with treatment response in myasthenia gravis: A further step towards precision medicine. *Pharmacol Res* **148**, 104388, doi:10.1016/j.phrs.2019.104388 (2019).

31 Barzago, C. *et al.* A novel infection- and inflammation-associated molecular signature in peripheral blood of myasthenia gravis patients. *Immunobiology* **221**, 1227-1236, doi:10.1016/j.imbio.2016.06.012 (2016).
